# Activation and C−C Coupling of Aryl Iodides via Bismuth Photocatalysis

**DOI:** 10.1002/anie.202418367

**Published:** 2024-11-16

**Authors:** Mauro Mato, Alexios Stamoulis, Paolo Cleto Bruzzese, Josep Cornella

**Affiliations:** ^1^ Max-Planck-Institut für Kohlenforschung Kaiser-Wilhelm-Platz 1 45470 Mülheim an der Ruhr Germany; ^2^ Max-Planck-Institut für Chemische Energiekonversion Stiftstrasse 34–36 45470 Mülheim an der Ruhr Germany

**Keywords:** bismuth catalysis, radical catalysis, photocatalysis, cross-coupling reactions, aryl halide activation

## Abstract

Within the emerging field of bismuth redox catalysis, the catalytic formation of C−C bonds using aryl halides would be highly desirable; yet such a process remains a synthetic challenge. Herein, we present a chemoselective bismuth‐photocatalyzed activation and subsequent coupling of (hetero)aryl iodides with pyrrole derivatives to access C(sp^2^)−C(sp^2^) linkages through C−H functionalization. This unique reactivity is the result of the bismuth complex featuring two redox state‐dependent interactions with light, which 1) activates the Bi(I) complex for oxidative addition via MLCT, and 2) promotes the homolytic cleavage of aryl Bi(III) intermediates through a LLCT process.

## Graphical abstract

The catalytic formation of C–C bonds from aryl halides is a highly attractive but unexplored area in the emerging field of bismuth redox catalysis. We report a chemoselective bismuth‐photocatalyzed activation and coupling of (hetero)aryl iodides with pyrrole derivatives to forge C(sp^2^)–C(sp^2^) bonds via C–H functionalization. The bismuth complex features two different light‐driven processes: an MLCT transition that promotes oxidative addition into Bi(I) and an LLCT‐enabled homolytic cleavage of aryl–Bi(III) bonds.

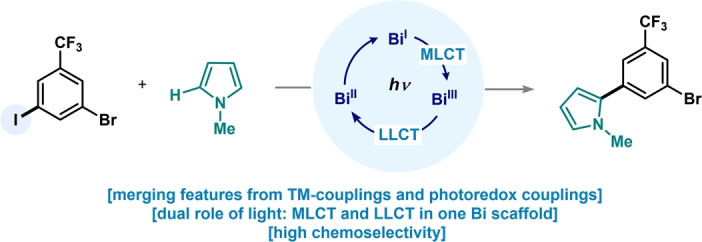

The formation of C−C bonds is among the most powerful tools available to synthetic chemists to construct molecular complexity. A successful approach to forge these bonds have been transition metal‐catalyzed cross‐coupling reactions between nucleophiles and electrophiles, which revolutionized the preparation of organic molecules in academic, medicinal, and industrial settings.[^1^, ^2^] Mechanistically, the metal complexes in these reactions are involved in three inner‐sphere organometallic processes, namely oxidative addition, transmetalation, and reductive elimination (Figure 1A, left).^[3]^ However, recent times have witnessed the development of alternative catalytic radical coupling reactions, thus giving rise to novel retrosynthetic disconnections.[^4^, ^5^] Unlike canonical TM‐catalyzed manifolds, these reactions typically proceed through single‐electron processes involving outer‐sphere mechanisms (Figure 1A, right).^[6]^ These developments arose hand‐in‐hand with the fast progress in photoredox catalysis, which provided a unique reactivity landscape.^[7]^ For example, various photocatalysts can be used to activate electrophilic organic (pseudo)halides via excited‐state single‐electron transfer (SET), generating free organic open‐shell species that can engage in downstream radical reactivity,[^8^, ^9^] or participate in subsequent cooperative catalytic cycles (e.g. metallaphotoredox).[^10^, ^11^]

**Figure 1:**
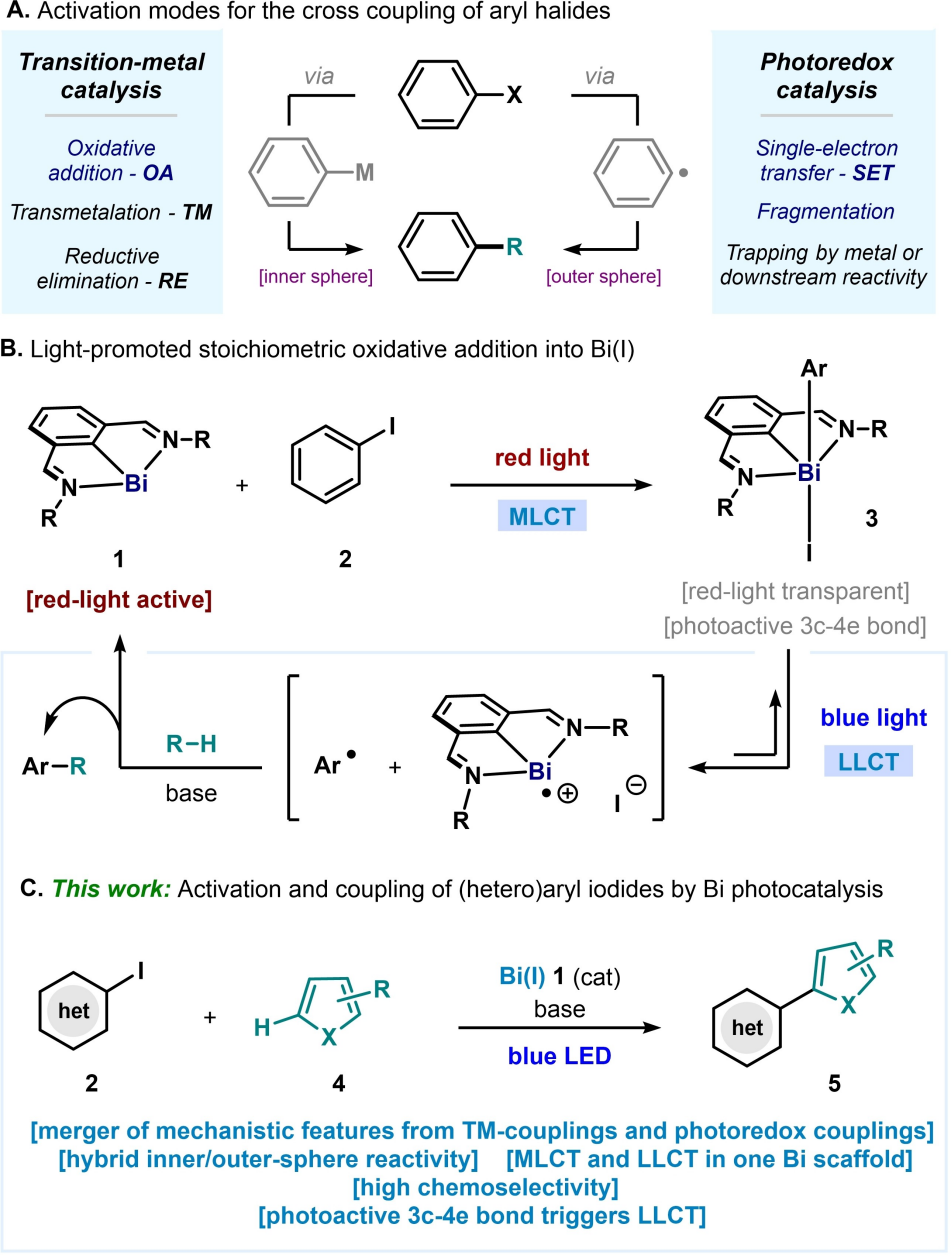
**(A)** Classical approaches for the catalytic activation of aryl halides. **(B)** Light‐promoted stoichiometric oxidative addition of aryl iodides into Bi(I). **(C)** This work: Photocatalytic activation and coupling of aryl iodides with bismuth, merging inner‐ and outer‐sphere mechanistic features.

Our group has been interested in the reactivity of bismuth complexes, in the pursuit of fundamentally unique catalysis. Indeed, bismuth is capable of participating in a rich variety of two‐electron and one‐electron[^12^, ^13^, ^14^] redox events that established the foundations for novel catalytic manifolds.[^15^, ^16^, ^17^, ^18^, ^19^, ^20^, ^21^, ^22^, ^23^, ^24^, ^25^] Very recently, we reported that *N,C,N*‐bismuthinidenes (general structure **1**, Figure 1B)[^26^, ^27^] represent a new family of photoactive species capable of undergoing light‐promoted oxidative additions with a variety of aryl electrophiles, giving access to well‐defined aryl‐Bi(III) complexes (Figure 1B).^[28]^ The use of low‐energy red light enabled a key metal‐to‐ligand charge transfer (MLCT) transition in **1**, while being innocuous to the resulting oxidative‐addition products **3**. However, the inertness of the resulting adducts **3** in the ground state precluded further reactivity.

Now, we report that the resulting aryl‐Bi(III) iodide complexes **3** can absorb blue light to access a ligand‐to‐ligand charge transfer (LLCT) transition, which triggers Bi−Ar homolysis and enables access to Ar radical chemistry (Figure 1B).[^18^, ^29^, ^30^] This reactivity can be harnessed in a catalytic C−H coupling event that combines features of traditional transition‐metal catalysis (well‐defined oxidative‐addition adduct intermediates) and photoredox couplings (outer‐sphere trapping of organic‐radical intermediates). This led to the development of a chemoselective photocatalytic cross‐coupling reaction between (hetero)aryl iodides and pyrroles,[^31^, ^32^, ^33^, ^34^, ^35^, ^36^, ^37^, ^38^, ^39^] where light plays a dual role in the activation of the Bi complexes via MLCT followed by LLCT (Figure 1C).[^10^, ^11^, ^40^, ^41^, ^42^]

We have previously reported that the dark green Bi(I) complex **1a** reacts with 4‐iodobenzonitrile (**2a**) under red‐light (660 nm) irradiation, leading to the clean formation of adduct **3a** (Figure 2, top left).^[28]^ In contrast, complex **3a** (yellow in solution) does not absorb low‐energy red photons (Figure 2, bottom left); however, upon blue‐light (457 nm) irradiation, **3a** reacts with an excess of *N*‐methylpyrrole (**4a**) in the presence of K_2_HPO_4_ to give coupling product **5a** in 69% yield after 24 h, with subsequent regeneration of Bi(I) complex **1a** (Figure 2, right). This reactivity suggests that light induces the homolytic cleavage of the Bi−C bond, affording a putative intermediate Bi(II)‐species **3aa** and aryl radical **3ab**.[^12^, ^13^, ^14^, ^43^] We postulate that the aryl radical can be trapped by **4a**, and the resulting adduct would deliver product **5a** after formal oxidation by **3aa** and elimination with base. The formation of the desired C−H arylation product and regeneration of the parent Bi(I) complex led us to explore the development of a catalytic version of this process. For this purpose, we hypothesized that blue light (which can be absorbed both by **1a** and **3a**; Figure 2, bottom left) could serve not only to promote the homolytic cleavage of **3a**, but also to excite Bi(I) **1a** to the same reactive MLCT state responsible for the activation of aryl iodides.^[28]^ Indeed, after 14 h of blue‐light irradiation, the reaction of **2a** (0.1 M) with 50 equivalents of **4a** in the presence of 10 mol% of Bi(I) **1a** and 2.0 equivalents of K_2_HPO_4_ in MeCN leads to the formation of coupling product **5a** in 38% yield (Table 1, entry 2). We believe the excess of **4a** is required to favor the desired reactivity over deleterious side‐reactions that occur when **3a** is irradiated with blue light for prolonged periods of time in the absence of a coupling partner (for a graphical representation of the persistence of **3a** under various wavelengths of light, see Figure S20 in the Supporting Information). We observed that by simply extending the reaction time, the product was obtained in 58% NMR yield after 48 h, or 62% isolated yield after 60 h (entry 1). Control experiments confirmed the need of light (entry 3), Bi(I) catalyst (entry 4) or base (entry 5). Screening other Bi(I) complexes as catalysts led to either similar (**1b**, entry 7) or more sluggish reactivity (**1c**, entry 8). Simple bismuth halides (entry 9) or *N,C,N*‐bismuth(III) dihalides **1aa** or **1ab** (entry 10) were not catalytically active.^[44]^

**Figure 2:**
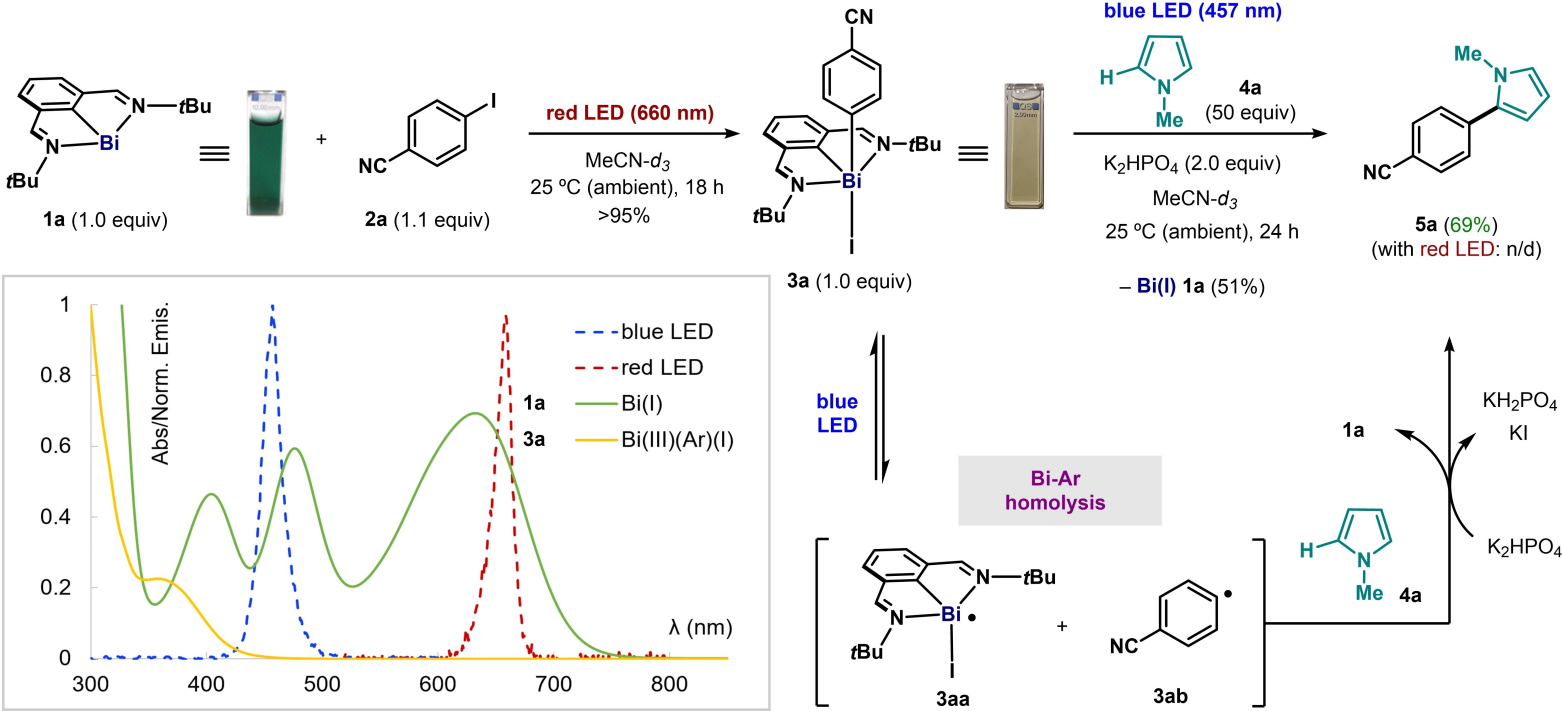
Photophysical properties (UV‐Vis absorption spectra; green trace: absorbance of **1a**; yellow trace: absorbance of **3a**; blue dashed trace: emission of blue LED; red dashed trace: emission of red LED), physical appearance and photochemical reactivity of Bi(I) **1a** and aryl oxidative‐addition Bi(III) adduct **3a** under red‐ or blue‐light irradiation.

**Table 1:**
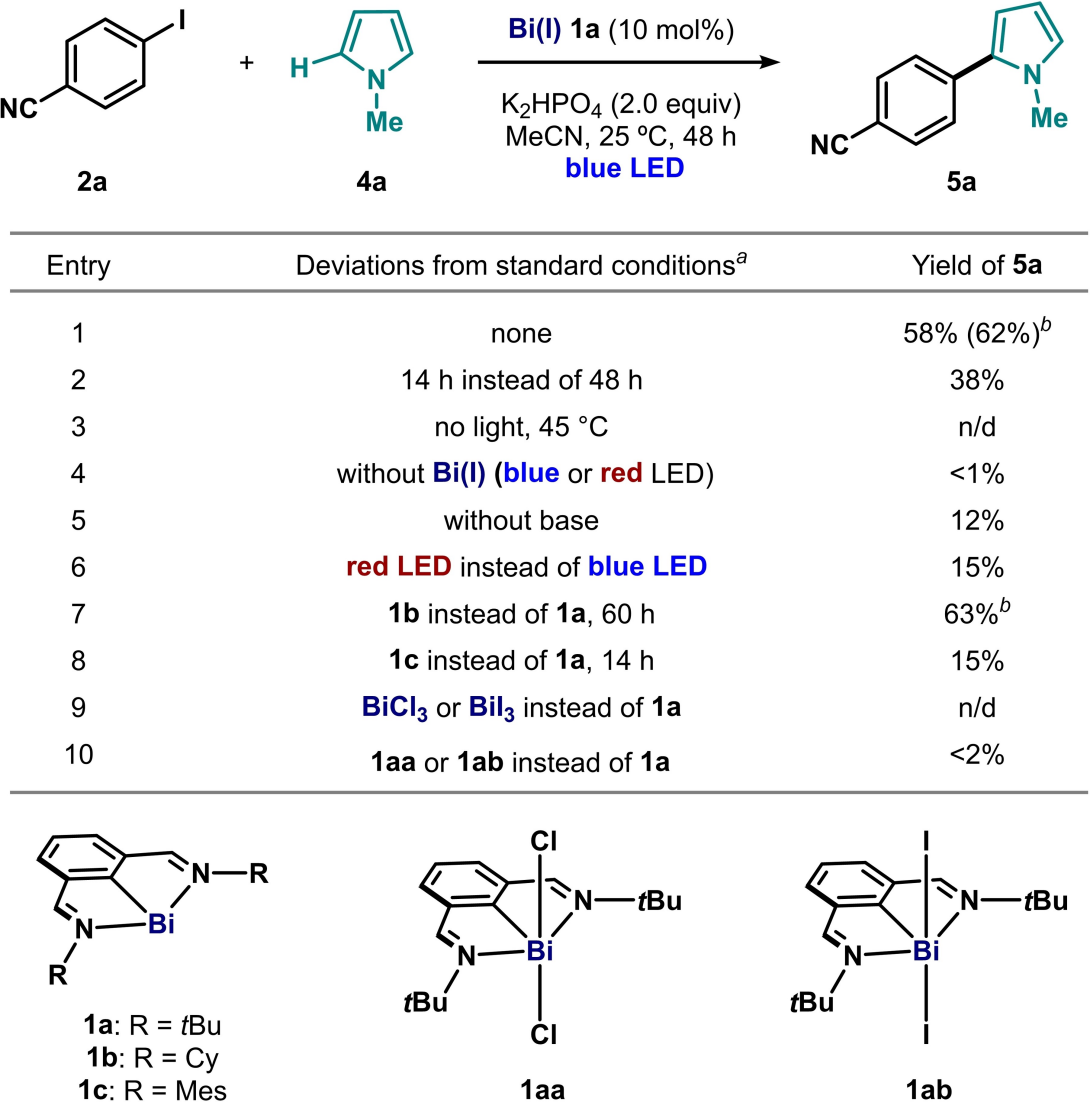
Optimization and control experiments for the bismuth‐catalyzed coupling.

[a] Reaction conditions: **2a** (0.05 mmol, 0.1 M, 1 equiv), **4a** (50 equiv), K_2_HPO_4_ (2 equiv), and **1a** (10 mol%) in MeCN with constant stirring and blue‐light irradiation (457 nm LED strip) at 25 °C (ambient) for 48 h. ^1^H NMR yields determined using Ph_2_CH_2_ as internal standard. [b] 60 h reaction time instead of 48 h. n/d: not detected.

With optimized conditions in hand, we interrogated the generality of the protocol (Table 2). Substituents at the 4‐, 3‐ and 2‐position of the aryl iodide were similarly tolerated, giving the corresponding products in moderate to good yields (**5a−c**). The reaction was found to display excellent chemoselectivity, even in the presence of other electrophilic moieties on the aryl iodide. For example, activation of the C(sp^2^)−I bond occurs exclusively even in the presence of bromide (**5e**, **5g**, **5k**, **5l**), chloride (**5f**, **5m**) and triflate (**5d**) substituents on the iodoarene. In line with our previous observations on the stoichiometric oxidative addition between **1a** and aryl iodides, electron‐rich electrophiles such as 4‐iodotoluene react much slower (**5q**).^[45]^ On the other hand, we found that relevant heteroaryl moieties, such as iodopyridines (**5h**, **5j**−**l**) behave exceedingly well, providing the corresponding coupling products in good yields, even in the presence of other halide substituents.[^46^, ^47^] Remarkably, iodopyrazine or iodobenzopyrazine delivered products **5i** and **5n** in excellent yield. We also performed control experiments without catalyst for high‐yielding products **5h** and **5i**, confirming the requirement of the bismuth complex in both cases. Besides **4a**, other unprotected pyrrole derivatives were evaluated, giving products **5o** and **5p** with similar efficiency. Additionally, other C‐H coupling partners also led to C−C adducts **5r**−**t** albeit in modest yields, suggesting the potential generalization to the C−H functionalization of other heterocycles.^[38]^

**Table 2:**
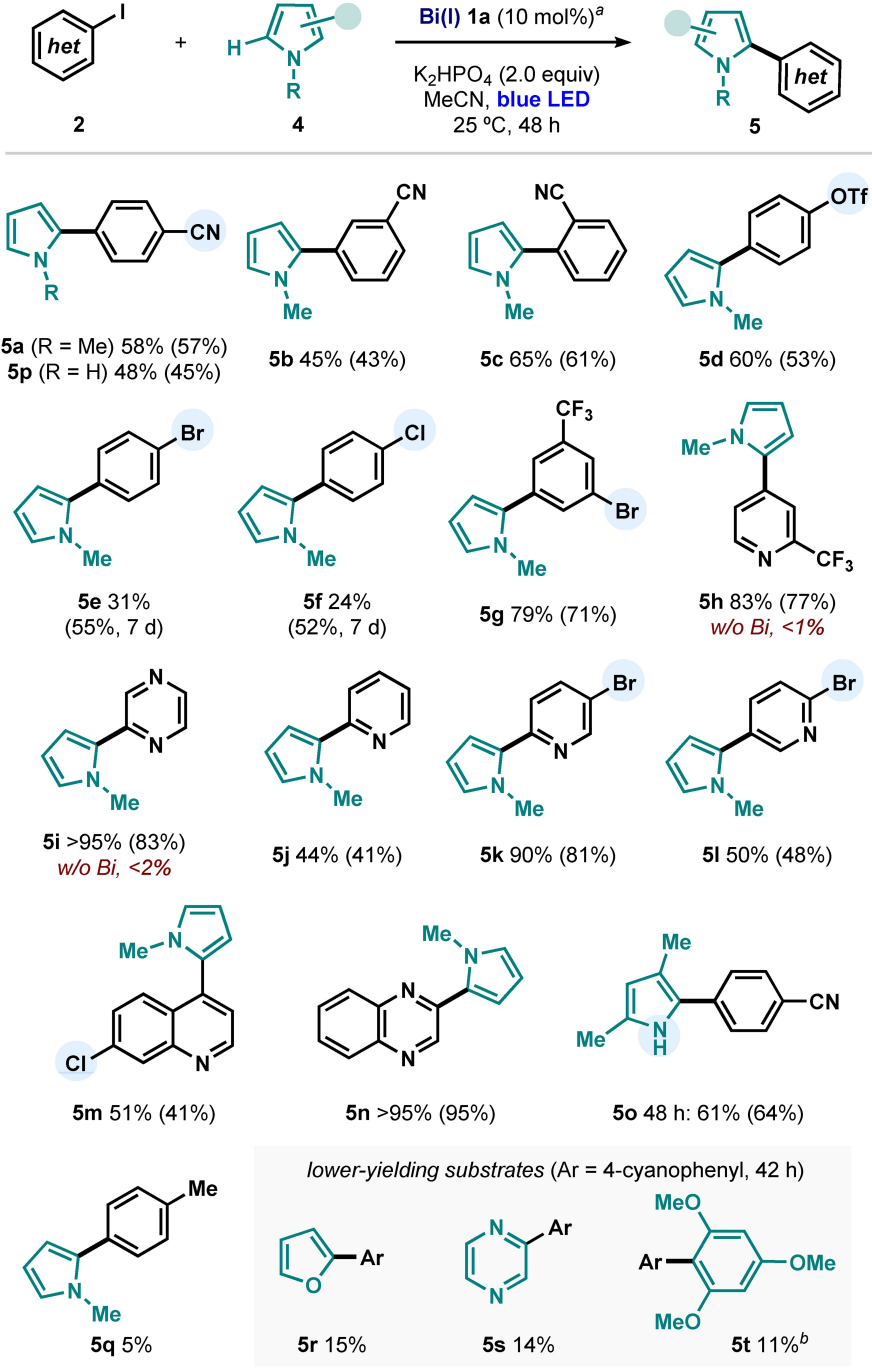
Scope of the bismuth‐photocatalyzed chemoselective cross coupling of aryl iodides with pyrroles and other substrates.

[a] Reaction conditions: **2** (0.2 mmol, 1 equiv), nucleophile **4** (50 equiv) with K_2_HPO_4_ (2 equiv) in the presence of Bi(I) **1a** (10 mol%) in MeCN (0.1 M) stirred upon blue‐light irradiation (457 nm LED strip) at 25 °C (ambient) for 60 h, unless stated otherwise. ^1^H NMR yields determined using Ph_2_CH_2_ as internal standard. Isolated yield between parentheses. [b] With 5 equiv of 1,3,5‐trimethoxybenzene.

During reaction development, we observed that bismuth(I) complex **1a** was not able to promote the analogous catalytic couplings with aryl diazonium salts (**6**). These highly oxidizing electrophiles react immediately with Bi(I), even in the dark, to give aryl bismuthonium complexes such as **3a−BF_4_** (Figure 3A).^[28]^ Surprisingly, subjecting this cationic complex to standard stoichiometric coupling conditions did not lead to the formation of any product **5a**. At this point, we speculated that the counterion might have a profound effect on the observed reactivity. Indeed, upon introduction of an iodide source such as NaI or tetrabutylammonium iodide (TBAI) the expected photoreactivity was restored, affording 42% and 44% yield of **5a**, respectively, after 16 h under blue‐LED irradiation. This striking counteranion‐dependence in the photoreactivity of these complexes prompted us to carefully analyze the role of the iodide. While **3a−BF_4_** does not absorb light above 400 nm, addition of 1.0 or 2.0 equivalents of TBAI led to the appearance of a new absorption band centered at 370 nm and tailing down to 450 nm.^[44]^ This new band (also present in **3a−I**, see Figures 2 or 3C) overlays with the emission band of the blue LED employed in this study. To shed light into the nature of this transition, we performed time‐dependent density functional theory (TDDFT) analysis using the ORCA package.^[48]^ Figure 3C shows the experimental absorption spectrum of pure **3a−I** (black trace) overlapped with the spin‐orbit coupling (SOC)‐corrected TDDFT‐predicted transitions (red bars). Analysis of the natural transition orbitals unveiled that the blue‐light absorptions correspond mainly to ligand‐to‐ligand charge transfer (LLCT) transitions,[^19^, ^29^, ^30^] where electron density is pushed from the axial 3c‐4e bond (HOMO, composed by the 5p_z_ orbital of the iodide and the σ orbitals of 4‐cyanophenyl) towards the *N,C,N* backbone (LUMO, antibonding π orbitals spread over the *N,C,N*‐pincer ligand). We believe this process facilitates the blue‐light‐promoted homolytic Bi−C cleavage of the axial aryl group, leading to the observed reactivity.

**Figure 3:**
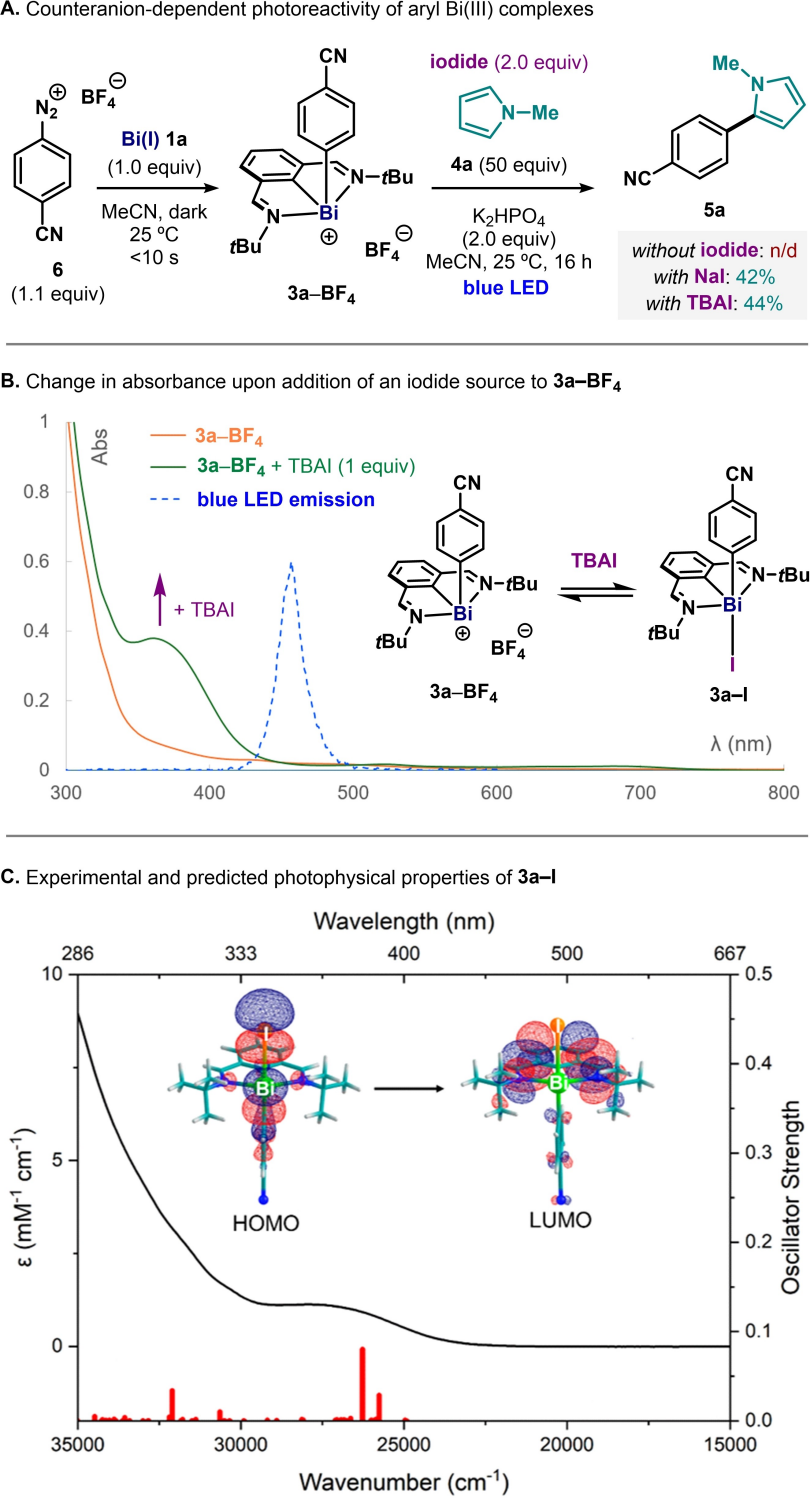
**(A)** Effect of the counteranion (I vs BF_4_) in the reactivity of **3a**. **(B)** Absorption difference of **3a–BF4** upon addition of TBAI (tetra‐*n*‐butylammonium iodide) and blue‐LED emission. **(C)** Experimental UV‐Vis spectrum of **3a–I** (black trace), SOC‐corrected TDDFT transitions (red bars), and key orbitals involved in LLCT blue‐light absorptions.

Notably, bond‐dissociation free energy (BDFE) analysis revealed the axial Bi–Ar bond (Ar = 4‐cyanophenyl) in **3a** to be considerably weaker (52.3 kcal/mol) than the Bi–I bond (66.0 kcal/mol), favoring the Bi–C homolysis upon LLCT.^[15]^ Finally, in accordance with experimental observations, a similar TDDFT calculation for complex **3a−BF4** (without iodide as second axial coordinating ligand) predicts no significant transitions above 350 nm (see Figure S23).^[44]^

With these observations in mind, we propose the mechanistic cycle shown in Figure 4A. The reaction would start by the known light‐promoted oxidative addition of aryl iodide **2a** into Bi(I) **1a**.[^28^, ^49^] Then, upon LLCT (Figure 3C), aryl‐Bi(III) iodide complex **3a** would be in equilibrium with the caged radical pair consisting of an aryl radical and Bi(II) species **3aa**.^[43]^ The aryl radical can be trapped by pyrrole **4a**, giving a highly reducing vinylogous α‐amino radical **6**.^[18]^ This reducing species would react readily with the relatively oxidizing Bi(II) **3aa** to give Bi(I) **1a** and coupling product **5a** upon rearomatization of the pyrrole with base.^[50]^

**Figure 4:**
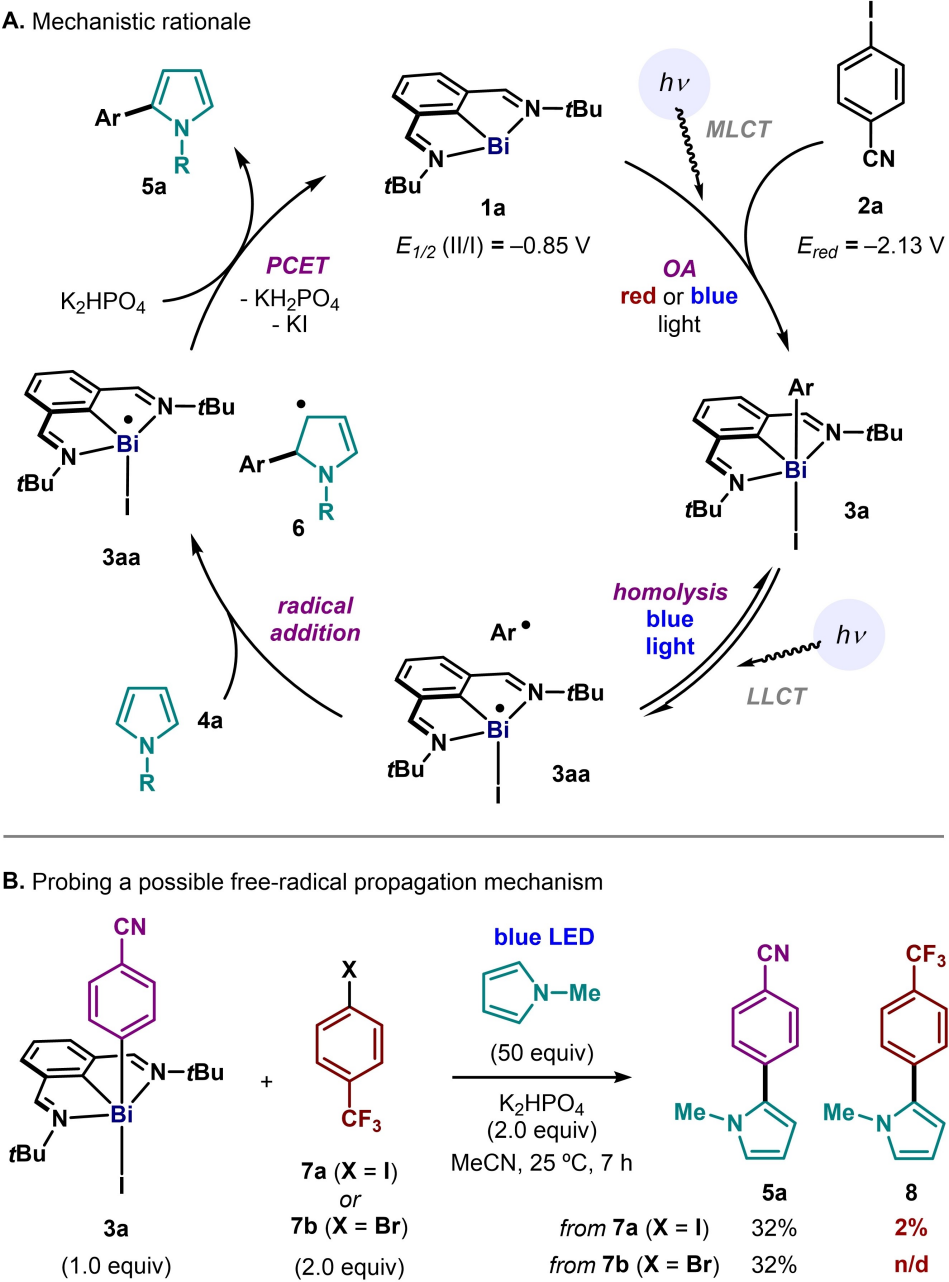
**(A)** Proposed mechanistic rationale. Redox potentials in V vs Fc^0/+^. OA: oxidative addition; PCET: proton‐coupled electron transfer; MLCT: metal‐to‐ligand charge transfer; LLCT: ligand‐to‐ligand charge transfer. **(B)** Mechanistic experiments evaluating the competence of a free‐radical propagation mechanism. n/d: not detected.

Previous reports have highlighted the feasibility of a free‐radical chain propagation mechanism when γ‐enamino radicals such as **6** are formed in a photochemical reaction.^[38]^ Hence, we sought to probe the existence of such propagation mechanism in our system, which would be initiated via halogen‐atom transfer (XAT) of radical **6** with free aryl iodide. First, reaction of 1 equivalent of freshly prepared adduct **3a** with *N*‐methylpyrrole in the presence of 2 equivalents of **7a** led to the formation of 32% of stoichiometric‐coupling product **5a** and only 2% of product **8** (Figure 4B) after 7 h of blue‐light irradiation. The latter could arise from either a free‐radical reaction of **6** with **7a** or by simple stoichiometric coupling between **7a** and pyrrole promoted by in situ‐generated Bi(I). In order to fully discriminate between the two scenarios, we performed the same experiment using aryl bromide **7b**, which could still participate in a chain propagation process with **6**,[^36^, ^37^, ^38^] but is not activated by Bi(I) **1a** under the reaction conditions. Indeed, this led to the exclusive formation of **5a** (32%), with no radical‐crossover product **8** detected. These observations, together with (i) the success of the stoichiometric reaction in the absence of aryl iodide (Figure 2), (ii) the qualitative alignment between the slow overall reaction kinetics with those of the isolated oxidative‐addition step,^[28]^ and (iii) the catalytic quantum yield values below 1%,^[44]^ are consistent with product formation occurring almost exclusively via a bismuth‐catalyzed mechanism as presented in Figure 4A. The absence of a free radical chain propagation mechanism is rationalized by a fast in‐cage collapse of the **3aa/6** pair. This is attributed to the relatively high oxidizing nature of Bi(II) **3aa** (E_1/2_(II/I) = − 0.85 V vs Fc^+/0^),[^18^, ^43^, ^44^, ^51^] which can be rapidly reduced by nucleophilic radical **6** before the latter can react with another molecule of aryl halide. As a consequence of this, our catalytic system exclusively activates aryl iodides over bromides or chlorides, the latter substrates being often indiscriminately reactive under free radical‐chain propagation conditions or related systems.[^34^, ^38^] Notably, these investigations were made possible by the unique behavior of bismuth complexes as enabling mechanistic tools that allow the isolation and study of photoreactive intermediates which are often not observable in traditional TM photoredox systems.^[51]^

In summary, we disclose a bismuth‐based approach for the activation and coupling of aryl iodides, which merges mechanistic features of traditional TM‐based cross‐coupling reactions and photoredox couplings. Beyond unlocking the elusive formation of C−C bonds via bismuth redox catalysis, we describe a unique photoredox system where light plays two distinct roles. First, the light‐promoted MLCT transition in the Bi(I) complex engenders the oxidative addition into aryl iodides. And second, the LLCT‐induced homolytic cleavage of otherwise unreactive aryl Bi(III) intermediates, for which we found a dramatic counteranion‐dependence on the photoreactivity. Furthermore, studies on photoactive reaction intermediates revealed that product formation almost exclusively arises from a Bi‐catalyzed mechanism instead of a radical chain propagation. Overall, this work represents an entry point for the use of low‐valent bismuth as a mechanistically atypical platform in the development of new photocatalyzed C−C coupling reactions.

## Supporting Information

The authors have cited additional references within the Supporting Information.[^18^, ^19^, ^27^, ^28^, ^48^, ^53^, ^54^, ^55^, ^56^, ^57^, ^58^, ^59^, ^60^, ^61^, ^62^, ^63^, ^64^, ^65^, ^66^, ^67^, ^68^, ^69^, ^70^, ^71^, ^72^, ^73^, ^74^]

## Conflict of Interests

The authors declare no conflict of interest.

## Supporting information

As a service to our authors and readers, this journal provides supporting information supplied by the authors. Such materials are peer reviewed and may be re‐organized for online delivery, but are not copy‐edited or typeset. Technical support issues arising from supporting information (other than missing files) should be addressed to the authors.

Supporting Information
