# Supplementary material for: Activation and C−C Coupling of Aryl Iodides via Bismuth Photocatalysis
Source: Angew Chem Int Ed Engl. 2024 Nov 16;64(5):e202418367. doi: 10.1002/anie.202418367 (PMC11773318; doi:10.1002/anie.202418367)
Supplement: Supplementary file 1 — Supporting Information [file ANIE-64-e202418367-s001.pdf]

Angewandte Chemie
Eine Zeitschrift der Gesellschaft Deutscher Chemiker

Supporting Information

Activation and C–C Coupling of Aryl Iodides via Bismuth Photocatalysis

*M. Mato, A. Stamoulis, P. Cleto Bruzzese, J. Cornella**

Supporting Information

Activation and C–C Coupling of Aryl Iodides via Bismuth Photocatalysis

Mauro Mato,[a] Alexios Stamoulis,[a] Paolo Cleto Bruzzese,[b] Josep Cornella[a,*]

[a] Max-Planck-Institut für Kohlenforschung, Kaiser-Wilhelm-Platz 1, Mülheim an der Ruhr, 45470, Germany. [b] Max-Planck-Institut für Chemische Energiekonversion, Stiftstrasse 34–36, Mülheim an der Ruhr, 45470, Germany

cornella@kofo.mpg.de

Supporting Information

1. General considerations 3

2. Light sources and photoreaction set-ups 4

3. Synthesis of bismuthinidenes 7

4. Stoichiometric aryl oxidative additions into bismuth(I) 8

  4.1. Red-light promoted oxidative addition of aryl iodides 8

  4.1. Oxidative addition of aryl diazonium salts 10

5. Reaction development, optimization and control experiments 13

6. Bismuth-photocatalyzed activation and coupling of aryl iodides 15

  6.1. General procedure for the coupling reactions 15

  6.2. Characterization data for the reaction products 16

  6.3. Unsuccessful or low-yielding substrates 25

  6.4. Miscellaneous information 28

7. Electrochemical data 31

8. Photophysical properties 33

  8.1. UV-Vis absorption and emission spectroscopy 33

  8.2. Ferrioxalate actinometry and quantum yield determination 41

9. Mechanistic experiments 45

  9.1. Effect of iodide on the stoichiometric C-H arylation of *N*-methyl pyrrole 45

  9.2. Stoichiometric experiments to differentiate between catalytic and radical chain pathways for product formation 46

  9.3. Miscellaneous experiments 47

10. Computational analysis 48

  10.1 TDDFT study 50

  10.2 Calculation of BDFE 52

  10.3 Cartesian coordinates 53

11. NMR spectra 61

12. References 88

1. General considerations

Unless otherwise stated, all manipulations were performed under argon using standard Schlenk-line techniques or in an argon-filled glovebox.

Instruments

NMR data were recorded using Bruker AVIII HD 300 MHz, Bruker AVIII HD 400 MHz, or Bruker AVNeo 600 MHz NMR spectrometers (at 298-300 K, unless stated otherwise). ^{1}H and ^{13}C chemical shifts are reported in ppm relative to the solvent residual peaks as an internal reference. For ^{1}H NMR the following residual proton peaks of the deuterated solvents were used: $CDCl_3$, $\delta_H(CHCl_3)$ 7.260; THF-d_8, $\delta_H((CD_2)_3CHDO)$ 3.580; CD_3CN, $\delta_H(CHD_2CN)$ 1.940. For ^{13}C NMR: $CDCl_3$, δ 77.16; THF-d_8, δ 67.57; CD_3CN, δ 1.32. ^{13}C spectra were acquired with broadband ^{1}H decoupling unless mentioned otherwise. Chemical shifts (δ) are given in ppm, relative to deuterated solvent residual peak, and coupling constants (*J*) provided in Hz. ^{19}F NMR shifts are reported relative to the ^{19}F resonances of $CFCl_3$. ^{19}F data at 282 MHz NMR is generally reported with ^{1}H decoupling, and ^{19}F data at 565 MHz NMR is reported without ^{1}H decoupling due to hardware limitations. Mass spectra were acquired using the following instruments: EI - Finnigan MAT 8200 (70 eV); ESI-MS - Bruker ESQ 3000. Accurate mass determinations: Bruker APEX III FT-MS (7 T magnet) or Finnigan MAT 95. UV-Vis absorption spectra were recorded on a Cary 6000i UV-Vis-NiR Spectrophotometer. Chromatographic purifications were performed by flash column chromatography using Merck silica gel 60 (40-63 µm) or by preparative TLC using PLC Silica gel 60 F_{254}, 1 mm, 20x20 cm (Sigma-Aldrich).

Solvents and reagents

Anhydrous MeCN employed in the catalytic reaction was purchased from Sigma-Aldrich, opened and stored in an Ar-filled glovebox. Smaller amounts of MeCN-d_3 and THF-d_8 were purchased and submitted to 3 freeze-pump-thaw cycles, introduced into an Ar-filled glovebox, and then dried/stored under 3 Å molecular sieves. We found this treatment to be enough for carrying out the low-valent bismuth chemistry described herein. Molecular sieves were activated at 200 °C under high vacuum for 3 days. Unless otherwise stated, solvents used for the preparation of the ligands, catalysts or starting materials were also anhydrous, but not degassed nor stored over molecular sieves.

Anhydrous $BiCl_3$ (99.9%, trace metal basis) was purchased from Alfa Aesar and stored in the glovebox. Unless otherwise noted, all reagents were obtained from commercial suppliers and used without further purification.

2. Light sources and photoreaction set-ups

Blue-light irradiation:

Blue LED irradiation (for catalytic experiments) was performed with 100 cm of 24V DC LED strips (centered at 457 nm, 100 cm/140 LEDs, max. output ca. 19 W; max. luminous flow 400 lumen) strapped around a 15-cm glass crystalizing dish. These LED strips were purchased from LEDs24. The reactions were run in 10-mL culture tubes assembled inside the dish surrounded by the LED strip, while the temperature was maintained close to room temperature by using a cooling fan placed on top of the reactor. The reactor was set up on top of a stirring plate. The walls of the reactor were covered with aluminum foil. The photon flux at the blue-light photoreactor employed was determined to be 1.0175×10^{-7} einstein/s by ferrioxalate actinometry (see Section 8.2).

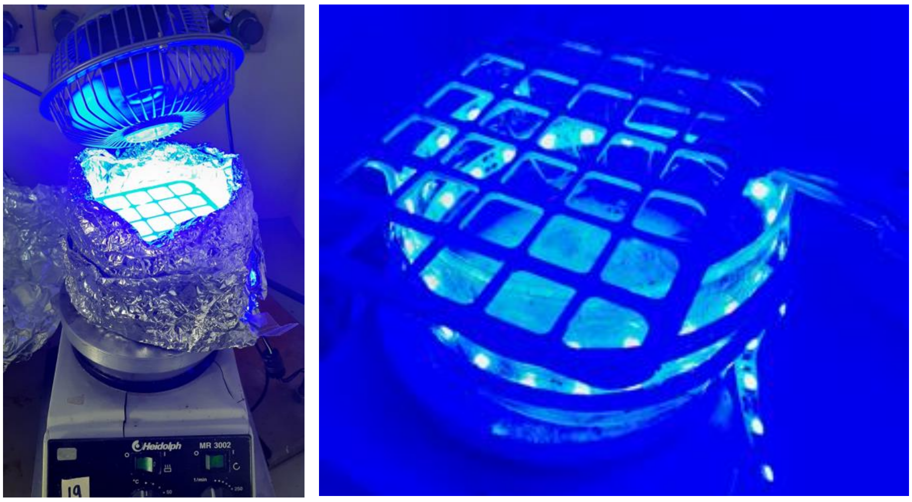

Figure S1. Representative blue-LED strip irradiation set up in a 15 cm glass dish.

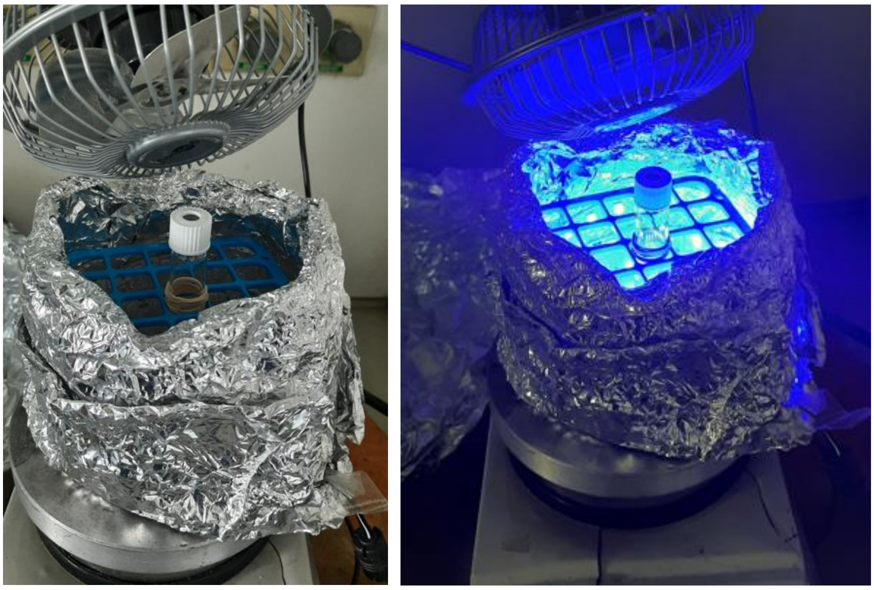

Figure S2. Lights off and on in the blue-LED strip photoreactor used in catalysis.

Red-light irradiation

Red-light irradiation (for stoichiometric oxidative additions) was performed with two 660 nm Kessil lamps at full intensity. Unless mentioned otherwise, the reactions carried out under red-light irradiation were performed using two standardized 660 nm LED PR160L lamps purchased from Kessil.

The inside of the reactor box was fully covered with aluminum foil. The two Kessil lamps were assembled opposite to one another through two holes in the walls of the reactor. As suggested by the manufacturer, the two lamps were assembled at ca. 12 cm from each other. The reaction vessels will be then located at ca. 6 cm from each lamp, receiving an average intensity of 159 mW/cm^2 (see https://kessil.com/products/science_PR160L.php for light-intensity maps and other details). Unless stated otherwise, both PR160L Kessil lamps were used at the 100% intensity setting. The reactor was set up on top of a stirring plate, and the temperature of the reactions was maintained around 35 °C by using a cooling fan placed on top of the reactor.

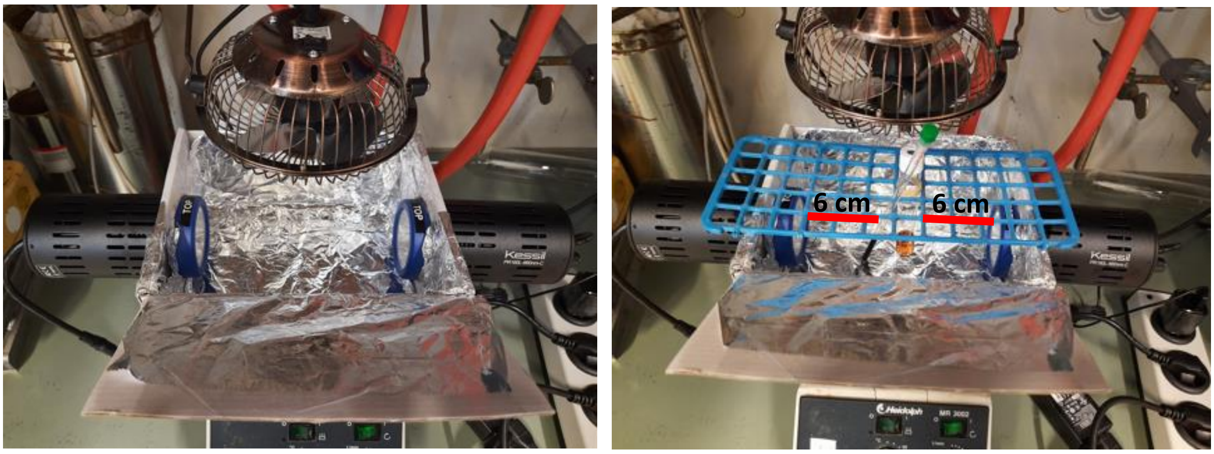

Figure S3. Set up with 2x Kessil 660 nm LED PR160L lamps. The reactions sit at ca. 6 cm from each LED.

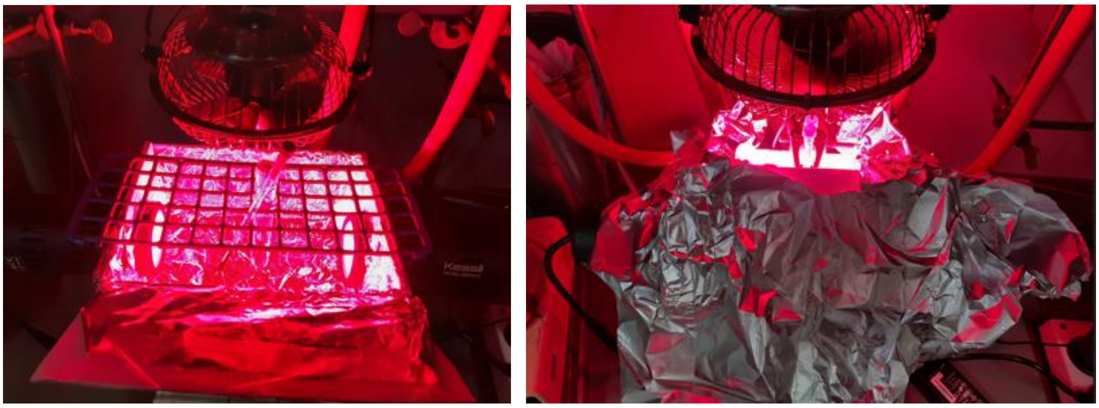

Figure S4. Kessil LED set up with lights on, uncovered (left) or covered with aluminum foil.

Recorded emission spectra of the light sources

The emission spectra of the LEDs employed in this study was recorded using a Cary Eclipse spectrometer, placing the light from the corresponding source close to the fluorimeter emission detector.

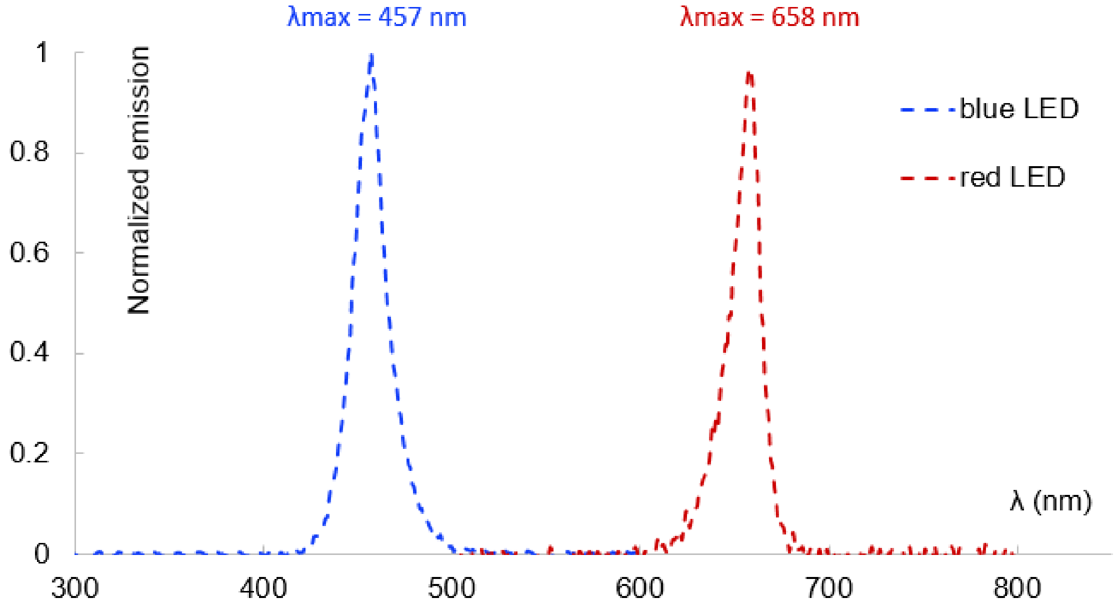

Figure S5. Emission spectra of the two light sources employed in this study: Blue LED strip (blue trace) centered at 457 nm, and red Kessil LED (red trace) centered at 658 nm.

3. Synthesis of bismuthinidenes

The key bismuthinidene used in this study (**1a**) was prepared by scaling up a reported procedure, in two steps from the corresponding aryl bromide **S1**.[1,2]

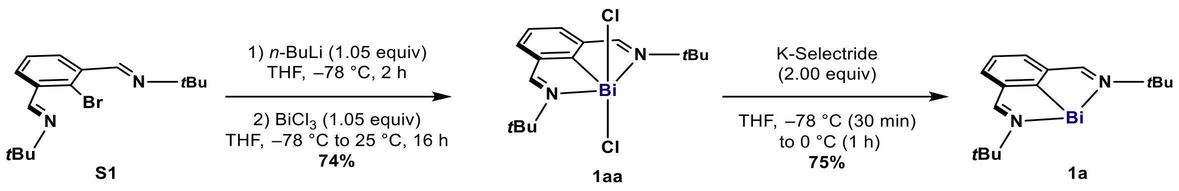

Dichlorobismuthine **1aa**: A heat gun-dried 250 mL Schlenk flask with a magnetic stirring bar was charged with (1*E*,1'*E*)-1,1'-(2-bromo-1,3-phenylene)bis(*N*-tert-butylmethanimine) (**S1**) (3.00 g, 9.28 mmol, 1.00 equiv), and it was dissolved in dry THF (100 mL, ca. 0.1 M). The mixture was cooled down to –78 °C in an acetone/dry ice bath, and to this mixture was added *n*-BuLi dropwise (2.5 M in hexanes, 3.90 mL, 1.05 equiv). The resulting solution was stirred at –78 °C for 2 h. After this time, this solution was transferred in a single portion via cannula to a 250 mL Schlenk flask containing a solution of $BiCl_3$ (3.07 g, 9.74 mmol, 1.05 equiv) in dry THF (60 mL, ca. 0.15 M) under Ar, also pre-cooled to –78 °C. After stirring for 5 min at –78 °C, the cooling bath was removed and the mixture was allowed to stir at room temperature for 16 h. After this time a yellow suspension was obtained, and the rest of the procedure was carried out under air. THF was removed in vacuum, and the obtained residue was re-suspended in 200 mL of HPLC-grade CH_2Cl_2. This was filtered through a plug of Celite®, which was washed with 2 x 100 mL of CH_2Cl_2. The volume of d CH_2Cl_2 was reduced to ca. 75 mL in vacuum, and then, 100 mL of hexanes were added. A precipitate was obtained, which was filtered through a filtering plate, washed twice (2 x 25 mL) with pentane, and dried in high vacuum, to afford **1aa** (3.60 g, 74%) as a white solid, which was carried to the next step without further purification. **^{1}H NMR** (300 MHz, $CDCl_3$) δ 9.61 (s, 2H), 8.17 (d, *J* = 7.5 Hz, 2H), 7.85 (dd, *J* = 7.8, 7.2 Hz, 1H), 1.60 (s, 18H).[1,2]

Bismuthinidene **1a**: A heat gun-dried 250 mL Schlenk flask with a magnetic stirring bar was charged with **1aa** (1.80 g, 3.44 mmol, 1.00 equiv), which was dissolved under argon in anhydrous THF (60 mL, 0.06 M), and cooled down to –78 °C in an acetone/dry ice bath. To this mixture was added dropwise K-Selectride (1.0 M in THF, 6.90 mL, 2.00 equiv) and the resulting dark green mixture was stirred at –78 °C for 30 min and then warmed to 0 °C in an ice/water bath, and stirred for 1 h. Then, THF was removed slowly in vacuum, and the resulting residue was dried in high vacuum for 2 h. The crude was redissolved in anhydrous pentane (ca. 75 mL, in three portions), and filtered through a heat gun-dried glass funnel under argon, into another 250 mL Schlenk flask. The volume of the filtrate was reduced to ca. 20 mL in vacuum, and the solution was cooled to –20 °C overnight. After this time, **1a** was obtained as dark green crystalline needles, from which the mother liquor was removed via cannula before drying in high vacuum. The product (1.15 g, 75%) was stored in an argon-filled glovebox. **^{1}H NMR** (300 MHz, THF-d_8) δ 9.81 (s, 2H), 7.94 (d, *J* = 7.4 Hz, 2H), 7.08 (t, *J* = 7.4 Hz, 1H), 1.57 (s, 18H).[1,2]

Bismuthinidenes **1b** and **1c** evaluated in the optimization process were prepared according to reported procedures.[3,4]

4. Stoichiometric aryl oxidative additions into bismuth(I)

4.1. Red-light promoted oxidative addition of aryl iodides

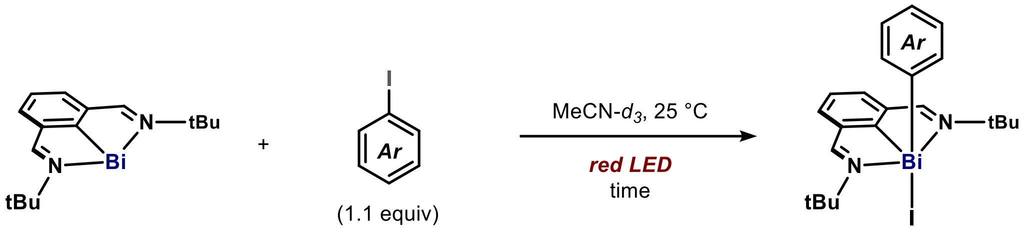

In an argon-filled glovebox, a glass vial with a magnetic stirring bar was charged with bismuthinidene **1a** (1.0 equiv) and the corresponding aryl iodide (1.1 equiv). Both reagents were dissolved in anhydrous, degassed MeCN-d_3 (for characterization) and the solution was transferred into an NMR tube which was closed, taken out of the glovebox and further sealed with parafilm. The resulting homogeneous solution was left red-light irradiation (unless stated otherwise, 2x 660 nm LED PR160L lamps at 100% intensity, purchased from Kessil, see section 2 for details) until full conversion of the starting bismuth(I) complex. Full conversion of bismuth(I) was determined visually, by the disappearance of its dark green color to give light yellow/orange solutions. The yield of the oxidative addition was determined by NMR by repeating the reaction in the presence of 1 equiv of 1,3,5-trimethoxybenzene as internal standard. Unless otherwise stated, full conversion was observed for the starting bismuth(I) complex.

For preparative scale, the reaction was carried out in non-deuterated MeCN, in a 10-mL screw-cap culture tube. The tube was further sealed with parafilm, taken out of the glovebox, and stirred under red-light irradiation (unless stated otherwise, 2x 660 nm LED PR160L lamps at 100% intensity, purchased from Kessil, see section 2 for details). Full conversion of bismuth(I) was determined visually, by the disappearance of its dark green color to give light yellow/orange solutions. Then, the solvent was removed in vacuum, and the product was precipitated by adding diethyl ether and sonication. The resulting powder was filtered through a small frit, and washed 5 times with diethyl ether, to remove most of the unreacted excess aryl iodide. Then, the resulting yellow solid was dried in high vacuum and, if necessary, submitted to crystallization for X-ray analysis.

Characterization data of oxidative addition adducts from aryl iodides

[(2,6-(*t*BuNCH)$_2$C$_6$H$_3$)Bi(4-cyanophenyl)(iodide)] (3a-I)

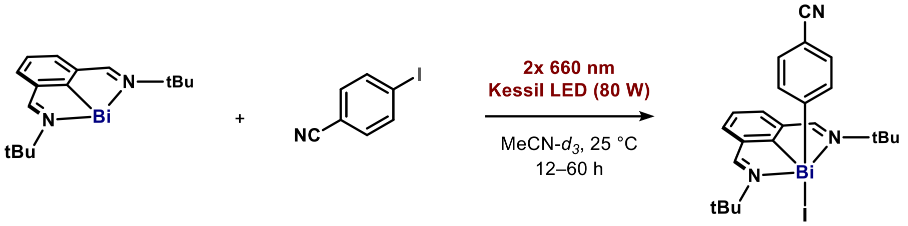

For characterization, the title compound was obtained following the General Procedure above from bismuthinidene **1a** (9.1 mg, 0.020 mmol, 1.0 equiv) and 4-iodobenzonitrile (5.1 mg, 0.022 mmol, 1.1 equiv) in 0.6 mL of MeCN-*d*$_3$ (0.033 M) after 12 h of red-LED irradiation (93% NMR yield). Alternatively, the title compound was also obtained from bismuthinidene **1** (68 mg, 0.15 mmol, 1.0 equiv) and 4-iodobenzonitrile (68 mg, 0.30 mmol, 2.0 equiv) in 2 mL of MeCN (0.08 M) after 60 h of red-LED irradiation and washing with diethyl ether, as a yellow powder (85 mg, 83% yield). Crystals suitable for X-ray diffraction were obtained by vapor diffusion of MTBE into a 1,2-dichloroethane solution.[5]

^{1}H NMR (600 MHz, CD$_3$CN) δ 9.76 (s, 2H), 8.34 (d, *J* = 7.6 Hz, 2H), 8.28 – 8.23 (m, 2H), 8.09 (dd, *J* = 7.8, 7.4 Hz, 1H), 7.81 – 7.75 (m, 2H), 1.30 (s, 18H).

^{13}C NMR (151 MHz, CD$_3$CN) δ 188.98, 185.53, 168.91, 149.51, 139.62, 137.70, 135.81, 131.82, 119.51, 113.44, 62.43, 31.01.

HRMS (ESI Positive): calculated for $C_{23}H_{27}BiN_3$ $[M\text{-}I]^+$: 554.20035; found: 554.20065.

Full assignment for the oxidative addition adduct (see NMR section for details)

NMR data supports the following structure:

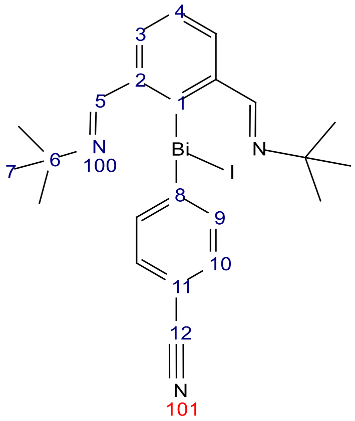

Atom	J	δ (ppm)	HSQC	COSY	HMBC	NOESY
1 C		188.974			3, 5	
2 C		149.511			4, 5	
3 C		137.696	3		3, 5	
H	7.6(4)	8.345	3	4	1, 3, 5	5
4 C		131.824	4			
H	7.6(3)	8.085	4	3	2	
5 C		168.911	5		3	
H		9.762	5		1, 2, 3, 6, 100	3, 7
6 C		62.429			5, 7	
7 C		31.014	7		7	
H3		1.301	7		6, 7, 100	5, 9
8 C		185.530			10	
9 C		139.621	9		9	
H		8.250	9	10	9, 11	7
10 C		135.808	10		10	
H		7.781	10	9	8, 10, 12	
11 C		113.442			9	
12 C		119.506			10	
100 N		-70.800			5, 7	
101 N						

4.1. Oxidative addition of aryl diazonium salts

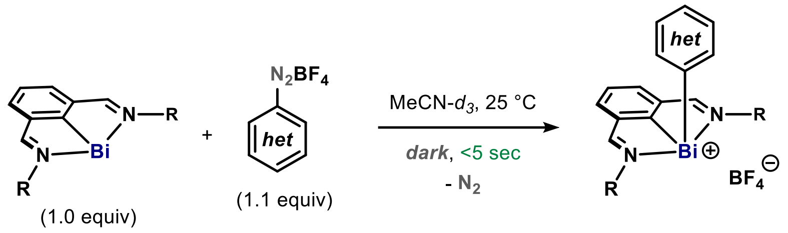

In an argon-filled glovebox, an oven-dried Schlenk flask or a glass vial with a magnetic stirring bar was charged with bismuthinidene **1a** (1.0 equiv), and it was dissolved in anhydrous degassed MeCN-*d*3 (0.1 M). To this stirred solution was added dropwise another solution of the corresponding aryl diazonium salt (1.1 equiv, 0.1 M in MeCN-*d*3). Immediate evolution of nitrogen gas was observed, and color changed from dark green to light yellow/orange upon addition of the full equivalent of electrophile (aryl diazonium salts can also be added as solids in a single portion, but addition of a solution allows for a titration-like control of the addition of 1.0 equiv, due to the significant color change when all bismuth(I) has been consumed). Then, for characterization purposes, the resulting solution was transferred into an NMR tube, which was closed and taken out of the glovebox. Due to the formation of minor side products (mainly from direct reaction of aryl radical with the solvent before recombination with bismuth(II), see section **7** for details), the accurate yield of the oxidative addition was determined by NMR by repeating the reaction in the presence of 1 equiv of 1,3,5-trimethoxybenzene as internal standard. Unless otherwise stated, full conversion was observed for the starting bismuth(I) complex.

[(2,6-(*t*BuNCH)$_2$C$_6$H$_3$)Bi(4-benzonitrile)(tetrafluoroborate)] (**3a-BF$_4$**)

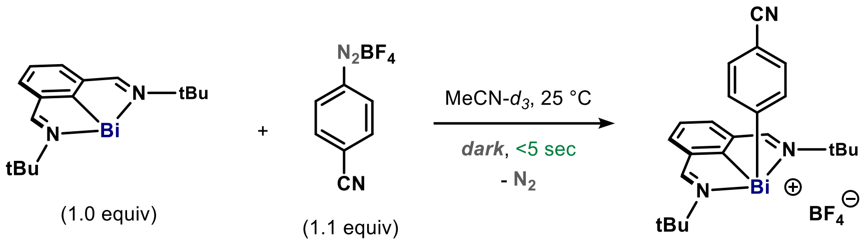

The title compound was obtained following the General Procedure above from bismuthinidene **1** (14 mg, 0.030 mmol, 1.0 equiv) and 4-benzonitrile diazonium tetrafluoroborate (6.7 mg, 0.033 mmol, 1.1 equiv) in 0.6 mL of MeCN-d_3 (0.05 M) immediately after addition (86% NMR yield).

Scale-up procedure: While working in an argon-filled glovebox, a 4 mL scintillation vial equipped with a Teflon-coated magnetic stir bar was charged with bismuthinidene **1a** (45 mg, 0.10 mmol, 1.0 equiv) and anhydrous MeCN (1.0 mL), and the resulting solution stirred at room temperature. In a separate 4 mL vial was prepared a solution of *p*-cyanobenzenediazonium tetrafluoroborate (24 mg, 0.11 mmol, 1.1 equiv) in anhydrous MeCN (0.50 mL). The latter solution was added dropwise to the stirring solution of bismuthinidene until the color of the reaction solution changed from dark green to pale orange. The contents of the vial were stirred for another 5 minutes, after which the vial was ported out of the glovebox. *Note*: the handling of the crude reaction no longer required the use of air-free techniques. The contents of the flask were transferred to a 10 mL round-bottom flask and concentrated to dryness via rotary evaporation. The resulting material was taken up in a minimal amount of anhydrous THF (~ 2 mL) and the desired product was precipitated *via* addition of pentane (20 mL). The product was collected via filtration, washed with MTBE (3 x 5 mL) and pentane (2 x 5 mL), and dried under high vacuum to afford the product as a beige solid (48 mg, 75%). Note: Although ^{1}H NMR analysis shows the presence of < 10% of the (*N,C,N*)Bi^{III}(CH$_2$CN)(BF$_4$) complex, the material was deemed of sufficient quality for the UV-Vis titration experiments in Section 8.1.

Note: for best results, it is highly recommended to use freshly prepared aryldiazonium tetrafluoroborate. Using older samples of aryldiazonium can lead to complex mixtures and low yields of the desired oxidative addition complex.

^{1}H NMR (600 MHz, CD$_3$CN) δ 9.76 (s, 2H), 8.35 (d, *J* = 7.6 Hz, 2H), 8.24 – 8.20 (m, 2H), 8.10 (dd, *J* = 7.8, 7.4 Hz, 1H), 7.79 (d, *J* = 8.3 Hz, 2H), 1.29 (s, 18H).

^{13}C NMR (151 MHz, CD$_3$CN) δ 188.33, 184.49, 168.98, 149.53, 139.51, 137.77, 135.91, 131.98, 119.48, 113.60, 62.45, 30.97.

^{19}F NMR (565 MHz, CD$_3$CN) δ -151.5.

^{11}B NMR (193 MHz, CD$_3$CN) δ -1.17.

HRMS (ESI Positive): calculated for C$_{23}$H$_{27}$BiN$_3$ [M-BF$_4$]$^+$: 554.20035; found: 554.20074.

Preparation of 4-cyanobenzenediazonium tetrafluoroborate

4-Cyanobenzenediazonium tetrafluoroborate was synthesized according to the following adapted literature procedure.[6]

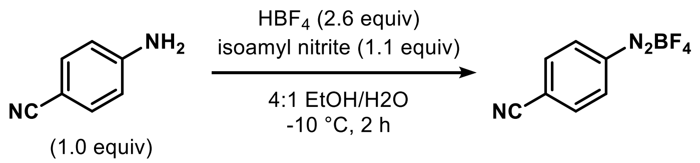

A 2-neck 50 mL round-bottom flask equipped with a Teflon-coated stir bar was charged with 4-aminobenzonitrile (1.063g, 9 mmol, 1 equiv) and a 4:1 EtOH/H_2O solution (10 mL), which were stirred at room temperature to afford a homogeneous solution. Aqueous tetrafluoroboric acid (3.0 mL of a 48 wt% solution in water, 23.4 mmol, 2.6 equiv) was added with a plastic syringe and the resulting solution was cooled down to –10 °C using an acetone/ice bath. To the cooled, stirring solution was added isoamyl nitrite (1.16 g, 9.9 mmol, 1.1 equiv) in a dropwise fashion over a period of ca. 5 minutes. After the addition was finished, the mixture was stirred in the dark for an additional 2 h at -10 °C. The flask was removed from the cold bath and the reaction slurry concentrated to dryness on high vacuum, using the latent heat of vaporization to keep the solution cold. The crude was dissolved in a minimal amount of cold acetone (~15 mL, previously stored in a -20 °C freezer for 2 h) and the purified product was precipitated via the portion-wise addition of cold methyl *tert*-butyl ether (MTBE, 100 mL total, previously stored in a -20 °C freezer for 2 h). The suspension was filtered over a medium fritted funnel plumbed to a water aspirator, and the filtrand was washed with cold MTBE (2 x 25 mL). The remaining solids were dried under high vacuum in the dark to afford the desired product as a white powder that was analytically pure by ^1H NMR (1.753 g, 90%). The latter was stored in a masked vial in the freezer of an argon-filled glovebox (–35 °C).

Note: even when stored in an argon-filled glovebox, the complex slowly decomposes when stored at room temperature in a clear container (solid gradually changes color from white to brown).

5. Reaction development, optimization and control experiments

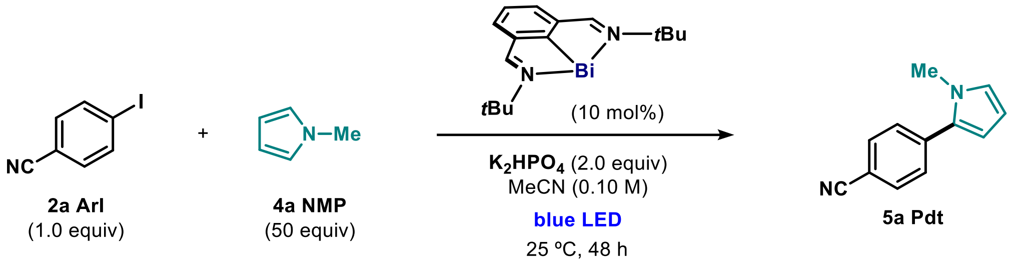

General procedure for optimization and control experiments

While working in an argon-filled glovebox, a flame-dried 10 mL thick-walled culture tube equipped with a Teflon-coated magnetic stir bar was charged with **2a** (1.0 equiv, 0.05 mmol, 11 mg), K_2HPO_4 (2.0 equiv, 0.10 mmol, 18 mg) and bismuth(I) complex **1a** (10 mol%, 2.3 mg). This was followed by the addition of *N*-methylpyrrole (50 equiv, 2.5 mmol, 0.22 mL) and anhydrous MeCN (0.10 M respect to **2a**, 0.50 mL). After this, the tube was closed with a Teflon-lined cap, and the seal further secured with Parafilm. The tube was taken out of the glovebox and subjected to blue-light (457 nm LED strip) with constant stirring at room temperature (the temperature was kept around 25–30 ºC with a cooling fan). After 48 h (unless stated otherwise), the tube was opened and 1.0 equiv of 1,3,5-trimethoxybenzene (8.4 mg) was added as a solution in 0.20 mL of EtOAc. Then, all volatiles were removed in high vacuum, and the resulting residue was dissolved in $CDCl_3$ for crude NMR analysis.

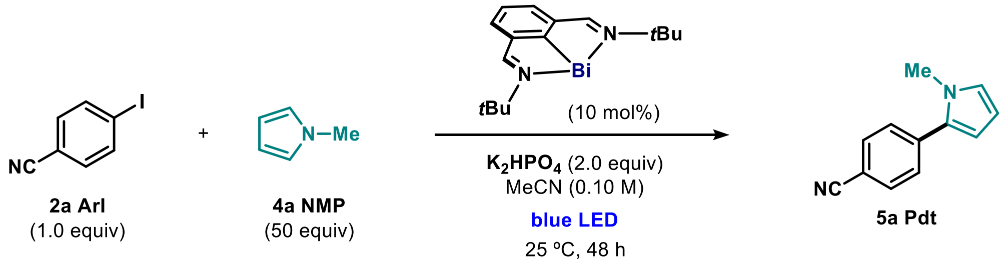

Entry	*Deviations from conditions above*	*Yield Pdt 5a*
**1**	**none**	**58%** (62% ArI Conv)
2	14 h instead of 48 h	38%
3	60 h instead of 48 h	**62%**
4	no light, 45 °C	n/d
5	without base	12%
6	**red LED** instead of **blue LED**	15%
7	blank (no bismuth), 60 h, **blue LED**	<1%
8	blank (no bismuth), 60 h, **red LED**	n/d

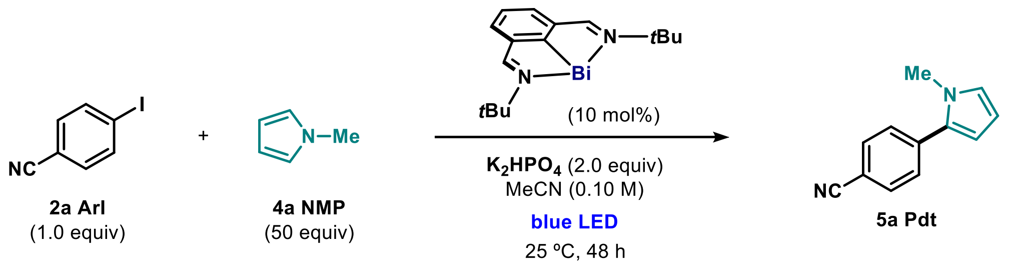

Entry		*Deviations from conditions above*	*Yield Pdt 5a*
9		**CyBisiBi(I) 1b** (10 mol%) instead of **1a**, 60 h	63%
10		**MesBisiBi(I) 1c** (10 mol%) instead of **1a** 14 h	15%
11		5 mol% of **Bi(I) 1a**	31% (45% ArI Conv)
12		20 mol% of **Bi(I) 1a**	49% (81% ArI Conv)
13		10 mol% of **BiI_3** instead of **1a**, 24 h	n/d
14		10 mol% of **$BiCl_3$** instead of **1a**, 24 h	n/d
15		***t*BuBisiBiCl$_2$ 1aa** (10 mol%) instead of **1a**, 24 h	2%
16		***t*BuBisiBiI$_2$ 1ab** (10 mol%) instead of **1a**, 24 h	2%
17		10 equiv of **NMP**	35% (45% ArI Conv)
18		5 equiv of **NMP**	27% (33% ArI Conv)
19		**KH_2PO_4** (pK_a = 2.2) instead of **K_2HPO_4**	19%
20		**$NaHCO_3$** (pK_a = 6.3) instead of **K_2HPO_4**	46%
21	**HPO_4^{2-}** pK_a = 7.2 (58%)	**K_2CO_3** (pK_a = 10.3) instead of **K_2HPO_4**	42%
22		**Na_3PO_4** (pK_a = 12.3) instead of **K_2HPO_4**	59%
23		**NaOH** (pK_a = 14) instead of **K_2HPO_4**	33%
24		THF instead of MeCN	16%
25		DMA instead of MeCN	44%

Note on entries 15–16: The small amount of product (ca. 2% entries 15 and 16) formed in the reactions using $LBiCl_2$ **1aa** or $LBiI_2$ **1ab** might potentially arise from a small amount of LBi(I) generated in-situ via Bi–X homolysis followed by disproportionation of the resulting LBi(II)–X species.

Note on entries 19–23: pK_a values for the conjugated acids of the screened bases are in water and are merely orientative.

6. Bismuth-photocatalyzed activation and coupling of aryl iodides

6.1. General procedure for the coupling reactions

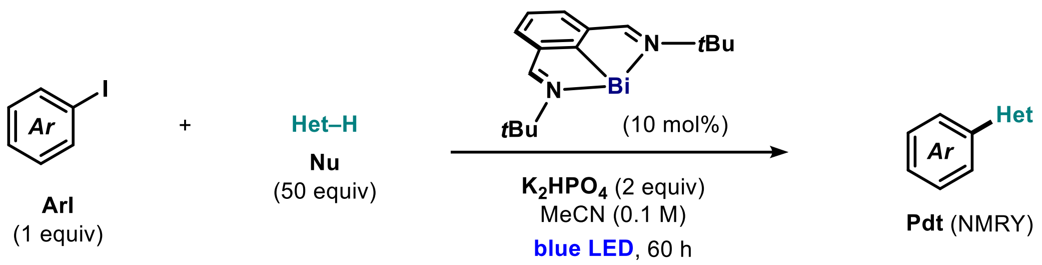

While working in an argon-filled glovebox, a flame-dried 10 mL thick-walled culture tube equipped with a Teflon-coated magnetic stir bar was charged with the corresponding (hetero)aryl iodide **2** (1.0 equiv, 0.2 mmol, unless stated otherwise), K_2HPO_4 (2.0 equiv) and bismuth(I) complex **1a** (10 mol%). This was followed by the addition of the corresponding aromatic nucleophile **4** (50 equiv) and anhydrous MeCN (0.1 M respect to **2**). After this, the tube was closed with a Teflon-lined cap, and the seal further secured with Parafilm. The tube was taken out of the glovebox and subjected to blue-light (457 nm LED strip) with constant stirring at room temperature (the temperature was kept around 25–30 ºC with a cooling fan). Most mixtures stay dark green throughout the course of the reaction, but after >2 days turn into yellow suspensions which suggest the eventual deactivation/decomposition of **1a**. After 60 h (unless stated otherwise) all volatiles were removed in high vacuum. Then, ca. 5 mL of water were added, and the crude mixture was extracted with 3 x 5 mL of EtOAc. The combined organic fractions were concentrated in vacuum, and the resulting crude product was subsequently purified using either flash column chromatography in SiO_2 or preparative TLC in SiO_2, with mixtures of hexanes and EtOAc as eluent.

Note: For completeness, all reactions of the scope were run twice: first at 0.05 mmol-scale for NMR yield determination (following the General Procedure used for the optimization, see Section 5), and then at ca. 0.1-0.2 mmol-scale for product isolation and characterization. Both yields (NMR at 0.05 mmol scale and isolated at 0.2 mmol scale) are reported in the corresponding table of the main text.

6.2. Characterization data for the reaction products

4-(1-Methyl-1*H*-pyrrol-2-yl)benzonitrile (**5a**)

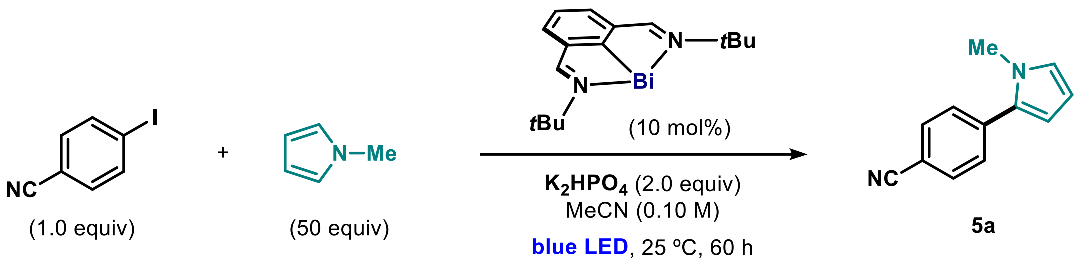

The title product was obtained as a pale yellow residue in 58% yield (21 mg) from 4-iodobenzonitrile (46 mg, 0.20 mmol, 1.0 equiv) and *N*-methylpyrrole (0.90 mL, 10 mmol, 50 equiv) using bismuth complex **1a** as catalyst (9.0 mg, 0.020 mmol, 10 mol%) in MeCN (0.10 M, 2.0 mL) following the General Procedure, after preparative TLC in silica gel (1000 um, 20x20 cm) using 95:5 hexanes/EtOAc (two elutions; R_f = 0.15 in hexanes/EtOAc 95:5).

^{1}H NMR (400 MHz, $CDCl_3$) δ 7.72 – 7.67 (m, 2H), 7.55 – 7.50 (m, 2H), 6.81 (dd, *J* = 2.7, 1.8 Hz, 1H), 6.38 (dd, *J* = 3.7, 1.8 Hz, 1H), 6.26 (dd, *J* = 3.7, 2.7 Hz, 1H), 3.74 (s, 3H).

^{13}C NMR (101 MHz, $CDCl_3$) δ 137.72, 132.65, 132.28, 128.30, 125.87, 119.06, 110.77, 109.72, 108.61, 35.48.

HRMS (EI): calculated for $C_{12}H_{10}N_2$ $[M]^+$: 182.0838; found: 182.0836.

3-(1-Methyl-1*H*-pyrrol-2-yl)benzonitrile (**5b**)

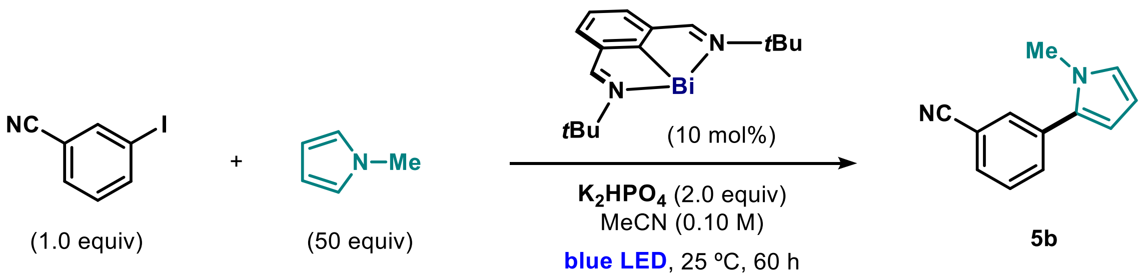

The title product was obtained as a pale yellow residue in 44% yield (16 mg) from 3-iodobenzonitrile (46 mg, 0.20 mmol, 1.0 equiv) and *N*-methylpyrrole (0.90 mL, 10 mmol, 50 equiv) using bismuth complex **1a** as catalyst (9.0 mg, 0.020 mmol, 10 mol%) in MeCN (0.10 M, 2.0 mL) following the General Procedure, after preparative TLC in silica gel (1000 um, 20x20 cm) using 95:5 hexanes/EtOAc (two elutions; R_f = 0.2 in hexanes/EtOAc 95:5).

^{1}H NMR (400 MHz, $CDCl_3$) δ 7.70 (td, *J* = 1.8, 0.6 Hz, 1H), 7.66 (dt, *J* = 7.7, 1.6 Hz, 1H), 7.59 (dt, *J* = 7.7, 1.5 Hz, 1H), 7.52 (td, *J* = 7.7, 0.6 Hz, 1H), 6.79 (dd, *J* = 2.8, 1.8 Hz, 1H), 6.31 (dd, *J* = 3.7, 1.8 Hz, 1H), 6.25 (dd, *J* = 3.7, 2.7 Hz, 1H), 3.71 (s, 3H).

^{13}C NMR (101 MHz, $CDCl_3$) δ 134.60, 132.55, 132.10, 131.59, 129.97, 129.30, 125.05, 118.80, 112.70, 110.06, 108.35, 35.21.

HRMS (EI): calculated for $C_{12}H_{10}N_2$ $[M]^+$: 182.0838; found: 182.0839.

2-(1-Methyl-1*H*-pyrrol-2-yl)benzonitrile (**5c**)

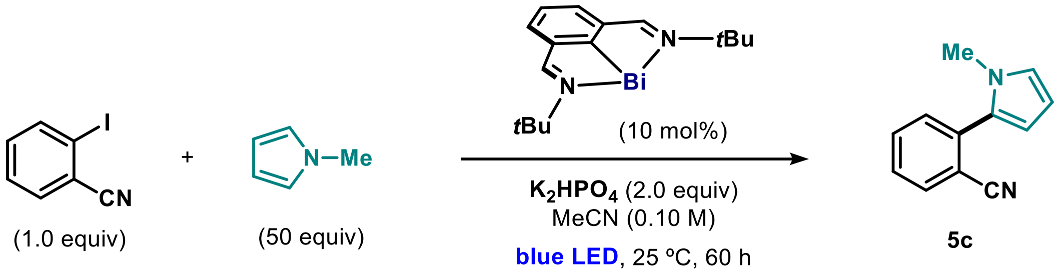

The title product was obtained as a pale yellow amorphous solid in 60% yield (11 mg) from 2-iodobenzonitrile (23 mg, 0.10 mmol, 1.0 equiv) and *N*-methylpyrrole (0.45 mL, 5.0 mmol, 50 equiv) using bismuth complex **1a** as catalyst (4.5 mg, 0.010 mmol, 10 mol%) in MeCN (0.10 M, 1.0 mL) following the General Procedure, after preparative TLC in silica gel (1000 um, 20x20 cm) using 95:5 hexanes/EtOAc (two elutions; R_f = 0.2 in hexanes/EtOAc 95:5).

^{1}H NMR (400 MHz, $CDCl_3$) δ 7.74 (ddd, *J* = 7.8, 1.4, 0.6 Hz, 1H), 7.61 (td, *J* = 7.7, 1.4 Hz, 1H), 7.46 – 7.38 (m, 2H), 6.79 (dd, *J* = 2.7, 1.7 Hz, 1H), 6.41 (dd, *J* = 3.7, 1.8 Hz, 1H), 6.25 (dd, *J* = 3.7, 2.7 Hz, 1H), 3.61 (s, 3H).

^{13}C NMR (101 MHz, $CDCl_3$) δ 136.94, 133.53, 132.34, 130.88, 129.94, 127.41, 124.82, 118.63, 112.88, 111.47, 108.33, 34.83.

HRMS (EI): calculated for $C_{12}H_{10}N_2$ $[M]^+$: 182.0838; found: 182.0839.

4-(1-Methyl-1*H*-pyrrol-2-yl)phenyl trifluoromethanesulfonate (**5d**)

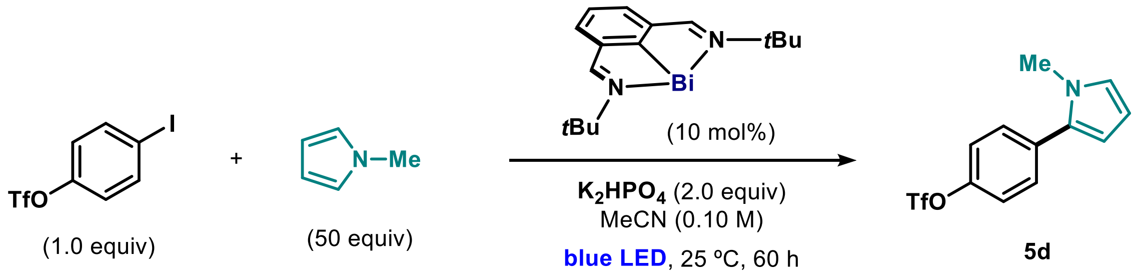

The title product was obtained as a pale yellow residue in 52% yield (16 mg) from 4-iodophenyl trifluoromethanesulfonate (35 mg, 0.10 mmol, 1.0 equiv) and *N*-methylpyrrole (0.45 mL, 5 mmol, 50 equiv) using bismuth complex **1a** as catalyst (4.5 mg, 0.010 mmol, 10 mol%) in MeCN (0.10 M, 1.0 mL) following the General Procedure, after flash column chromatography in silica gel using hexanes/EtOAc 97:3 to 95:5 as eluent (R_f = 0.3 in hexanes/EtOAc 95:5).

^{1}H NMR (600 MHz, $CDCl_3$) δ 7.48 – 7.44 (m, 2H), 7.32 – 7.28 (m, 2H), 6.75 (dd, *J* = 2.7, 1.8 Hz, 1H), 6.26 (dd, *J* = 3.6, 1.8 Hz, 1H), 6.21 (dd, *J* = 3.6, 2.7 Hz, 1H), 3.67 (s, 3H).

^{13}C NMR (151 MHz, $CDCl_3$) δ 148.36, 133.95, 132.67, 130.15, 124.79, 121.50, 118.87 (q, *J* = 320 Hz), 109.88, 108.30, 35.29.

^{19}F NMR (565 MHz, $CDCl_3$) δ -72.83.

HRMS (EI): calculated for $C_{12}H_{10}N_1O_3SF_3$ $[M]^+$: 305.0328; found: 305.0331.

2-(4-Bromophenyl)-1-methyl-1*H*-pyrrole (**5e**)

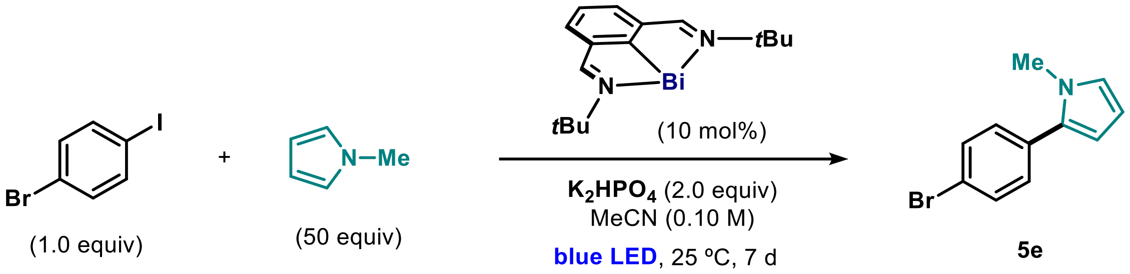

The title product was obtained as a colorless oil in 55% yield (26 mg) from 1-bromo-4-iodobenzene (56 mg, 0.20 mmol, 1.0 equiv) and *N*-methylpyrrole (0.90 mL, 10 mmol, 50 equiv) using bismuth complex **1a** as catalyst (9.0 mg, 0.020 mmol, 10 mol%) in MeCN (0.10 M, 2.0 mL) following the General Procedure (7 days instead of 60 h), after preparative TLC in silica gel (1000 um, 20x20 cm) using 95:5 hexanes/EtOAc (one elution; R_f = 0.4 in hexanes/EtOAc 95:5).

^{1}H NMR (400 MHz, $CDCl_3$) δ 7.57 – 7.52 (m, 2H), 7.32 – 7.28 (m, 2H), 6.75 (dd, *J* = 2.7, 1.9 Hz, 1H), 6.25 (dd, *J* = 3.6, 1.8 Hz, 1H), 6.23 (dd, *J* = 3.6, 2.7 Hz, 1H), 3.68 (s, 3H).

^{13}C NMR (101 MHz, $CDCl_3$) δ 133.36, 132.25, 131.53, 130.09, 124.15, 120.79, 109.03, 108.00, 35.08.

HRMS (EI): calculated for $C_{11}H_{10}N^{79}Br$ $[M]^+$: 234.9991; found: 234.9987.

2-(4-Chlorophenyl)-1-methyl-1*H*-pyrrole (**5f**)

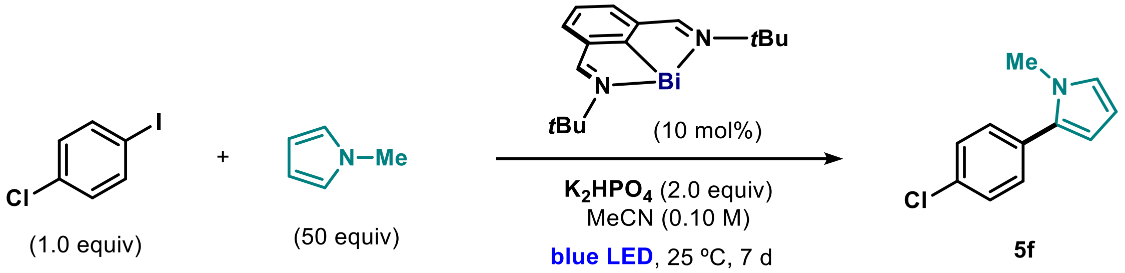

The title product was obtained as a colorless oil in 52% yield (20 mg) from 1-chloro-4-iodobenzene (48 mg, 0.20 mmol, 1.0 equiv) and *N*-methylpyrrole (0.90 mL, 10 mmol, 50 equiv) using bismuth complex **1a** as catalyst (9.0 mg, 0.020 mmol, 10 mol%) in MeCN (0.10 M, 2.0 mL) following the General Procedure, after preparative TLC in silica gel (1000 um, 20x20 cm) using 95:5 hexanes/EtOAc (one elution; R_f = 0.4 in hexanes/EtOAc 95:5).

^{1}H NMR (400 MHz, $CDCl_3$) δ 7.39 – 7.31 (m, 4H), 6.72 (dd, *J* = 2.7, 1.9 Hz, 1H), 6.24 – 6.19 (m, 2H), 3.65 (s, 3H).

^{13}C NMR (101 MHz, $CDCl_3$) δ 133.36, 132.69, 131.80, 129.78, 128.58, 124.06, 109.00, 107.95, 35.07.

HRMS (EI): calculated for $C_{11}H_{10}NCl$ $[M]^+$: 191.0496; found: 191.0496.

2-(3-bromo-5-(trifluoromethyl)phenyl)-1-methyl-1*H*-pyrrole (5g)

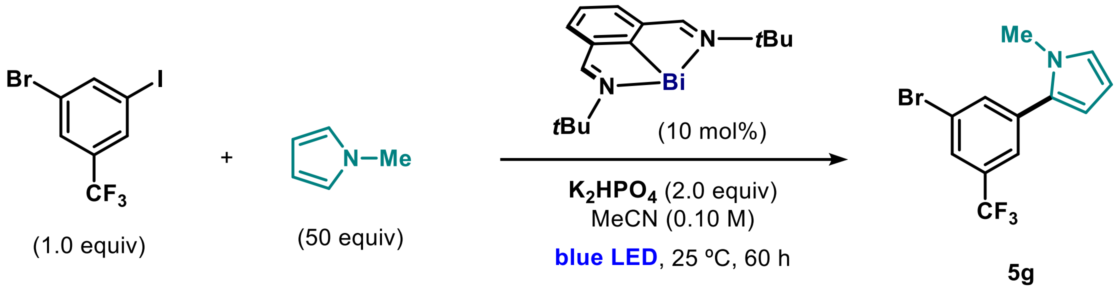

5g

The title product was obtained as a colorless amorphous solid in 79% yield (43 mg) from 1-bromo-3-iodo-5-(trifluoromethyl)benzene (70 mg, 0.20 mmol, 1.0 equiv) and *N*-methylpyrrole (0.90 mL, 10 mmol, 50 equiv) using bismuth complex **1a** as catalyst (9.0 mg, 0.020 mmol, 10 mol%) in MeCN (0.10 M, 2.0 mL) following the General Procedure, after preparative TLC in silica gel (1000 um, 20x20 cm) using 95:5 hexanes/EtOAc (one elution; R_f = 0.5 in hexanes/EtOAc 95:5).

^{1}H NMR (400 MHz, $CDCl_3$) δ 7.75 (d, *J* = 1.8 Hz, 1H), 7.70 (dt, *J* = 1.8, 0.9 Hz, 1H), 7.60 (tt, *J* = 1.5, 0.7 Hz, 1H), 6.79 (dd, *J* = 2.7, 1.8 Hz, 1H), 6.34 (dd, *J* = 3.7, 1.8 Hz, 1H), 6.24 (dd, *J* = 3.7, 2.7 Hz, 1H), 3.72 (s, 3H).

^{13}C NMR (101 MHz, $CDCl_3$) δ 136.02, 134.21, 132.43 (q, *J* = 33.0 Hz), 126.20 (q, *J* = 3.8 Hz), 125.34, 124.02 (q, *J* = 266.0 Hz), 123.62 (q, *J* = 3.9 Hz), 122.70, 110.48, 108.45, 35.20.

^{19}F NMR (282 MHz, $CDCl_3$) δ -62.87.

HRMS (EI): calculated for $C_{12}H_9NF_3{}^{79}Br$ $[M]^+$: 302.9865; found: 302.9868.

4-(1-Methyl-1*H*-pyrrol-2-yl)-2-(trifluoromethyl)pyridine (**5h**)

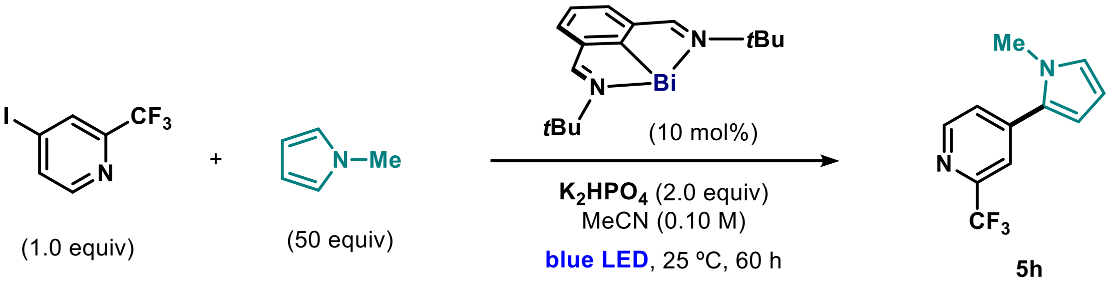

5h

The title product was obtained as a pale yellow residue in 77% yield (35 mg) from 4-iodo-2-(trifluoromethyl)pyridine (55 mg, 0.20 mmol, 1.0 equiv) and *N*-methylpyrrole (0.90 mL, 10 mmol, 50 equiv) using bismuth complex **1a** as catalyst (9.0 mg, 0.020 mmol, 10 mol%) in MeCN (0.10 M, 2.0 mL) following the General Procedure, after preparative TLC in silica gel (1000 um, 20x20 cm) using 9:1 hexanes/EtOAc (two elutions; R_f = 0.2 in hexanes/EtOAc 9:1).

^{1}H NMR (600 MHz, $CDCl_3$) δ 8.68 (dt, *J* = 5.1, 0.7 Hz, 1H), 7.70 (dd, *J* = 1.7, 0.8 Hz, 1H), 7.48 (ddd, *J* = 5.2, 1.8, 0.6 Hz, 1H), 6.83 (dd, *J* = 2.7, 1.8 Hz, 1H), 6.52 (dd, *J* = 3.8, 1.8 Hz, 1H), 6.26 (dd, *J* = 3.8, 2.7 Hz, 1H), 3.79 (s, 3H).

^{13}C NMR (151 MHz, $CDCl_3$) δ 150.25, 148.69 (q, *J* = 34.3 Hz), 142.24, 130.48, 127.57, 123.94, 121.71 (q, *J* = 273 Hz), 118.51 (q, *J* = 2.7 Hz), 112.44, 109.21, 35.91.

^{19}F NMR (565 MHz, $CDCl_3$) δ -68.18.

HRMS (EI): calculated for $C_{12}H_{10}N_2$ $[M]^+$: 226.0712; found: 226.0714.

2-(1-Methyl-1*H*-pyrrol-2-yl)pyrazine (5i)

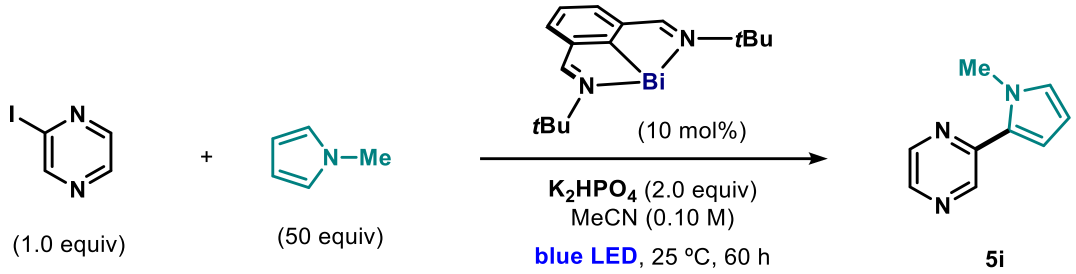

The title product was obtained as a pale yellow amorphous solid in 82% yield (26 mg) from 2-iodopyrazine (41 mg, 0.20 mmol, 1.0 equiv) and *N*-methylpyrrole (0.90 mL, 10 mmol, 50 equiv) using bismuth complex **1a** as catalyst (9.0 mg, 0.020 mmol, 10 mol%) in MeCN (0.10 M, 2.0 mL) following the General Procedure, after preparative TLC in silica gel (1000 um, 20x20 cm) using 9:1 hexanes/EtOAc (two elutions; R_f = 0.2 in hexanes/EtOAc 9:1).

^{1}H NMR (400 MHz, $CDCl_3$) δ 8.86 (d, *J* = 1.6 Hz, 1H), 8.48 (dd, *J* = 2.6, 1.6 Hz, 1H), 8.31 (d, *J* = 2.6 Hz, 1H), 6.84 – 6.80 (m, 1H), 6.75 (dd, *J* = 3.9, 1.7 Hz, 1H), 6.24 (dd, *J* = 3.9, 2.6 Hz, 1H), 4.03 (s, 3H).

^{13}C NMR (101 MHz, $CDCl_3$) δ 148.59, 142.98, 142.80, 140.21, 128.91, 127.95, 111.91, 108.27, 37.22.

HRMS (EI): calculated for $C_9H_9N_3$ $[M]^+$: 159.0790; found: 159.0791.

2-(1-Methyl-1*H*-pyrrol-2-yl)pyridine (5j)

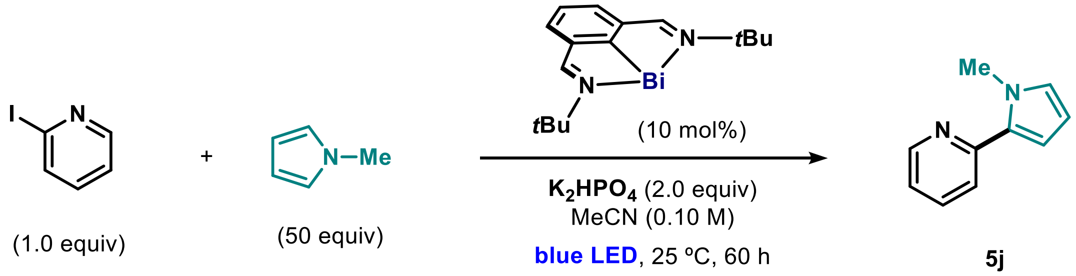

The title product was obtained as a pale yellow residue in 41% yield (13 mg) from 2-iodopyridine (41 mg, 0.20 mmol, 1.0 equiv) and *N*-methylpyrrole (0.90 mL, 10 mmol, 50 equiv) using bismuth complex **1a** as catalyst (9.0 mg, 0.020 mmol, 10 mol%) in MeCN (0.10 M, 2.0 mL) following the General Procedure, after preparative TLC in silica gel (1000 um, 20x20 cm) using 95:5 hexanes/EtOAc (two elutions; R_f = 0.25 in hexanes/EtOAc 95:5).

^{1}H NMR (400 MHz, $CDCl_3$) δ 8.58 (ddd, *J* = 4.9, 1.9, 1.0 Hz, 1H), 7.66 (ddd, *J* = 8.0, 7.4, 1.9 Hz, 1H), 7.55 (dt, *J* = 8.1, 1.1 Hz, 1H), 7.09 (ddd, *J* = 7.4, 4.9, 1.2 Hz, 1H), 6.76 (dd, *J* = 2.6, 1.8 Hz, 1H), 6.59 (dd, *J* = 3.8, 1.8 Hz, 1H), 6.20 (dd, *J* = 3.7, 2.6 Hz, 1H), 4.02 (s, 3H).

^{13}C NMR (101 MHz, $CDCl_3$) δ 152.76, 148.59, 136.20, 132.37, 126.35, 121.49, 120.23, 110.69, 107.64, 36.88.

HRMS (EI): calculated for $C_{10}H_{10}N_2$ $[M]^+$: 158.0838; found: 158.0837.

5-Bromo-2-(1-methyl-1*H*-pyrrol-2-yl)pyridine (**5k**)

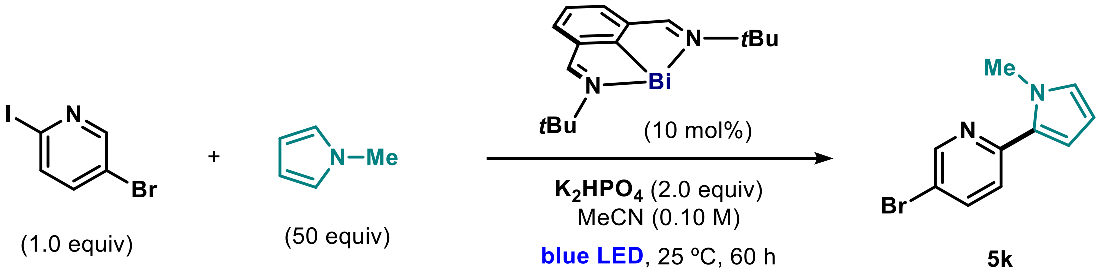

The title product was obtained as a colorless viscous oil in 80% yield (42 mg, containing 9% of starting heteroaryl iodide, yield corrected by NMR) from 5-bromo-2-iodopyridine (57 mg, 0.20 mmol, 1.0 equiv) and *N*-methylpyrrole (0.90 mL, 10 mmol, 50 equiv) using bismuth complex **1a** as catalyst (9.0 mg, 0.020 mmol, 10 mol%) in MeCN (0.10 M, 2.0 mL) following the General Procedure, after flash column chromatography in silica gel using hexanes/EtOAc 97:3 to 95:5 as eluent (R_f = 0.5 in hexanes/EtOAc 95:5).

^{1}H NMR (400 MHz, $CDCl_3$) δ 8.62 (dd, *J* = 2.3, 0.8 Hz, 1H), 7.76 (dd, *J* = 8.6, 2.4 Hz, 1H), 7.62 – 7.42 (m, 1H), 6.77 (dd, *J* = 2.6, 1.8 Hz, 1H), 6.61 (dd, *J* = 3.8, 1.8 Hz, 1H), 6.20 (dd, *J* = 3.8, 2.6 Hz, 1H), 4.00 (s, 3H).

^{13}C NMR (101 MHz, $CDCl_3$) δ 151.11, 149.46, 138.81, 131.12, 127.02, 122.44, 116.89, 111.24, 107.88, 37.16.

HRMS (EI): calculated for $C_{10}H_9N_2{}^{79}Br$ $[M]^+$: 235.9943; found: 235.9944.

2-Bromo-5-(1-methyl-1*H*-pyrrol-2-yl)pyridine (**5l**)

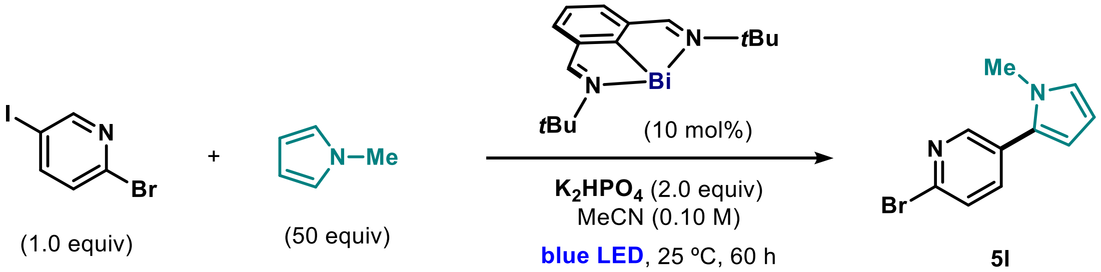

The title product was obtained as a white solid in 49% yield (23 mg) from 2-bromo-5-iodopyridine (57 mg, 0.20 mmol, 1.0 equiv) and *N*-methylpyrrole (0.90 mL, 10 mmol, 50 equiv) using bismuth complex **1a** as catalyst (9.0 mg, 0.020 mmol, 10 mol%) in MeCN (0.10 M, 2.0 mL) following the General Procedure, after preparative TLC in silica gel (1000 um, 20x20 cm) using 9:1 hexanes/EtOAc (one elution; R_f = 0.5 in hexanes/EtOAc 9:1).

^{1}H NMR (400 MHz, $CDCl_3$) δ 8.45 (dd, *J* = 2.5, 0.8 Hz, 1H), 7.59 (dd, *J* = 8.2, 2.5 Hz, 1H), 7.53 (dd, *J* = 8.2, 0.8 Hz, 1H), 6.80 (dd, *J* = 2.7, 1.8 Hz, 1H), 6.32 (dd, *J* = 3.7, 1.8 Hz, 1H), 6.25 (dd, *J* = 3.6, 2.7 Hz, 1H), 3.69 (s, 3H).

^{13}C NMR (101 MHz, $CDCl_3$) δ 149.24, 139.96, 137.89, 129.46, 128.57, 127.73, 125.28, 110.29, 108.52, 35.14.

HRMS (EI): calculated for $C_{10}H_9N_2{}^{79}Br$ $[M]^+$: 235.9943; found: 235.9943.

7-Chloro-4-(1-methyl-1*H*-pyrrol-2-yl)quinoline (**5m**)

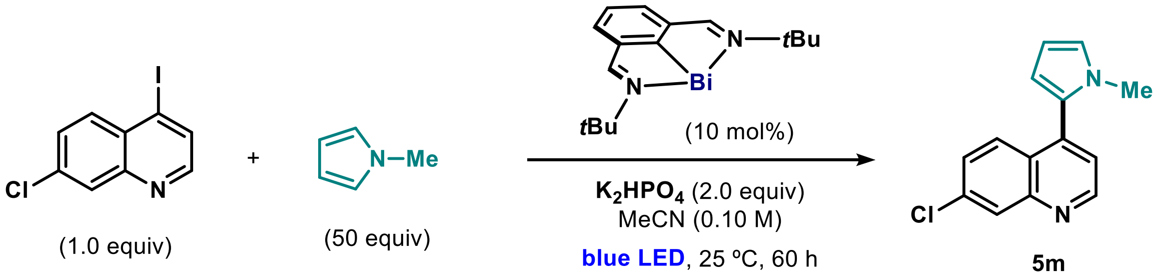

5m

The title product was obtained as a pale yellow amorphous solid in 41% yield (20 mg) from 7-chloro-4-iodoquinoline (58 mg, 0.20 mmol, 1.0 equiv) and *N*-methylpyrrole (0.90 mL, 10 mmol, 50 equiv) using bismuth complex **1a** as catalyst (9.0 mg, 0.020 mmol, 10 mol%) in MeCN (0.10 M, 2.0 mL) following the General Procedure, after preparative TLC in silica gel (1000 um, 20x20 cm) using 8:2 hexanes/EtOAc (two elutions; R_f = 0.2 in hexanes/EtOAc 9:1).

^{1}H NMR (400 MHz, $CDCl_3$) δ 8.95 (d, *J* = 4.5 Hz, 1H), 8.17 (d, *J* = 2.2 Hz, 1H), 7.90 (d, *J* = 9.0 Hz, 1H), 7.50 (dd, *J* = 9.0, 2.2 Hz, 1H), 7.33 (d, *J* = 4.5 Hz, 1H), 6.90 (dd, *J* = 2.7, 1.8 Hz, 1H), 6.39 (dd, *J* = 3.6, 1.8 Hz, 1H), 6.36 (dd, *J* = 3.6, 2.7 Hz, 1H), 3.53 (s, 3H).

^{13}C NMR (101 MHz, $CDCl_3$) δ 150.84, 149.25, 139.64, 135.39, 128.91, 128.70, 127.86, 127.76, 126.24, 124.83, 122.28, 112.22, 108.50, 34.90.

HRMS (EI): calculated for $C_{14}H_{11}N_2Cl$ $[M]^+$: 242.0605; found: 242.0607.

2-(1-Methyl-1*H*-pyrrol-2-yl)quinoxaline (**5n**)

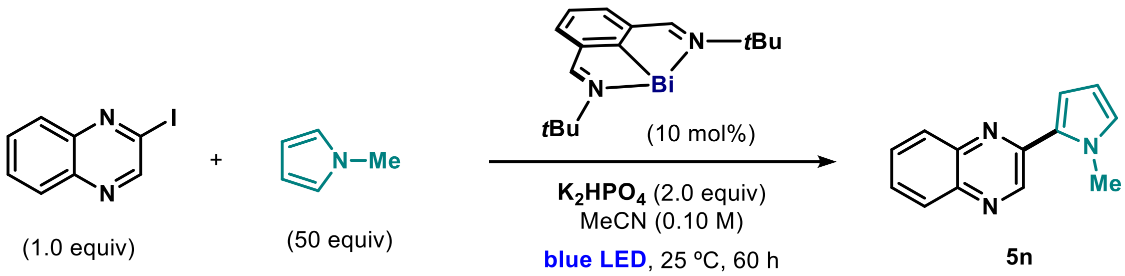

5n

The title product was obtained as a yellow solid in 96% yield (40 mg) from 2-iodoquinoxaline (51 mg, 0.20 mmol, 1.0 equiv) and *N*-methylpyrrole (0.90 mL, 10 mmol, 50 equiv) using bismuth complex **1a** as catalyst (9.0 mg, 0.020 mmol, 10 mol%) in MeCN (0.10 M, 2.0 mL) following the General Procedure, after preparative TLC in silica gel (1000 um, 20x20 cm) using 9:1 hexanes/EtOAc (one elution; R_f = 0.6 in hexanes/EtOAc 9:1).

^{1}H NMR (400 MHz, $CDCl_3$) δ 9.16 (s, 1H), 8.07 – 8.02 (m, 1H), 8.00 (ddd, *J* = 8.2, 1.5, 0.6 Hz, 1H), 7.72 (ddd, *J* = 8.4, 6.9, 1.6 Hz, 1H), 7.66 (ddd, *J* = 8.3, 6.9, 1.5 Hz, 1H), 6.98 (dd, *J* = 3.9, 1.7 Hz, 1H), 6.91 – 6.86 (m, 1H), 6.30 (dd, *J* = 3.9, 2.6 Hz, 1H), 4.22 (s, 3H).

^{13}C NMR (101 MHz, $CDCl_3$) δ 147.15, 144.57, 141.58, 139.82, 129.94, 129.41, 129.06, 129.03, 128.88, 128.27, 113.56, 108.51, 37.89.

HRMS (EI): calculated for $C_{13}H_{11}N_3$ $[M]^+$: 209.0947; found: 209.0946.

4-(3,5-Dimethyl-1*H*-pyrrol-2-yl)benzonitrile (**5o**)

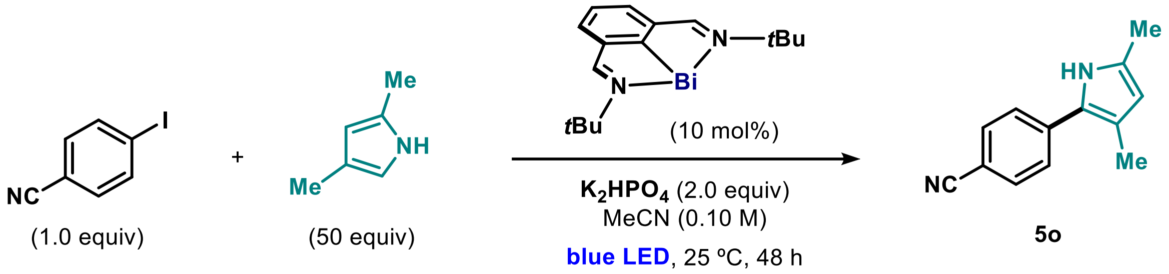

The title product was obtained as a white amorphous solid in 45% yield (15 mg) from 4-iodobenzonitrile (46 mg, 0.20 mmol, 1.0 equiv) and 2,4-dimethyl-1*H*-pyrrole (0.70 mL, 10 mmol, 50 equiv) using bismuth complex **1a** as catalyst (9.0 mg, 0.020 mmol, 10 mol%) in MeCN (0.10 M, 2.0 mL) following the General Procedure, after preparative TLC in silica gel (1000 um, 20x20 cm) using 8:2 hexanes/EtOAc (two elutions; R_f = 0.6 in hexanes/EtOAc 7:3).

^{1}H NMR (400 MHz, $CDCl_3$) δ 7.98 (s, 1H), 7.67 – 7.62 (m, 2H), 7.50 – 7.44 (m, 2H), 5.90 (d, *J* = 2.8 Hz, 1H), 2.33 (d, *J* = 0.9 Hz, 3H), 2.29 (s, 3H).

^{13}C NMR (101 MHz, $CDCl_3$) δ 137.98, 132.56, 129.92, 125.12, 124.86, 120.00, 119.41, 111.67, 107.72, 13.13, 13.06.

HRMS (EI): calculated for $C_{13}H_{12}N_2$ $[M]^+$: 196.0995; found: 196.0994.

4-(1*H*-Pyrrol-2-yl)benzonitrile (**5p**)

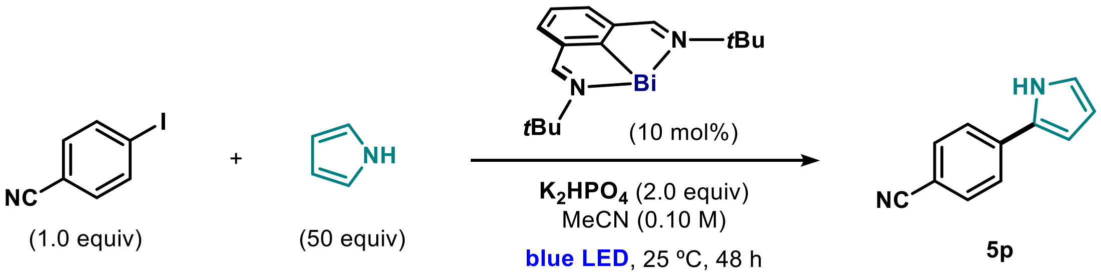

The title product was obtained as a white amorphous solid in 45% yield (15 mg) from 4-iodobenzonitrile (46 mg, 0.20 mmol, 1.0 equiv) and 1*H*-pyrrole (0.70 mL, 10 mmol, 50 equiv) using bismuth complex **1a** as catalyst (9.0 mg, 0.020 mmol, 10 mol%) in MeCN (0.10 M, 2.0 mL) following the General Procedure, after preparative TLC in silica gel (1000 um, 20x20 cm) using 8:2 hexanes/EtOAc (two elutions; R_f = 0.6 in hexanes/EtOAc 7:3).

^{1}H NMR (400 MHz, $CDCl_3$) δ 8.62 (s, 1H), 7.67 – 7.62 (m, 2H), 7.59 – 7.53 (m, 2H), 6.98 (td, *J* = 2.8, 1.4 Hz, 1H), 6.70 (ddd, *J* = 3.9, 2.7, 1.4 Hz, 1H), 6.37 (dt, *J* = 3.7, 2.6 Hz, 1H).

^{13}C NMR (101 MHz, $CDCl_3$) δ 136.75, 132.82, 130.12, 123.68, 120.99, 119.19, 111.02, 108.82, 108.78.

HRMS (EI): calculated for $C_{11}H_8N_2$ $[M]^+$: 168.0682; found: 168.0683.

6.3. Unsuccessful or low-yielding substrates

As expected, the reaction rate of the catalytic activation of aryl iodides decreases as electron density increases. This correlates perfectly with the rate previously observed for the oxidative addition process, further solidifying the idea that this is the rate-limiting step of the catalytic reaction.

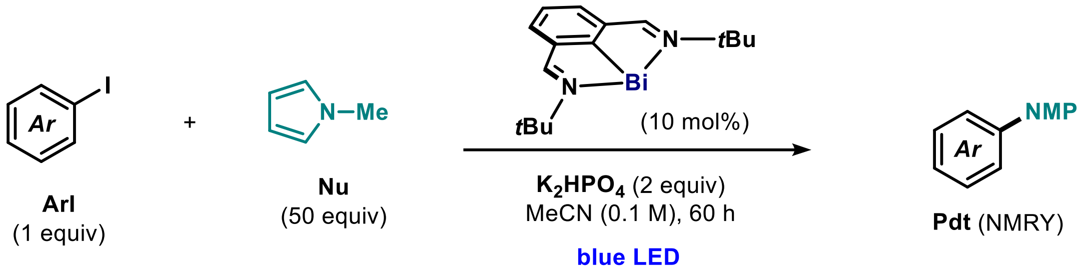

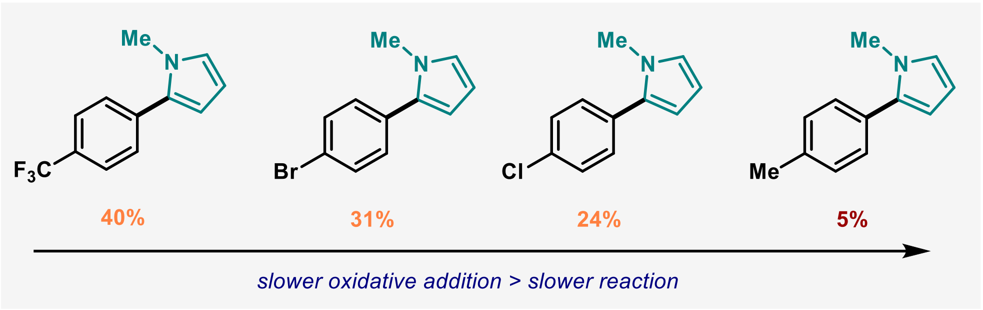

Some of the few electron-poor (hetero)aryl iodides that we found to be less efficient are listed below:

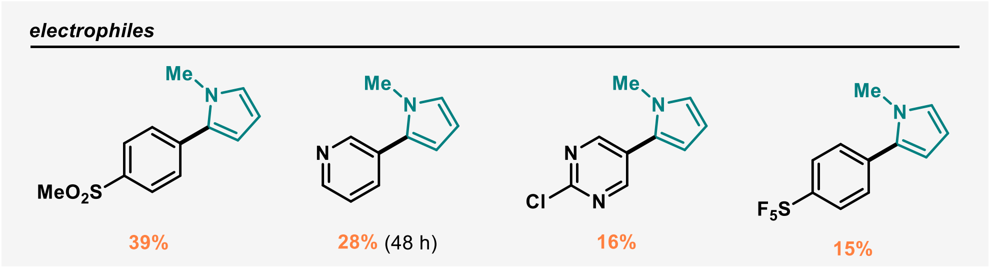

All yields determined by ^{1}H NMR using 1,3,5-trimethoxybenzene as internal standard.

Evaluation of higher intensity blue light

Since a higher photon flux is expected to increase the rates of both oxidative addition and Bi-C bond homolysis, the effect of the light intensity on the yield of the catalytic reactions was investigated. Several entries in the catalytic reaction were repeated in the presence of a higher intensity blue light source (2 x 40 W 456 nm PR160L Kessil lamps). The yields of the desired product were higher in all cases with the higher intensity light source.

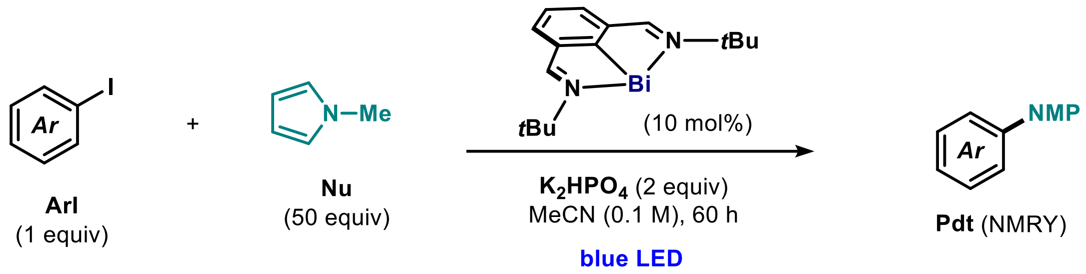

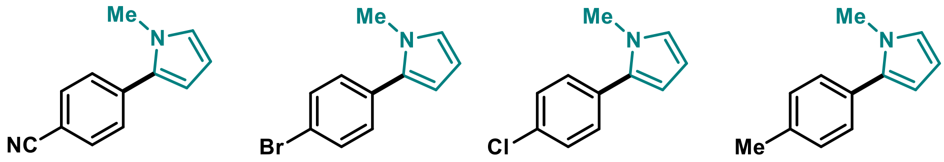

**2 x 40 W Kessil lamps**	**58%** (24 h)	**43%** (48 h)	**43%** (48 h)	**20%** (48 h)
**LED strip (ca. 20 W)**	**58%** (48 h)	**31%** (60 h)	**24%** (60 h)	**5%** (60 h)

All yields determined by ^{1}H NMR using 1,3,5-trimethoxybenzene as internal standard.

Thus, these preliminary results show that simply by increasing the light intensity, we can increase the overall rate of the reaction. Shortening the reaction time allows the activation of less electron-deficient aryl iodides in shorter timeframes.

Evaluation of other nucleophiles

As a proof-of-concept for generality, other nucleophiles were tested, under identical conditions and with no optimization whatsoever. The yield of the products was determined by ^{1}H NMR

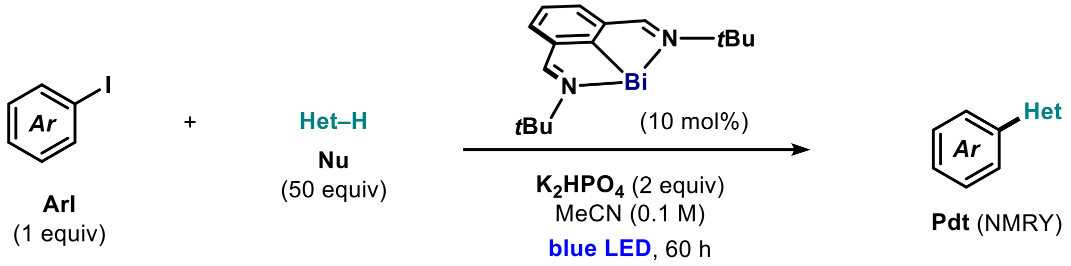

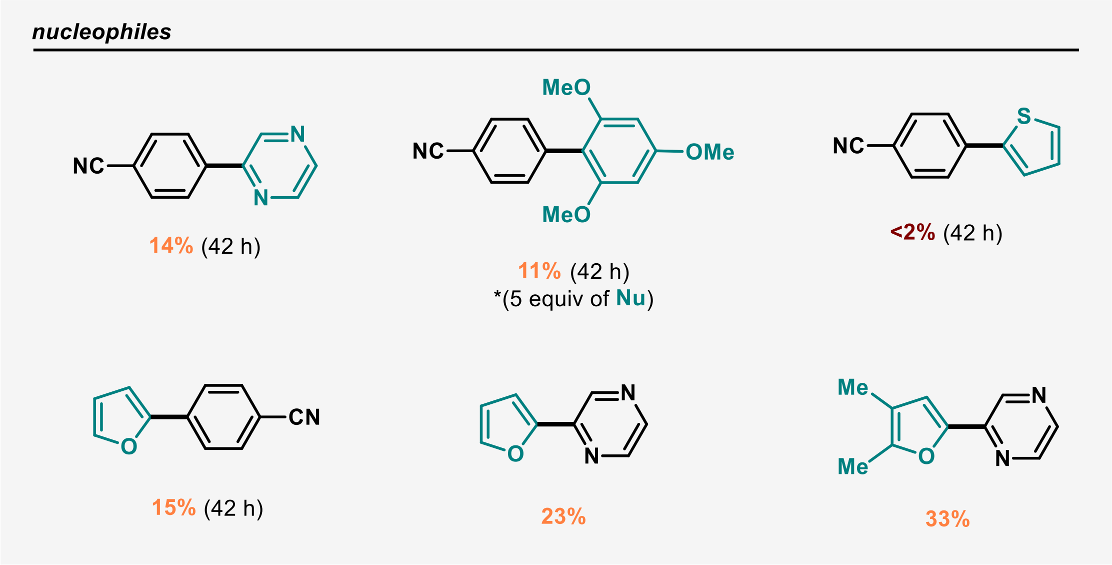

All yields determined by ^{1}H NMR using 1,3,5-trimethoxybenzene as internal standard.

6.4. Miscellaneous information

Illustrative GC-MS of a sample reaction

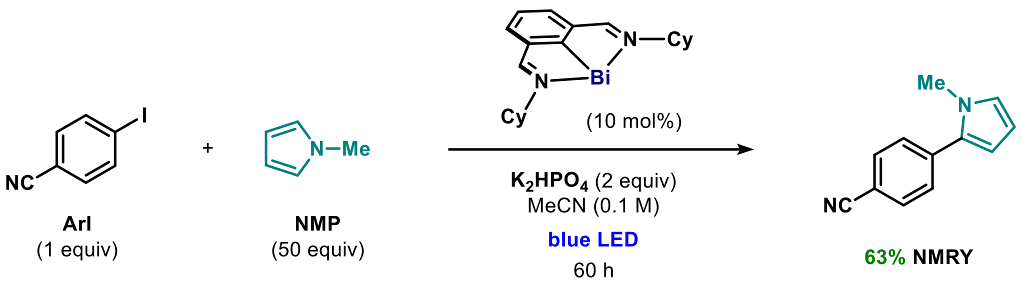

The cleanness of these reactions is illustrated by the GC-MS shown below, after addition of internal standard and quick filtration through a silica-gel pipette.

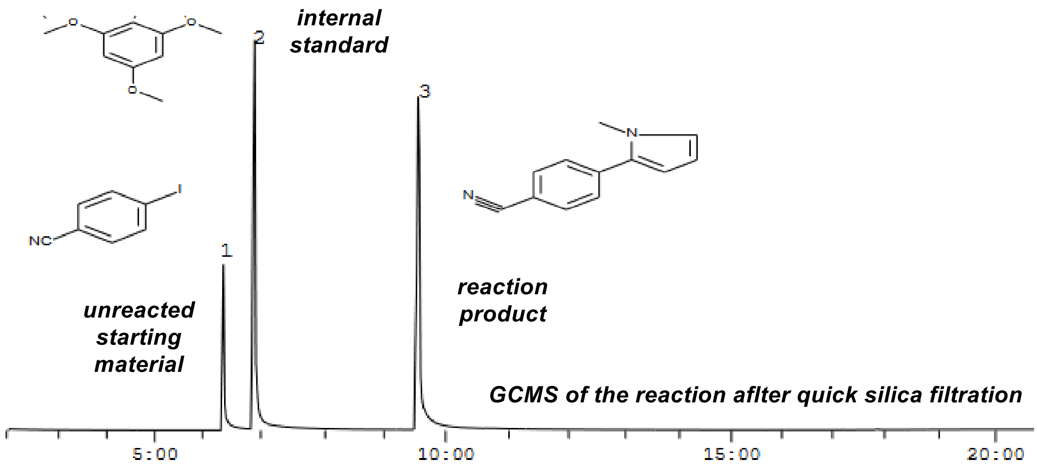

NMR analysis and product distribution of a finished reaction

Crude NMR spectrum of a finished reaction after removal of MeCN and other volatiles and redissolving in $CDCl_3$ (containing 1 equiv of 1,3,5-trimethoxybenzene as internal standard) under air.

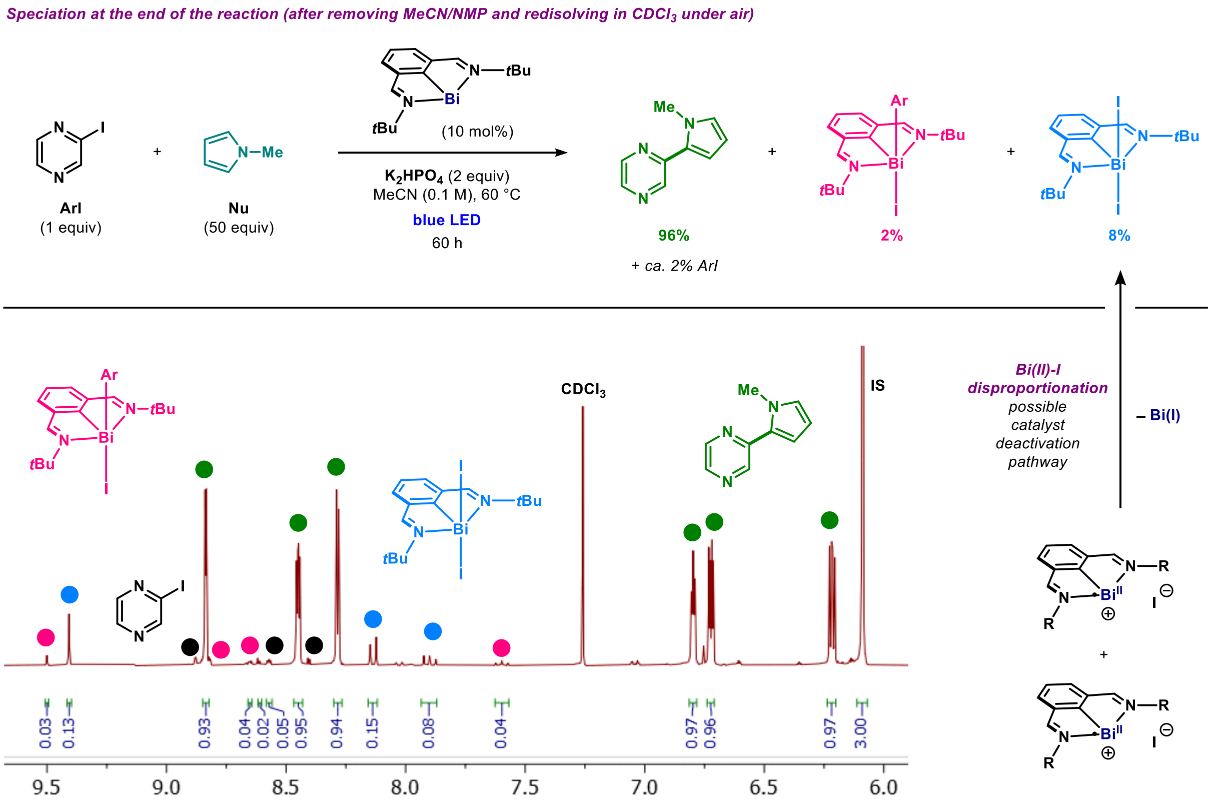

While (L)Bi(III)I_2 could arise during the reaction upon unproductive disproportionation of (L)Bi(II)–I radical intermediates, preliminary monitoring experiments under inert conditions do not reveal formation of this complex. This suggests that formation of (L)Bi(III)I_2 **1ab** could be occurring *after* workup under air, via the oxidation of remaining Bi(I) with oxygen in the presence of excess iodide salts generated during the reaction.

Note: the scarce solubility of (L)Bi(III)I_2 **1ab** in MeCN makes in-situ quantification more difficult. The solubility of this compound in $CDCl_3$ is much higher, allowing a recovery/quantification of most of it (e.g.: in the reaction above, ca. 8 mol% of **1ab** out of 10 mol% catalyst loading).

Identification and preparation of (L)Bi(III)I_2 (1ab)

In order to unequivocally identify the main (L)Bi species remaining after quenching the reactions, **1ab** was prepared according to the following adapted procedure.[7]

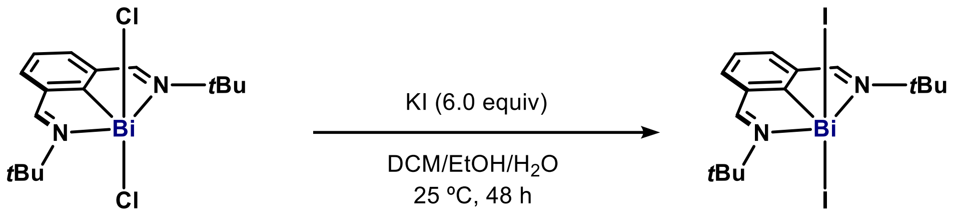

Under air, a round-bottom flask was charged with (L)Bi(III)Cl_2 **1aa** (210 mg, 0.40 mmol, 1.0 equiv), and it was dissolved in 6 mL of HPLC-grade DCM. To this mixture was added a solution of potassium iodide (400 mg, 2.4 mmol, 6 equiv) in 8 mL of HPLC-grade ethanol and 5 mL of deionized water. Upon mixing, the solution became bright yellow. The resulting biphasic mixture was vigorously stirred for 48 h. Then, it was diluted with ca. 25 mL of water and ca. 25 mL of DCM, resulting in the yellow solid fully dissolving in the organic fraction. This fraction was washed twice with water, dried over anhydrous Na_2SO_4, filtered and concentrated in vacuum, giving a yellow amorphous solid. This solid was redissolved in ca. 15 mL of boiling MeCN, and the solution was filtered through paper filter into a 100 mL Erlenmeyer flask. The solution was left cooling at room temperature for 30 min, and then for 1 h at the fridge (ca. 4 ºC). The resulting small yellow crystals were filtered in a frit, and washed twice with pentane before drying in high vacuum. This gave 101 mg (36% yield after recrystallization) of crystalline (L)Bi(III)I_2 **1ab**.

Characterization data matches the previously reported ones.[8] For reference, ^{1}H NMR in $CDCl_3$ (relatively soluble) and in MeCN-d_3 (barely soluble) is reported below.

^{1}H NMR (300 MHz, $CDCl_3$) δ 9.43 (s, 2H), 8.16 (d, *J* = 7.5 Hz, 2H), 7.93 (t, *J* = 7.5 Hz, 1H), 1.65 (s, 18H).

^{1}H NMR (300 MHz, CD_3CN) δ 9.67 (s, 2H), 8.32 (d, *J* = 7.6 Hz, 2H), 8.02 (t, *J* = 7.6 Hz, 1H), 1.60 (s, 18H).

Both (L)Bi(III)I_2 **1ab** and (L)Bi(III)Cl_2 **1aa** were shown to be inactive as catalysis in the photocatalytic coupling reaction (see Section 5 of the SI).

7. Electrochemical data

Cyclic voltammograms were collected using a 3-electrode cell consisting of a 3 mm glassy carbon working electrode, platinum wire as the counter electrode, and a bare silver wire as a pseudoreference electrode at ambient temperature in an argon-filled glovebox equipped with electrochemical outlets. Sublimed ferrocene was added as the internal reference. All potentials in V vs $Fc^{0/+}$.

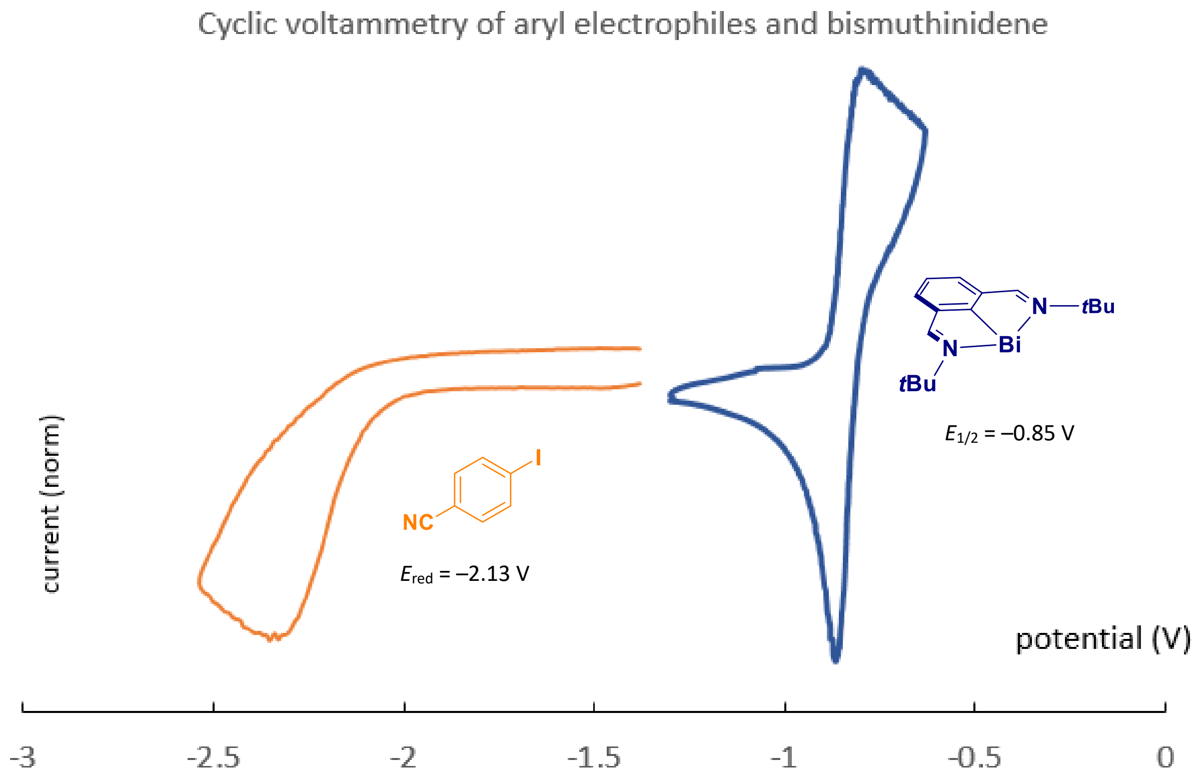

Figure S6. Cyclic voltammogram of bismuth(I) **1a** and 4-iodobenzonitrile in CH_3CN using 0.1 M $[^nBu_4N][PF_6]$ as supporting electrolyte at ambient temperature; scan rate: 100 mV/s. Potential in V vs $Fc^{0/+}$. Current is scaled for comparability.

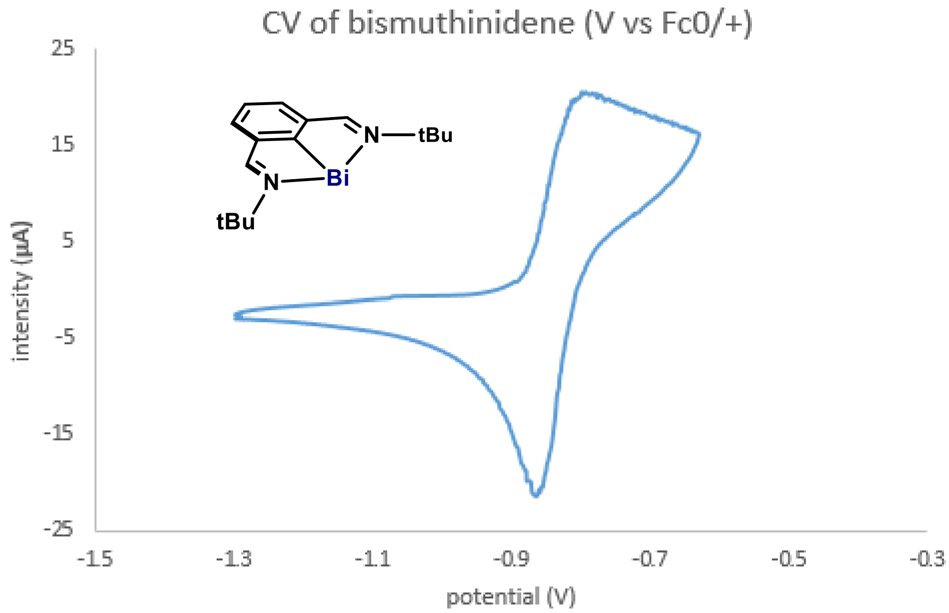

Figure S7. Cyclic voltammogram of bismuthinidene **1a** in CH_3CN using 0.1 M [$^{n}Bu_4N$][PF_6] as supporting electrolyte at ambient temperature; scan rate: 100 mV/s. Potential in V vs $Fc^{0/+}$.

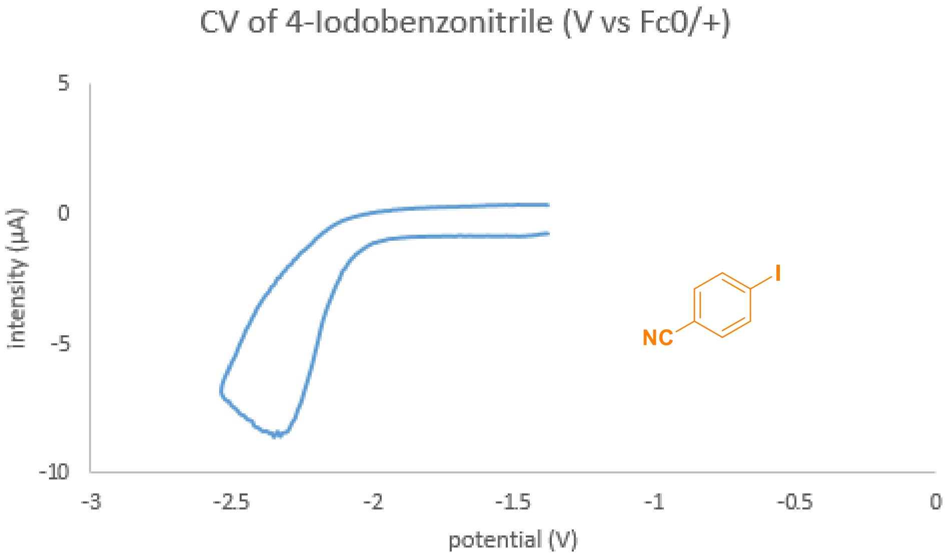

Figure S8. Cyclic voltammogram of **2a** in CH_3CN using 0.1 M [$^{n}Bu_4N$][PF_6] as supporting electrolyte at ambient temperature; scan rate: 100 mV/s. Potential in V vs $Fc^{0/+}$.

8. Photophysical properties

8.1. UV-Vis absorption and emission spectroscopy

General information

UV-Vis spectra were recorded on a Cary6000i UVVIS/NiR spectrometer, using 2 mm (*l* = 0.2 cm) Suprasil Quartz cuvettes. All measurements for bismuth complexes were done using solution of the specified concentration in anhydrous acetonitrile, stored inside an Ar-filled glovebox, and using the same solvent as blank.

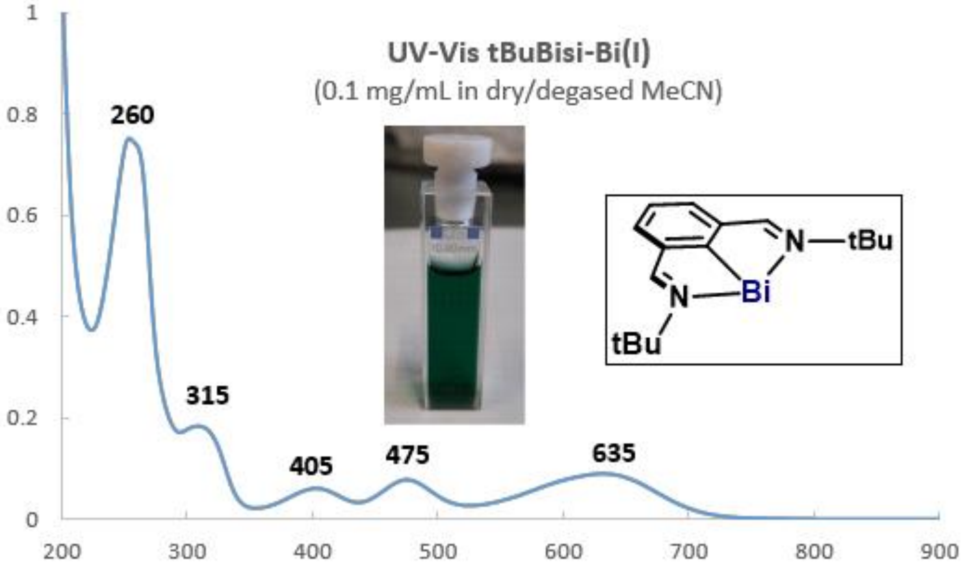

Figure S9. UV-Vis absorption spectrum of bismuthinidene **1a** in CH_3CN (0.1 mg/mL; 0.2 mM), selected absorption maxima, and physical appearance of a 1 mg/mL solution.

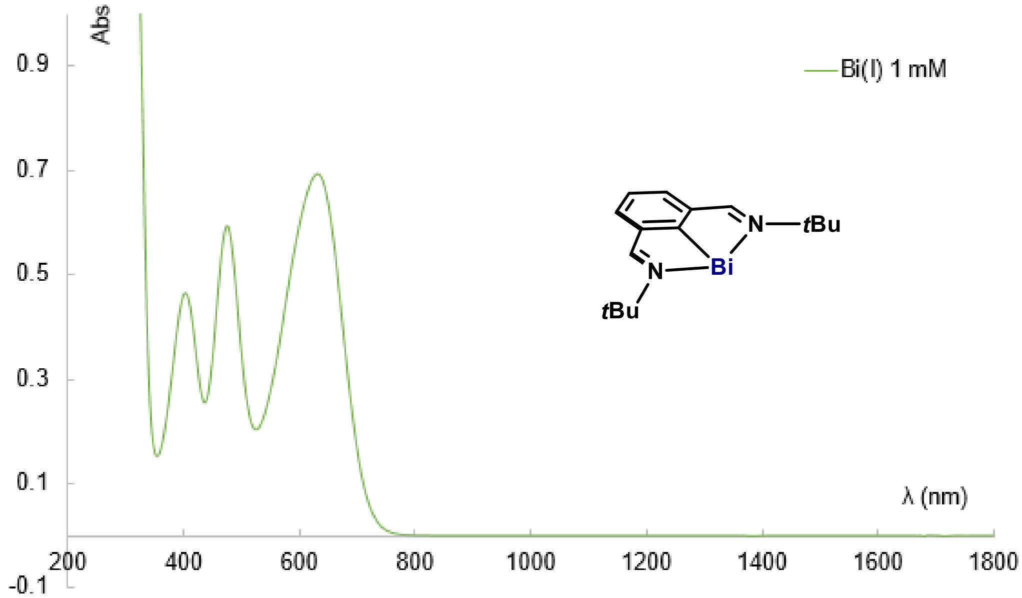

Figure S10. Absorption spectrum of bismuthinidene **1a** in CH_3CN (1 mM) from UV to NIR.

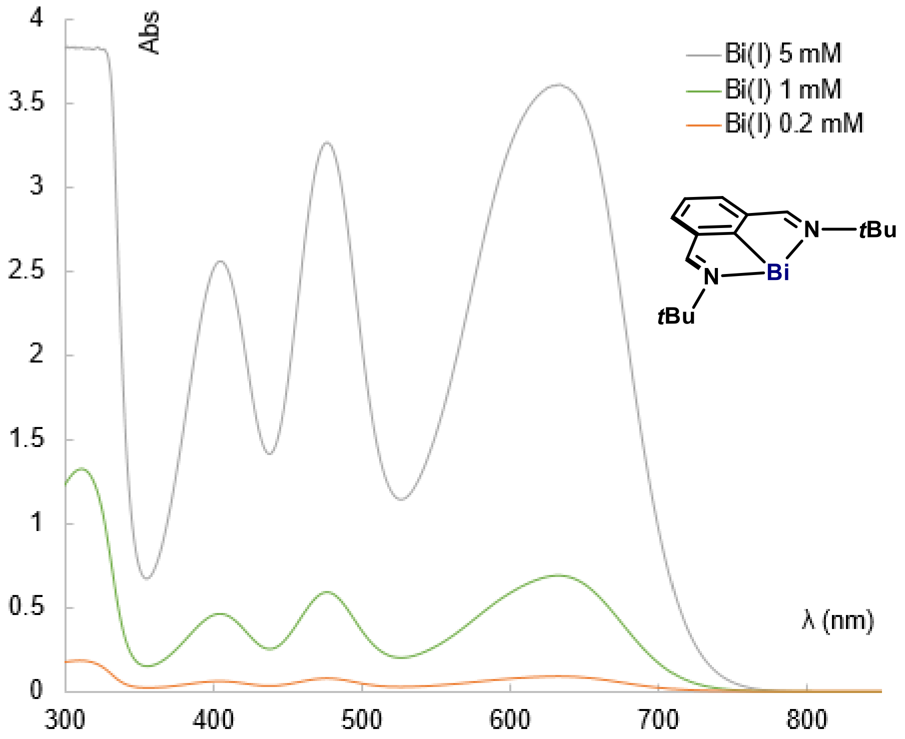

Figure S11. Absorption spectrum of bismuthinidene **1a** in CH_3CN at different concentrations between 0.2 mM and 5 mM. Higher concentrations result in a saturated spectrum.

UV-Vis absorption spectrum of aryl-iodide oxidative-addition adduct.

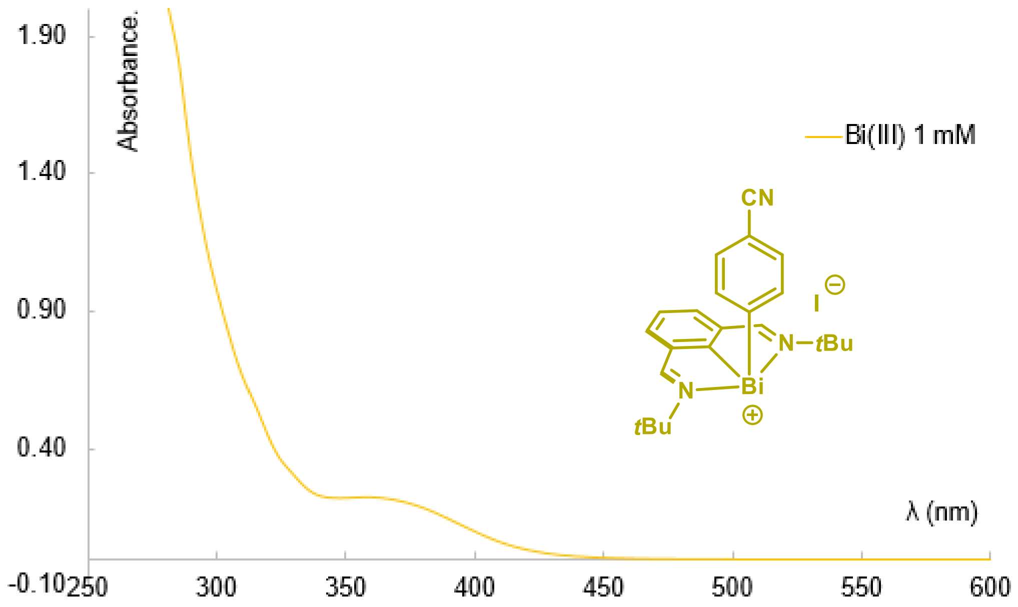

Figure S12. Absorption spectrum of aryl bismuth(III) oxidative addition adduct **3a-I** in CH_3CN (1 mM).

For completeness, an overlap of the UV-Vis of 4-iodobenzonitrile (**2a**, colorless, tails down to 400 nm) and oxidative-addition adduct **3a-I** (yellow, tails down to 450 nm) is shown below.

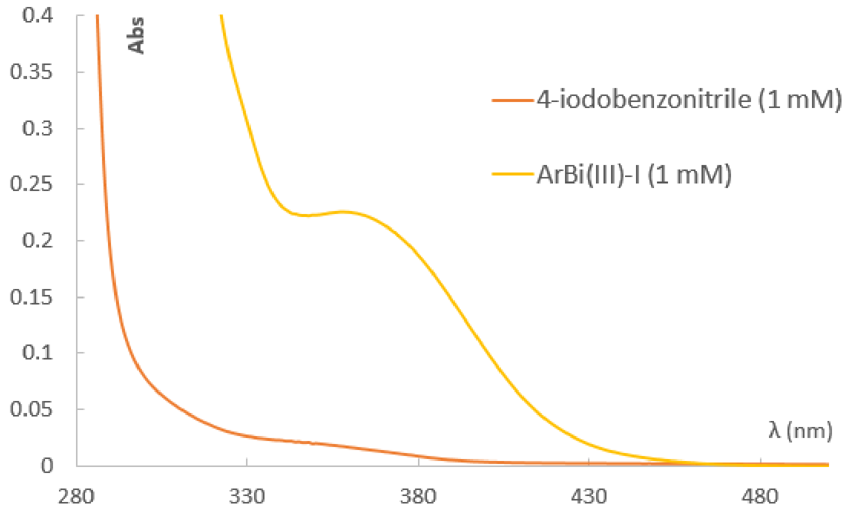

Figure S13. Overlap of the absorption spectra of **3a-I** and ArI **2a** in CH_3CN (1 mM).

UV-Vis absorption of 3a-BF_4 upon addition of iodide

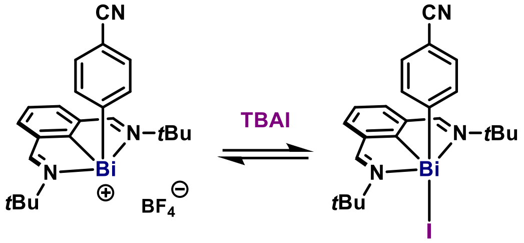

For comparison, complex **3a-BF_4** was prepared from the corresponding aryl diazonium salt following General Procedure X. UV-Vis absorbance was measured for a 1 mM solution of this colorless complex (orange trace), and also for solutions containing 1 mM of the complex and 1 mM (1 equiv) or 2 mM (2 equiv) of TBAI (tetra-*n*-butylammonium iodide) in CH_3CN (green and blue traces, respectively). The result of this addition is an appearance of the absorption band centered around 370 nm (tailing down to 450 nm) characteristic of the corresponding yellow iodide complex **3a-I** (purple trace). This is in accordance with iodide sources promoting the photoreactivity of complex **3a-BF_4**, enabled by a ligand-to-ligand charge transfer mechanism (LLCT, see computational section and reactivity studies).

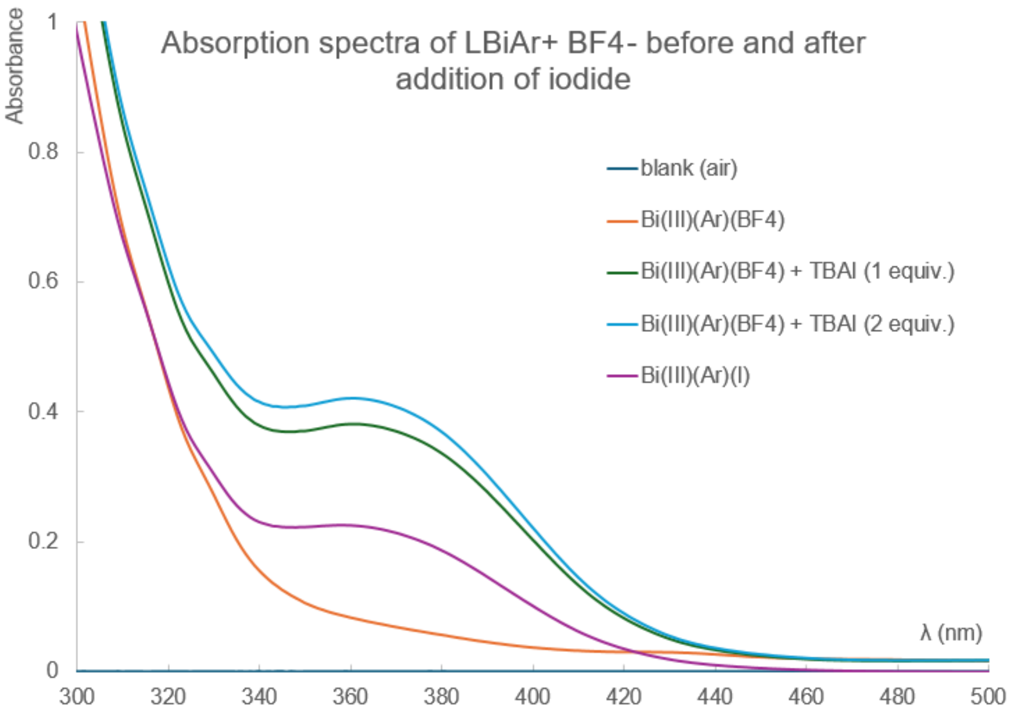

Figure S14. Overlap of the absorption spectra of **3a-I** (purple) **3a-BF_4** (orange) and **3a-BF_4** after the addition of 1 equiv (green) or 2 equiv (blue) of TBAI (tetra-*n*-butylammonium iodide) in CH_3CN (ca. 1 mM for bismuth).

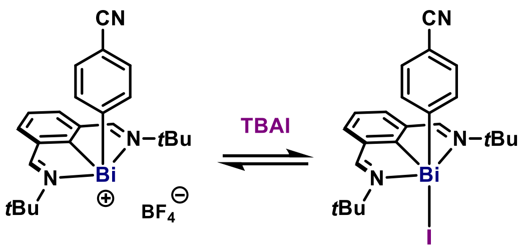

The following graph shows even more clearly the effect of adding an iodide source, by showing the *difference* in UV-Vis absorbance between complex **3a-BF$_4$** (baseline) and the same complex after the addition of 1 equiv (navy blue trace) or 2 equiv (green trace) of TBAI (tetra-*n*-butylammonium iodide). The increase in absorbance, especially in the 400 – 450 range, is responsible for the absorption of blue light under the reaction conditions.

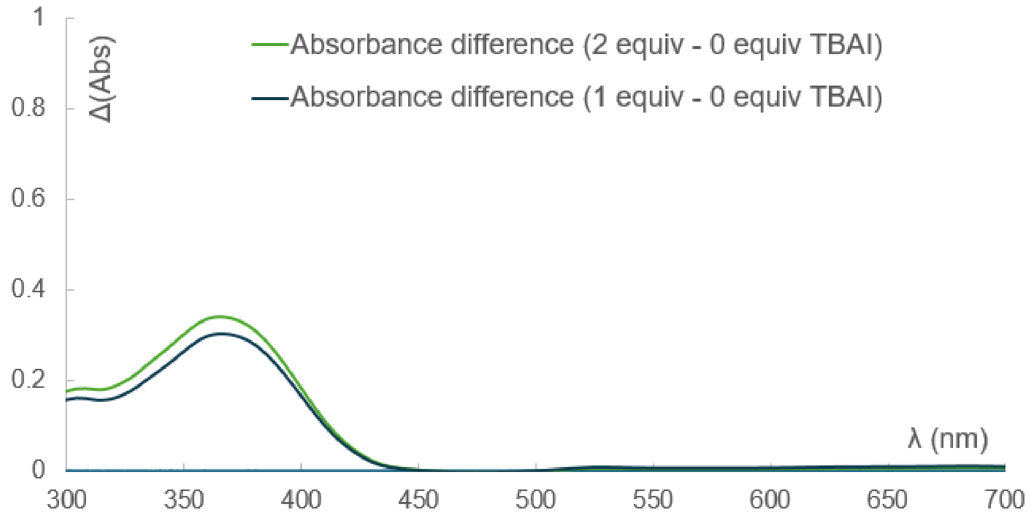

Figure S15. Absorbance difference between **3a-BF$_4$** and the same complex after the addition of 1 equiv (blue) or 2 equiv (green) of TBAI (tetra-*n*-butylammonium iodide) in CH_3CN (1 mM for bismuth).

Overlap of the absorption of 1a and 3a and the emission of the blue and red LED

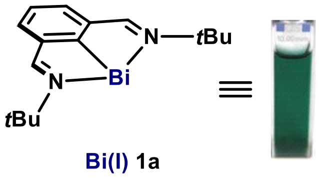

Bi(I) 1a

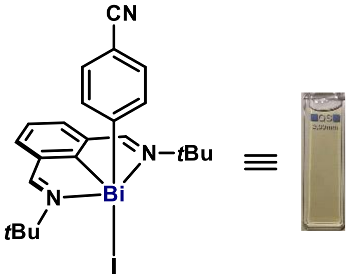

Bi(III) 3a

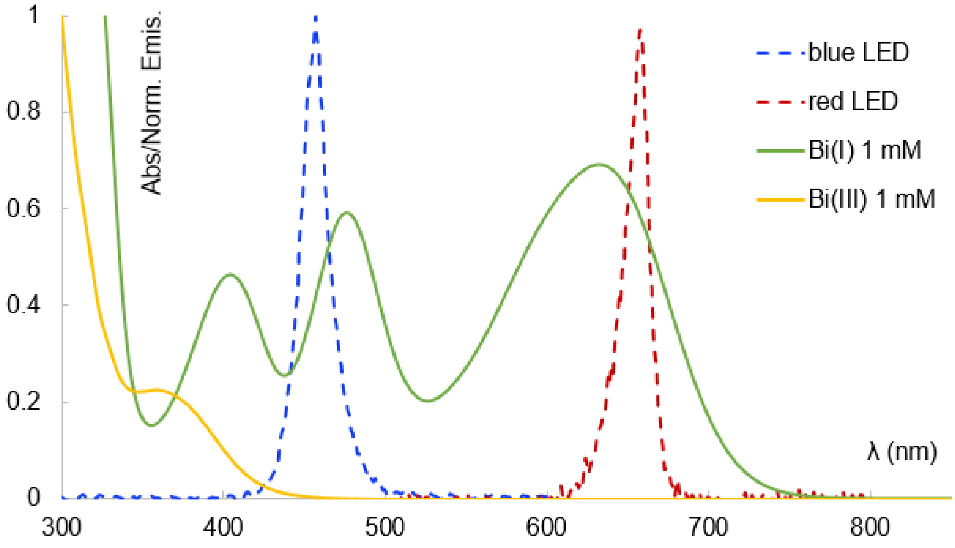

Figure S16. Overlap of the absorption spectra of Bi(I) **1a** and Bi(III) **3a** in CH_3CN (1 mM) and the emission spectra of the two light sources (blue and red LED) employed in this study.

Bismuth(I) complex **1a** absorbs and can be photo-activated by either blue or red light (green trace). However, aryl-bismuth(III) complex **3a** (yellow trace) is transparent to red light, and can only be activated by blue light, which triggers the homolysis and allows establishing a catalytic cycle.

UV-Vis absorption of a mixture of bismuthinidene and electrophile

In order to evaluate potential charge-transfer interaction between the two reagents in solution, we conducted UV-Vis absorption analysis of a solution of bismuthinidene **1** (0.1 mg/mL in MeCN), a solution of 4-iodobenzonitrile (0.2 mg/mL in MeCN) and a 1:1 mixture of the two solutions (0.05 mg/mL of **1**, 1 equiv, and 0.1 mg/mL of 4-iodobenzonitrile, 4 equiv).

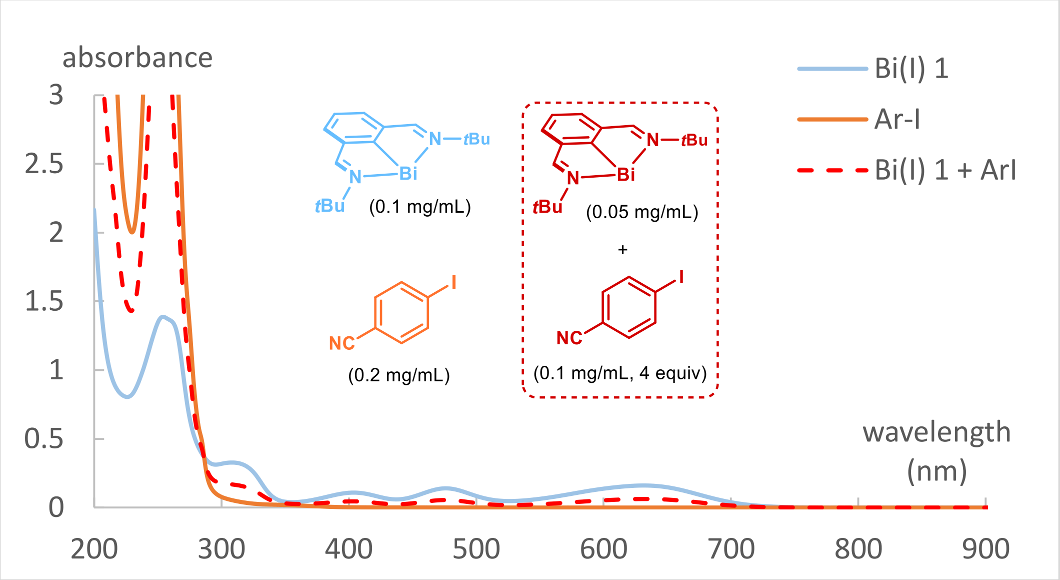

Figure S17. UV Vis absorption spectra of **1**, an electrophile, and a mixture of both.

We found no significant shift in the absorption bands. For easy visualization, a normalized zoomed in version of the spectrum is attached below.

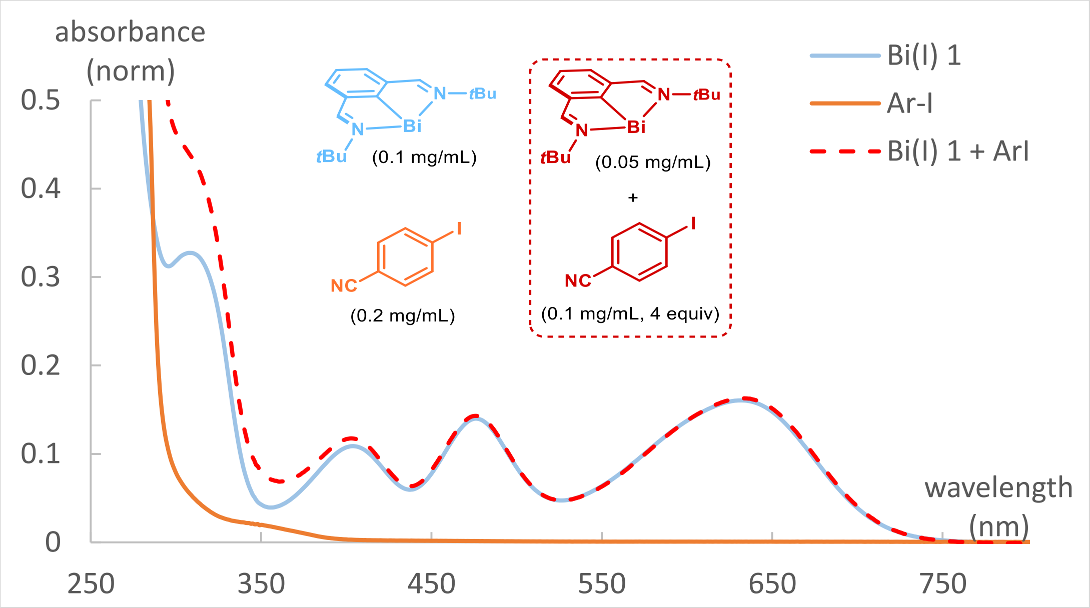

Figure S18. Zoomed in UV Vis absorption spectra of **1**, an electrophile, and a mixture of both.

Thus, no charge transfer of EDA complex formation can be observed.

A similar analysis was performed by ^{1}H NMR in MeCN-d_3.

A 0.01 M solution of bismuthinidene **1** in MeCN-d_3 (4.5 mg/mL, 0.01 mmol) of bismuthinidene **1** in MeCN-d_3 was first prepared and measured by NMR. A second 0.01 M solution of 4-iodobenzonitrile in MeCN-d_3 (2.3 mg/mL, 0.01 mmol) was prepared and measured by NMR. Then, a 1:1 mixture of the two solutions was prepared and also measured by NMR.

Again, no significant shift or broadening of the signals that might suggest a bonding interaction was observed.

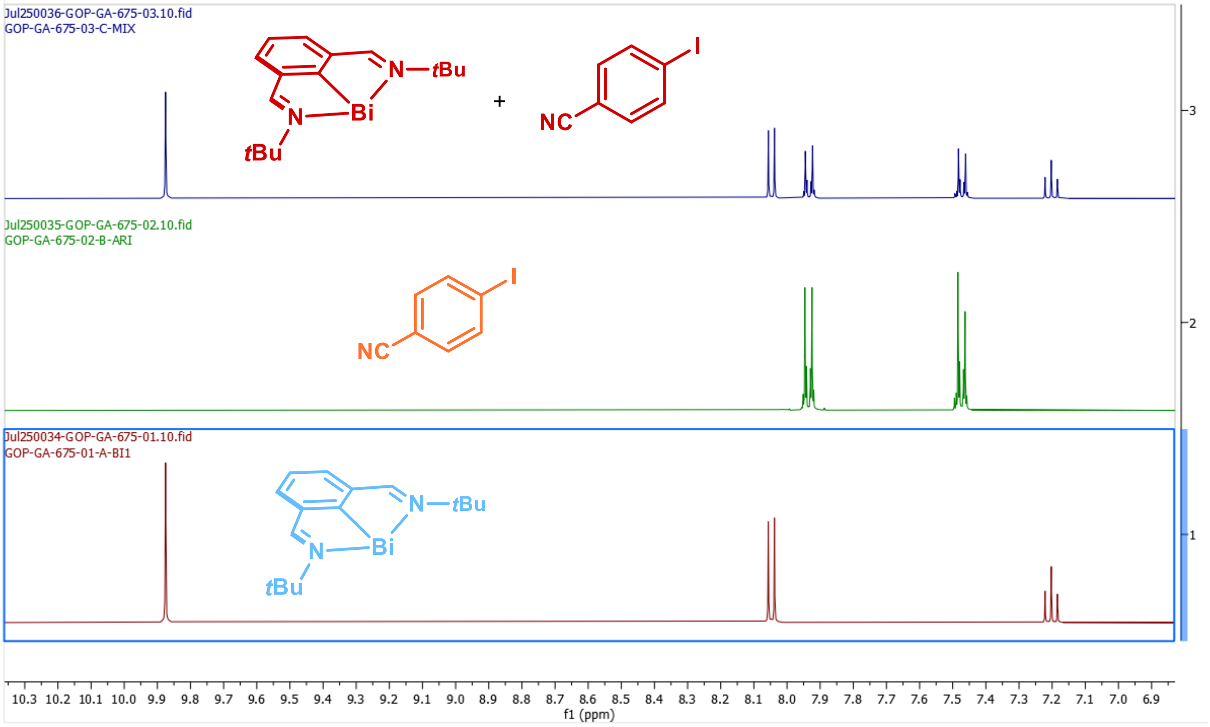

Figure S19. ^{1}H NMR spectra of **1** (bottom), an electrophile (middle), and a mixture of both (top).

8.2. Ferrioxalate actinometry and quantum yield determination

A. Determination of photon flux

The photon flux of the blue-LED photoreactor used for the catalysis was determined following a reported procedure for standard ferrioxalate actinometry.[9]

All solutions used in this procedure were freshly prepared and protected from ambient light as much as possible (room lights off and containers wrapped with aluminum foil or black tape).

Preparation of stock solutions

1) 10 mL of a buffered phenanthroline was prepared by dissolving 10 mg of 1,10-phenanthroline and 2.25 g of sodium acetate in 10 mL of aqueous H_2SO_4 0.5 M (prepared by dissolving 2.8 mL of concentrated 97% H_2SO_4 in deionized water, and adjusting the volume to 100 mL).
2) 10 mL of a 0.15 M solution of ferrioxalate was prepared by dissolving 0.74 g of ($[K_3Fe^{III}(C_2O_4)_3]•3H_2O$) in 10 mL of aqueous H_2SO_4 0.05 M (prepared by diluting ten-fold the 0.5 M solution prepared in step 1with deionized water).

Determination of photon flux

2 mL of the 0.15 M ferrioxalate solution (2) were added to a 10 mL culture tube (the same vessel and solvent volume used for the catalytic reactions). The vial was irradiated in the blue-LED photoreactor set up at λ_{max} = 457 nm for 15 seconds. After stopping the irradiation, the vial was protected again from ambient light, before 0.35 mL of the buffered phenanthroline solution (1) were added. The solution was allowed to rest for 1 h to allow the Fe^{2+} ions to fully coordinate to phenanthroline. Then, the solution was transferred to a 2 mm cuvette, and the UV-Vis absorption spectrum was recorded (blue trace).

This procedure was repeated side-by-side with another identical sample which was stored in the dark instead of submitting it to blue LED irradiation. UV-Vis absorption spectrum was also recorded to determine the background formation of Fe^{2+} ions (black trace).

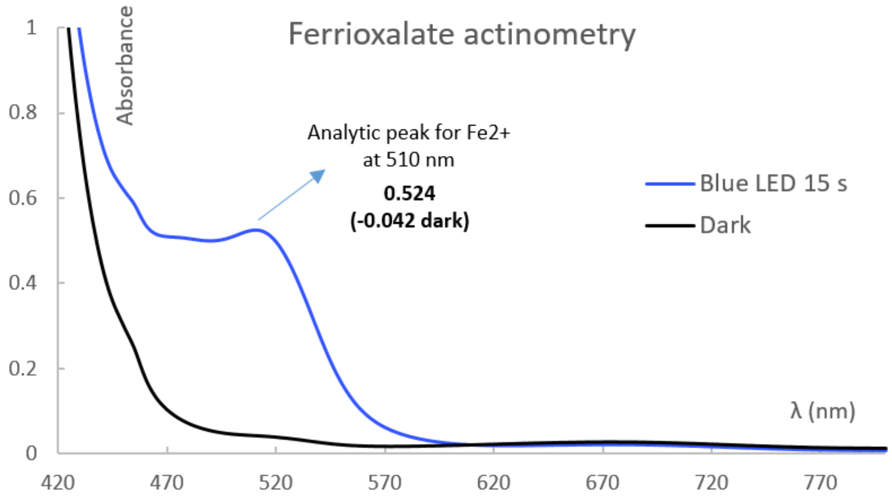

The absorption at λ_{max} = 510 nm was used as analytical peak to determine the concentration of Fe^{2+} ions formed as product of the photoreaction. The difference in absorbance at 510 nm between the blue-LED-irradiated reaction and the reaction in the dark was determined, ΔA.

Two replicates of these two experiments were performed, giving an average value of ΔA = 0.4865.

The amount of Fe^{2+} formed was calculated according to the following equation:

$$mol\ Fe^{2+} = \frac{V \cdot \Delta A}{l \cdot \varepsilon}$$

Where V is the total volume of the solution (2.0 + 0.35 mL = 2.35 mL = 0.00235 L), ΔA is the aforementioned average difference in absorbance at 510 nm (Figure Snn between the irradiated ferrioxalate solution, blue trace, and the one kept in the dark, black trace), *l* is the path length of the cuvette (0.2 cm) and ε is the molar absorptivity coefficient at 510 nm (11.110 L mol^{-1} cm^{-1}).[9]

$$mol\ Fe^{2+} = \frac{(0.00235\ L) \cdot (0.4865)}{(0.2\ cm) \cdot (11100\ L\ mol^{-1}\ cm^{-1})} = 5.1499\ \times 10^{-7} mol$$

The fraction of 467 nm light absorbed by the ferrioxalate actinometer (f_{Fe}) was calculated using the following equation:

$$f_{Fe} = 1 - 10^{-A}$$

Where A is the absorbance of the Fe^{3+} oxalate at 467 nm.

$$f_{Fe} = 1 - 10^{-0.21} = 0.38$$

The photon flux was calculated according to the following equation:

$$photon\ flux = \frac{mol\ Fe^{2+}}{\Phi_{\boldsymbol{Fe}} \cdot t \cdot f_{Fe}}$$

Where Φ_{Fe} is the quantum yield for the ferrioxalate actinometer (1.1 for a 0.15 M solution at λ_{max} = 456 nm), *t* is the time of irradiation (15 s) and f_{Fe} is the fraction of light absorbed by Fe^{3+} at 457 nm.

$$photon\ flux = \frac{(5.1499\ \times 10^{-7} mol)}{(1.1) \cdot (15\ s) \cdot (0.38)} = 1.0175\ \times 10^{-7} einstein/s$$

Fraction of 457 nm light absorbed by the Bi(I) catalyst

First, the fraction of 457 nm light absorbed by the bismuth(I) catalyst **1a** was determined to be $f_{Bi} \approx 1$ according to the following equation:

$$f_{Bi} = 1 - 10^{-A}$$

The absorbance of bismuthinidene **1a** at a 5 mM concentration in MeCN is 2.386.

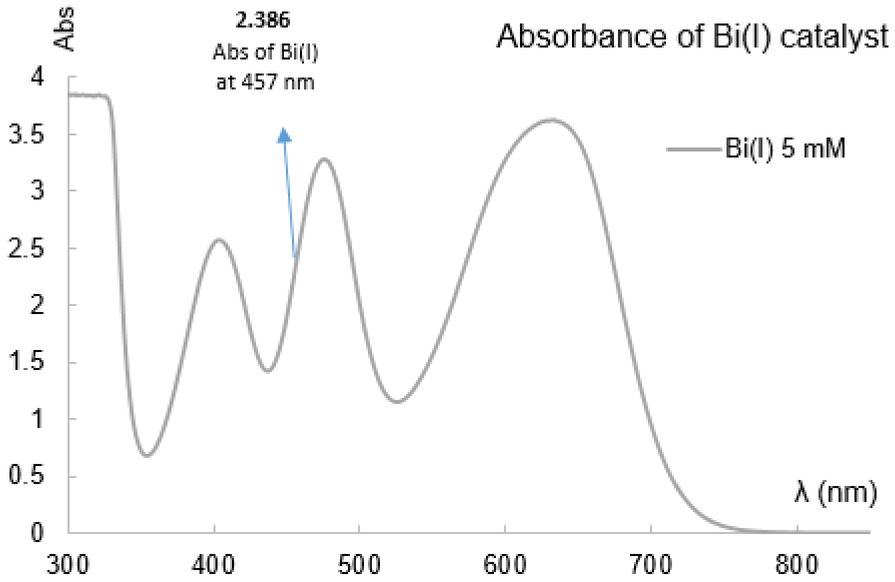

$$f_{Bi} = 1 - 10^{-2.386} = 0.996$$

In the catalytic reactions, a concentration of 10 mM of **1a** is employed, leading to saturated absorbance spectra (A > 4 at 467 nm), resulting in a fraction of light absorbed of $f_{Bi} \approx 1$.

Determination of the quantum yield of the catalytic reaction

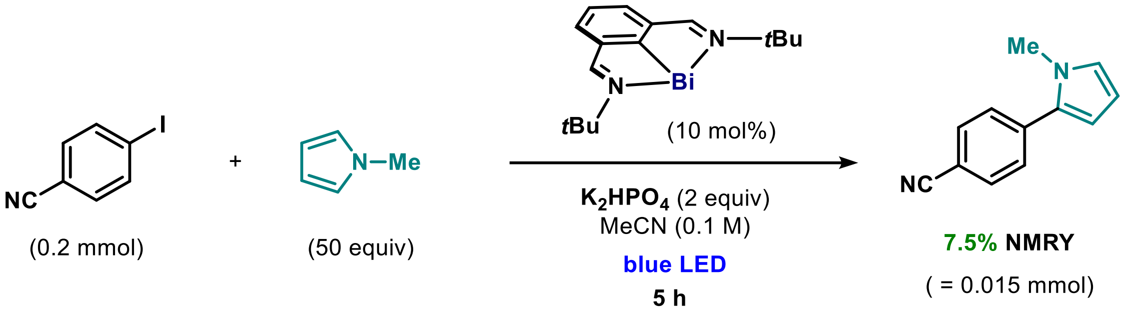

According to the general procedure, in an Ar-filled glovebox, a culture tube with a magnetic stirring bar was charged with 4-iodobenzonitrile (0.20 mmol, 46 mg, 1.0 equiv), dipotassium phosphate (0.40 mmol, 70 mg, 2.0 equiv), *N*-methylpyrrole (10 mmol, 0.81 g, 0.89 mL, 50 equiv) and acetonitrile (2.0 mL, 0.10 M). Then, bismuthinidene **1a** (10 mol%, 9.0 mg) was added, before the vial was closed with the corresponding screw-cap and further sealed with parafilm. The vial was taken out of the glovebox and stirred under blue LED (457 nm, same exposure as for the actinometry) for 5 h. After this time, 1,3,5-trimethoxybenzene (34 mg, 0.20 mmol, 1.0 equiv) was added as internal standard, the solvent was removed in high vacuum, and the crude mixture was analyzed by ^{1}H NMR in $CDCl_3$.

The NMR yield obtained was 7.5%, which translates in the formation of 0.015 mmol of product.

The quantum yield of the catalytic reaction (Φ) was determined according to the following equation:

$$\Phi = \frac{mol\ product}{photon\ flux \cdot t \cdot f_{Bi}}$$

Where *mol product* is the moles of product formed within the reaction time (0.000015 mol), *photon flux* was determined through the ferrioxalate actinometry (1.0175 x10^{-7} einstein/s), *t* is the reaction time (5 h = 18.000 s) and f_{Bi} is the fraction of 457 nm light absorbed by the bismuth(I) photocatalyst (ca. 1).

$$\Phi = \frac{(0.000015\ mol)}{(1.0175\ \times 10^{-7}\ einstein/s)\ \cdot (18000\ s) \cdot (1)} = 0.00819$$

The quantum yield for the catalytic reaction was determined to be **0.82%**. Thus, a free radical chain-reaction pathway cannot be identified.

9. Mechanistic experiments

9.1. Effect of iodide on the stoichiometric C-H arylation of *N*-methyl pyrrole

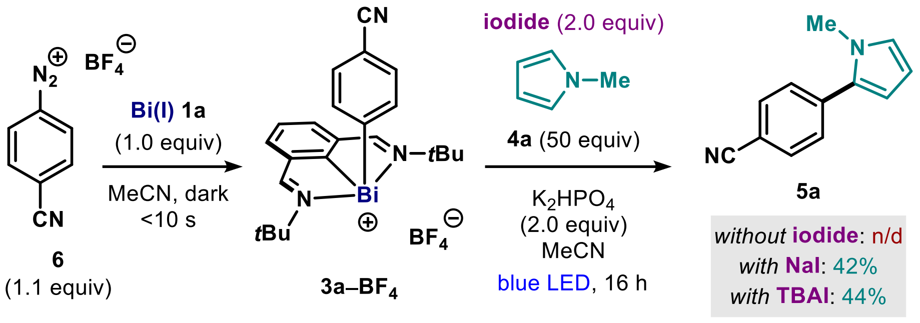

While working in an argon-filled glovebox, a 4 mL scintillation vial equipped with a magnetic stir bar was charged with Bi(I) complex (45 mg) and anhydrous MeCN (1.0 mL). To the stirring solution was added a solution of p-cyanophenyldiazonium tetrafluoroborate (22 mg) in MeCN (1.0 mL) in a dropwise fashion. At the end of the addition, the reaction color changed from dark green to pale orange. This solution was complemented with NMP (406 mg, 0.44 mL) and 0.56 mL anhydrous MeCN. After swirling the solution to ensure homogeneity, 0.60 mL aliquots of this stock solution were dispensed into three 10 mL thick-walled culture tubes, each containing a Teflon-coated magnetic stir bar and K_2HPO_4 (7.0 mg, 0.040 mmol, 2.0 equiv). The three reaction vessels were charged with additives according to the following legend:

Reaction 1: no additive

Reaction 2: + NaI (6.0 mg, 0.040 mmol, 2.0 equiv)

Reaction 3: + $[Bu_4N][I]$ (15 mg, 0.040 mmol, 2.0 equiv)

The culture tubes were capped with Teflon-lined caps, which were further secured with Parafilm. The setup was subjected to blue light irradiation (457 nm LED strip) at room temperature with constant stirring. After 24 h, the reaction vessels were removed from the setup and the contents were concentrated to dryness under high vacuum (the dried residue was heated gently to ca. 40 °C to remove all residual NMP and MeCN). The crude material in the culture tubes was complemented with a stock solution of trimethoxybenzene (3.4 mg, 0.020 mmol, 1.0 equiv) in $CDCl_3$ (0.65 mL). The resulting suspension was sonicated for 1 min before being filtered into an NMR tube which was submitted for ^{1}H NMR analysis.

9.2. Stoichiometric experiments to differentiate between catalytic and radical chain pathways for product formation

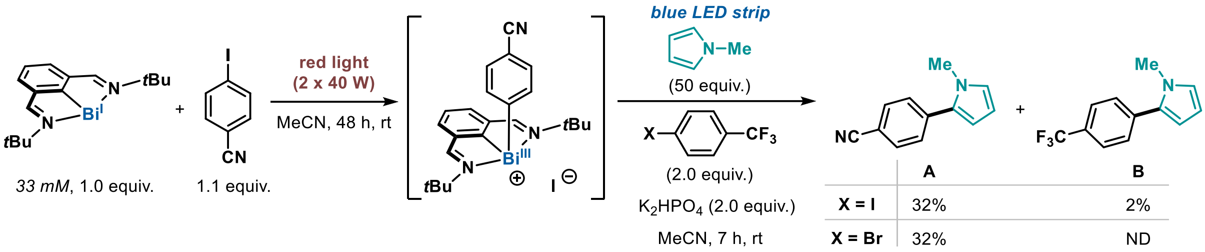

While working in an argon-filled glovebox, a flame-dried 10 mL thick-walled culture tube equipped with a Teflon-coated magnetic stir bar was charged with Bi(I) complex (0.020 mmol, 9.0 mg, 1.0 equiv), *p*-cyanoiodobenzene (0.022 mmol, 5.0 mg, 1.1 equiv), and anhydrous MeCN (0.60 mL). The tube was capped with a Teflon-lined cap and the cap further secured with Parafilm. The tube was taken out of the glovebox and subjected to red-light irradiation (2 x 660 nm LED PR160L Kessil lamps at 100% intensity) with constant stirring. After 48 h, the color of the reaction had changed from dark green to pale yellow, indicating full conversion of the Bi(I) complex.

The reaction tube was removed from the setup and ported back into an argon-filled glovebox. The resulting solution was complemented with *N*-methyl pyrrole (81 mg, 1.0 mmol, 50 equiv), K_2HPO_4 (7.0 mg, 0.040 mmol, 2.0 equiv), and *p*-(trifluoromethyl)iodobenzene (11 mg, 0.040 mmol, 2.0 equiv) or *p*-(trifluoromethyl)bromobenzene (9.0 mg, 0.040 mmol, 2.0 equiv). The tube was capped with a Teflon-lined cap and the cap further secured with Parafilm. The tube was taken out of the glovebox and subjected to blue-light irradiation (457 nm LED strip) at room temperature with vigorous stirring. After 7 h, the tube was removed from the setup and the contents were concentrated to dryness under high vacuum (the dried residue was heated gently to ca. 40 °C to remove all residual NMP and MeCN). The crude material was complemented with a stock solution of 1,3,5-trimethoxybenzene (3.4 mg, 0.020 mmol, 1.0 equiv) in $CDCl_3$ (0.65 mL). The resulting suspension was sonicated for 1 minute before being filtered into an NMR tube which was submitted for ^{1}H NMR analysis.

9.3. Miscellaneous experiments

Influence of light wavelength in the stoichiometric oxidative addition of aryl iodides

The initial observation that prompted us to explore the reactivity of aryl-bismuth(III) complexes under blue light was the fact that, whereas under red- or green-LED irradiation the stoichiometric reaction occurs cleanly, under blue light, decomposition of the oxidative-addition adducts occurs a significant rate. Evaluation of this stoichiometric reaction with different light wavelengths is shown below.

The reaction was followed using irradiation from different light colors, with LEDs with an estimated maximum output power of 20 W. The number of photons reaching each reaction may vary due to fabrication of the different light strips, and slight differences in reaction set-up. Hence, this only aims to be a qualitative comparison on selectivity. Reaction performed with 0.010 mmol of Bi(I) and 0.020 mmol of 4-iodobenzonitrile in 0.6 mL of MeCN-d_3 (0.0166 M), in an NMR tube. Yields determined by ^{1}H NMR using 1,3,5-trimethoxybenzene as internal standard.

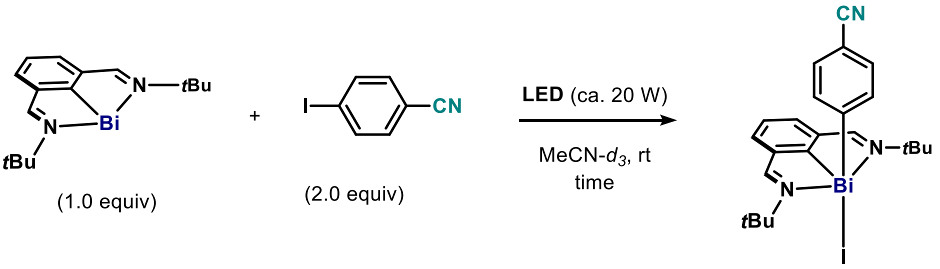

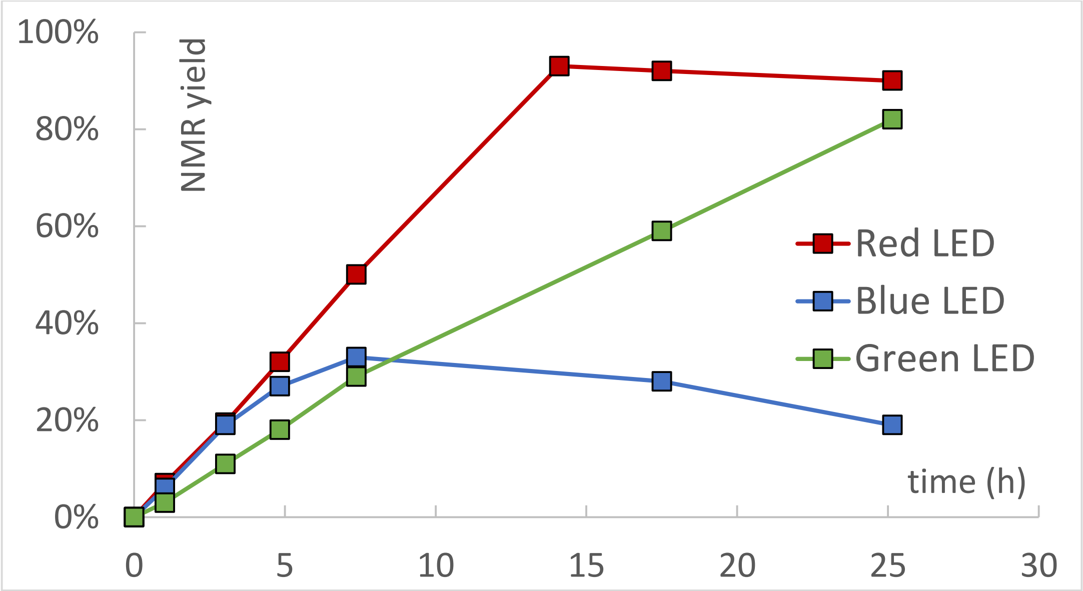

Figure S20. Reaction profiles using different light wavelengths.

For a closer comparison, blue (457 nm, 100 cm/140 LEDs, max. output ca. 19 W; max. luminous flow 400 lumen), green (550 nm, 100 cm/140 LEDs, max. output ca. 20 W; max. luminous flow 1360 lumen) and red (650 nm , 100 cm/140 LEDs, max. output ca. 21 W; max. luminous flow 1000 lumen) LED-strip irradiation was performed with 100 cm of 24V DC LED strips strapped around a 15-cm glass crystalizing dish. These LED strips were purchased from LEDs24. Nevertheless, the amount of light reaching the sample with each LED is likely not the same.

10. Computational analysis

Computational details

Quantum chemical calculations were performed using a development version of ORCA.[10] Geometry optimizations were performed using X2C relativistic approach,[11] with the B3LYP functional,[12] the x2c-TZVPPall-2c basis set for all the atoms and the x2c/J auxiliary basis set were employed.[13] Dispersion interactions were taken in account by using the D3 correction with Beck-Johnson damping function.[14] Solvation effects of acetonitrile were modeled with the SMD method.[15] Frequency calculations revealed that all optimized geometries were local minima with no imaginary frequencies.

Time-dependent density functional theory (TDDFT) calculations were performed on the DFT-optimized structures using the same level of theory reported above. Spin-orbit coupling (SOC) was treated using the mean-field SOC Hamiltonian, including picture change effects.[16] A total amount of 100 roots were computed including both singlet and triplet transitions. Subsequently, the calculated states were mixed through SOC to obtain the SOC-corrected energies transitions.

10.1 Structural Isomers of complex **3a-I**

Two structural minima of complex **3a-I** differing for the location of the I^- ligand (Figure S21) were investigated. In one case, the I^- ligand is coordinated *trans* with respect to the aryl group and sitting perpendicular to the plane of the pincer scaffold (**3a-I-axial**, I-Bi-C1 angle ≈ 167° in Figure S21a) while in the second case the iodine is *cis* with respect to the aryl group and sitting in the plane of the pincer scaffold (**3a-I-eq**, I-Bi-C1 angle ≈ 91° in Figure S21b). We found that **3a-I-axial** provides a slightly lower electronic energy compared to the **3a-I-eq** model. TDDFT calculations (Figure S22) show that **3a-I-eq** displays one transition with non-negligible oscillator strength (f_{osc}=0.024) at 26509 cm^{-1} (λ=377 nm). Such transition occurs at higher energies compared to when the I^- is in *axial* position (*vide infra*) and it is due to HOMO to LUMO excitations. However, the LUMO in **3a-I-eq** contains contributions from the Bi atom meaning that HOMO-LUMO transition cannot be defined as a LLCT for this isomer. We can speculate that if the I^- is coordinating the Bi within the plane of the *N*,*C*,*N* pincer, the blue-light-promoted homolytic Bi–C1 cleavage of the axial aryl group is less effective. This convince us to use the **3a-I-axial** as representative model of the experimental **3a-I** complex.

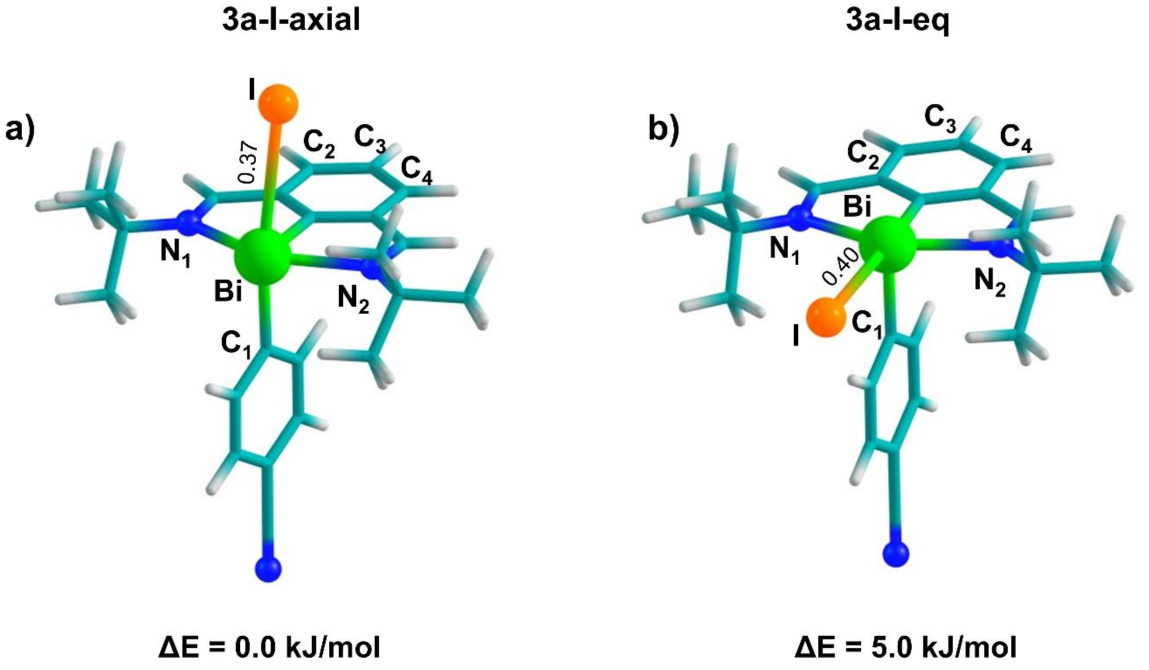

Figure S21. Optimized geometric structure of the isomers of complex **3a-I**. a) **3a-I-axial** with I-Bi-C1 angle ≈ 167° b) **3a-I-eq** I-Bi-C1 angle ≈ 91°. The relative electronic energy for the two configuration is reported at the bottom. The Bi-I bond length is given in nm.

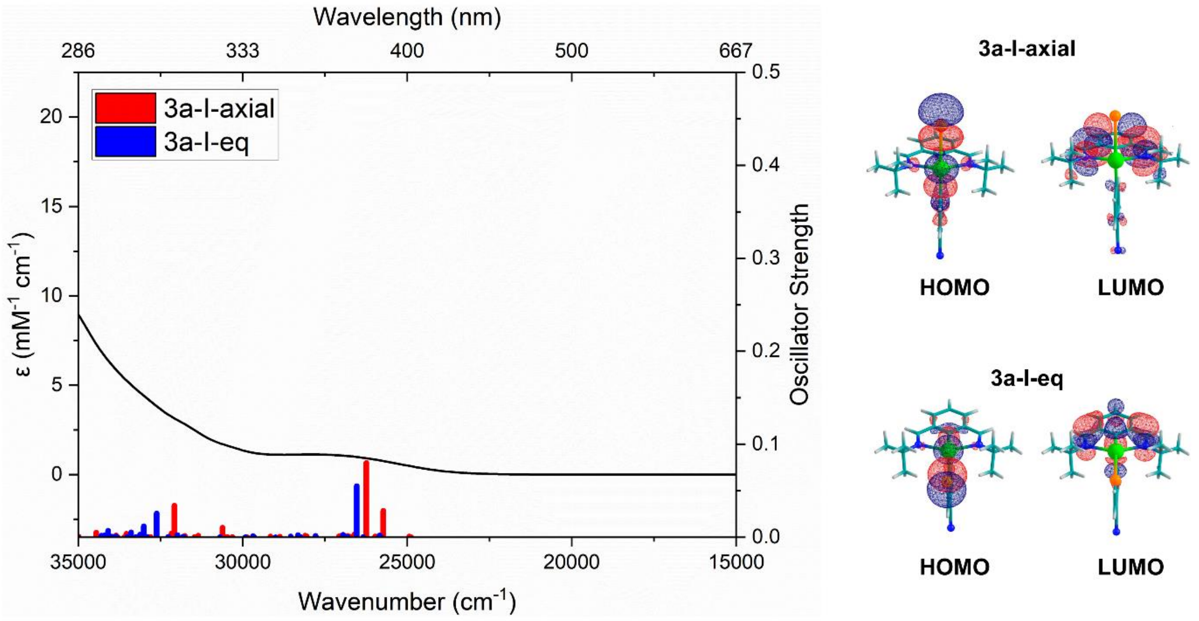

Figure S22. Experimental UV-Vis spectrum of **3a-I** (black trace) together with SOC-corrected TDDFT excited transitions for the isomer **3a-I-axial** (red bars) and the isomer **3a-I-eq** (blue bars). The natural transition orbitals (NTOs) representing the most intense transitions (HOMO→LUMO) are reported.

10.2 TDDFT details

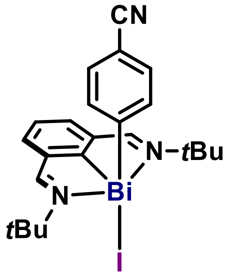

Inspection of UV-vis spectrum for complex **3a-I** reveals a broad absorption band spread from 27000 to approximately 25000 cm^{-1} ($\lambda \approx 370$-400 nm) which tails into the emission of the blue-LED light employed in the experimental catalytic study. TDDFT calculations on the optimized model structure of **3a-I** pinpoint the presence of two transitions with non-negligible oscillator strength falling at 25007 cm^{-1} (λ=399 nm, f_{osc}=0.020) and 25654 cm^{-1} (λ=390 nm, f_{osc}=0.053). Such transitions involve HOMO to LUMO excitations, as shown in **Figure S21**. Assuming that the z-axis is passing through the Bi-I bond, the HOMO is predominantly composed by the contributions from the I $5p_z$ as well as small components from the σ orbitals of the benzonitrile group. On the contrary, the LUMO is characterized by antibonding π orbitals spread over the *N*,*C*,*N*-pincer ligand (see also Mulliken population of the orbitals in **Table S1**). Hence, we can assign the absorption covering the blue light area to ligand-to-ligand charge transfer (LLCT).

Table S1. Mulliken orbital population calculated for HOMO and LUMO orbitals of complex **3a-I**. The contribution of each atom to the molecular orbitals is given in percentage.

Atom	HOMO	LUMO
Bi	7.0	< 1.0
I	76.4	< 1.0
N_1	< 1.0	8.5
N_2	< 1.0	10.8
C_1	11.5	1.3
C_2	< 1.0	16.7
C_3	< 1.0	17.2
C_4	< 1.0	5.7

Notably, calculations on the analog model without the iodide ligand (with a BF_4^- counteranion instead) show no transitions around blue light regime (**Figure S22**), correlating with the experimental observations, both in terms of UV-vis absorption analysis (see SI Section 8.1) and reactivity (see SI Section 9.1). This highlights the key role of the iodide in the photoreactivity and in catalysis.

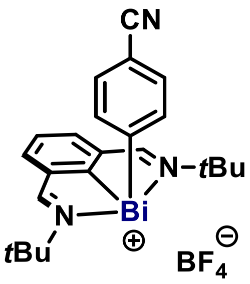

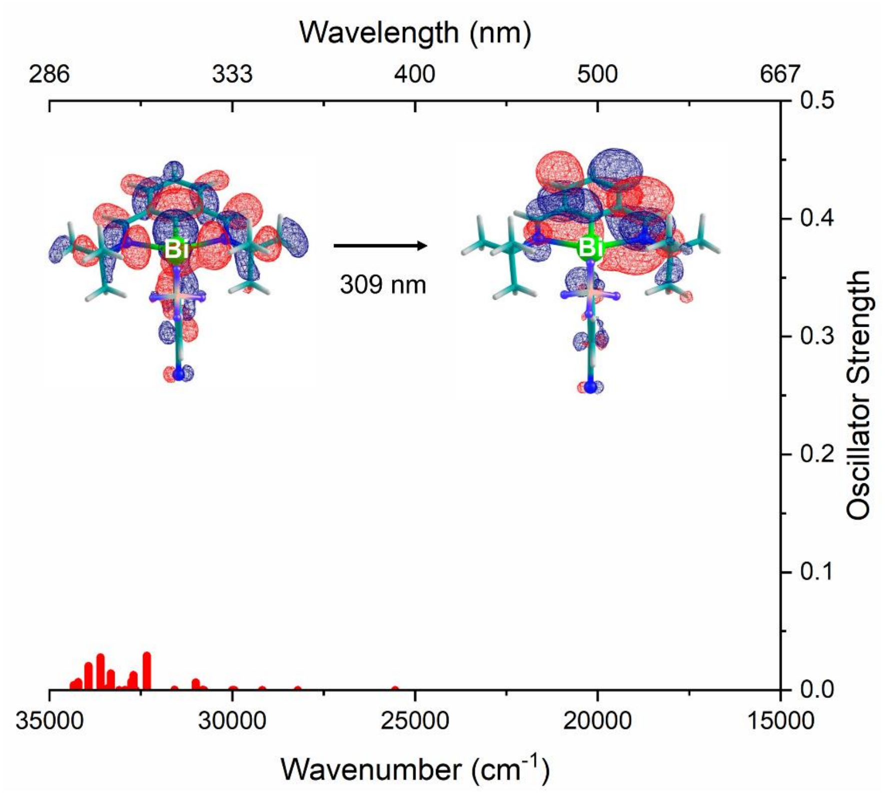

Figure S23. SOC-corrected TDDFT excited transitions (red bars of optimized model of pincer Bi(I) complex with BF_4 counterion instead of I^- ligand. The natural transition orbitals (NTOs) representing the transitions falling around 32000 cm^{-1} are reported.

10.2 Calculation of BDFE

Bond dissociation free energies (BDFEs) for Bi-I and Bi-C_1 bonds in complex Z were calculated as the change of the Gibbs free energy at 298 K of the following reactions:

$$LBi\text{-}I \rightarrow LBi^{\cdot} + I^{\cdot}$$

$$LBi\text{-}C_1 \rightarrow LBi^{\cdot} + C_1^{\cdot}$$

BDFEs were computed on the DFT optimized structures by using the electronic energy calculated at CCSD(T) with the DLPNO scheme and the vibrational corrections obtained at B3LYP level of theory.[17]

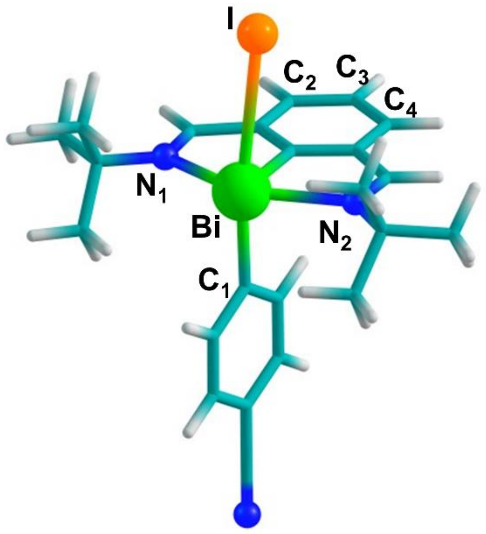

Table S2. Calculated bond dissociation free energies (BDFEs) at 298 K for Bi-I and Bi-C_1 bonds in complex **3a-I**.

**Bonds in Complex Z**	**BDFE** in kJ/mol (kcal/mol)
Bi-I	276.0 (66.0)
Bi-C_1	218.5 (52.3)

This shows the thermodynamically higher feasibility of homolytic cleavage for the Bi-C bond in contrast to the C-I bond upon blue-light absorption (blue light employed centered at 467 nm = 2.65 eV = 61.1 kcal/mol).

10.3 Cartesian coordinates

Complex **3a-I-axial**

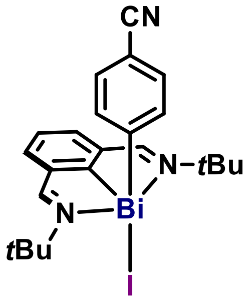

C	5.442592000	8.134877000	2.308570000
H	5.768437000	8.739001000	3.157956000
C	4.743640000	8.724447000	1.249554000
H	4.528002000	9.792867000	1.273517000
C	4.311002000	7.954658000	0.164520000
H	3.757203000	8.418175000	-0.654604000
C	4.590575000	6.575818000	0.133916000
C	5.305583000	5.999033000	1.183206000
Bi	5.746589000	3.824055000	1.129520000
N	6.626984000	4.793756000	3.281416000
C	6.436210000	6.063363000	3.351605000
H	6.798659000	6.656542000	4.199770000
C	7.364103000	4.015265000	4.298705000
C	6.408959000	2.917435000	4.793807000
H	6.081739000	2.273329000	3.965568000
H	6.925424000	2.288552000	5.531606000
H	5.519349000	3.358214000	5.264085000
C	8.563413000	3.382370000	3.572686000
H	8.226846000	2.756826000	2.733208000

H	9.124349000	2.747976000	4.272199000
H	9.231752000	4.160336000	3.179479000
C	7.849267000	4.862723000	5.478331000
H	8.378386000	4.210858000	6.185763000
H	7.009794000	5.331604000	6.011616000
H	8.547145000	5.647815000	5.153567000
N	4.423100000	4.440291000	-0.906394000
C	4.045528000	3.475738000	-1.960380000
C	3.301899000	2.328422000	-1.258160000
H	2.396010000	2.698739000	-0.759654000
H	3.942076000	1.847839000	-0.504328000
H	3.017731000	1.567821000	-1.997584000
C	3.161662000	4.087242000	-3.050937000
H	2.227942000	4.489270000	-2.631857000
H	3.681146000	4.888729000	-3.595115000
H	2.901598000	3.303559000	-3.774380000
C	5.360378000	2.957655000	-2.568826000
H	6.003934000	2.512866000	-1.796070000
H	5.912339000	3.776279000	-3.050291000
H	5.138441000	2.186792000	-3.319291000
C	4.156182000	5.697373000	-0.953559000
H	3.604554000	6.140294000	-1.791528000
C	5.721839000	6.756104000	2.277583000
I	8.543508000	5.307339000	-0.452336000
C	3.729007000	3.398035000	2.197228000
C	2.772789000	4.387516000	2.461928000

H	2.953738000	5.424670000	2.176575000
C	1.572379000	4.068241000	3.094236000
H	0.828349000	4.838461000	3.300305000
C	1.321427000	2.734938000	3.470723000
C	0.096074000	2.400025000	4.120810000
C	2.273066000	1.731369000	3.209447000
H	2.070504000	0.701096000	3.503446000
C	3.465806000	2.073439000	2.574633000
H	4.195287000	1.284310000	2.375879000
N	-0.907216000	2.126922000	4.649562000

Complex **3a-I-eq**

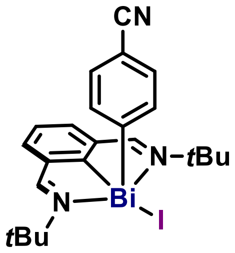

C	5.46741531005775	8.16630861622878	2.25186979225934
H	5.79337921681429	8.77413010490215	3.08681552842080
C	4.78057432810089	8.74584453154485	1.18820360657499
H	4.57590140522635	9.80766055688285	1.19783079544724
C	4.34860422824830	7.96991278007004	0.11568761201858
H	3.80737403278111	8.42533015706907	-0.70424797772912
C	4.61230245656020	6.59566239197602	0.10557064040280
C	5.30521349041490	6.02479238206638	1.16392051499507
Bi	5.72984601504635	3.83747940336972	1.14069151176337
N	6.58233131735434	4.84201451159876	3.29890556603632
C	6.42223550053467	6.10525973694554	3.33526223479551
H	6.78616494012075	6.71042205171821	4.16146482114022

C	7.30080712080421	4.07180888061108	4.33373563109840
C	6.35307885981316	2.96363378248115	4.80338249057456
H	6.06415636790520	2.31850719612480	3.97423019916052
H	6.85243191496377	2.35009020149129	5.55364196171959
H	5.45042617069822	3.38615626219714	5.24628509105479
C	8.52474835817891	3.45182508390031	3.64743764334004
H	8.22421235069555	2.80986166544596	2.81855184558312
H	9.07893180480320	2.84491203532697	4.36383666794957
H	9.18678276432026	4.22959757498101	3.26481254834174
C	7.74337492009328	4.92180805814889	5.52333893012820
H	8.24524369580330	4.27946608403136	6.24641254017617
H	6.89260223130323	5.38880681189041	6.02187429264780
H	8.44547798474732	5.70169473410026	5.22538378360755
N	4.39203627944472	4.45587547730415	-0.90385198079952
C	4.01668283922279	3.48714857131380	-1.95232863222749
C	3.24845143572680	2.35621127455878	-1.26054113391389
H	2.34474954376594	2.73546433473200	-0.78261380380916
H	3.86499110586441	1.87163353804321	-0.50349033487984
H	2.96336474330223	1.60502710191338	-1.99732031628926
C	3.16246959830545	4.09750873733824	-3.06167472472357
H	2.23667274440979	4.51944125843095	-2.66725622069003
H	3.69710171876322	4.87723124700767	-3.60567785874945
H	2.90000166882022	3.31544866404042	-3.77351620983826
C	5.32785838310016	2.94344101613670	-2.53446088268536
H	5.93614581117688	2.47649441072355	-1.75900688560463
H	5.90620513367450	3.74528903155365	-2.99514795115098
H	5.10829897678209	2.19236350402860	-3.29374672070330
C	4.16534219106829	5.70760309391426	-0.97300058531351
H	3.64256594199613	6.15552427420339	-1.81406923612379

C	5.72920010320721	6.79169233337383	2.23985119822331
C	3.75883009044017	3.37254630671880	2.19366624381234
C	2.81894972562522	4.36027101624368	2.48239484651390
H	3.00179166367764	5.39139571952860	2.21592055613299
C	1.62812564887424	4.03829753683616	3.11724469151015
H	0.89901506186016	4.80482742414812	3.34067126064244
C	1.37273098211337	2.70897596084422	3.46934095897305
C	0.15115663064501	2.37142555415063	4.12408468850479
C	2.30916862619061	1.71013873577049	3.18242172021716
H	2.10479954468810	0.68436841105630	3.45657220568401
C	3.49410737397734	2.04936073484293	2.54670369573803
H	4.21273828380742	1.27078898182550	2.32583518789512
N	-0.83626801665282	2.09946142322346	4.65409181639407
I	6.70074701373294	-0.06458734890870	1.04660462775342

Complex **3a-BF$_4$**

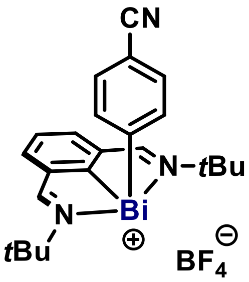

C	5.300920000	8.193300000	2.348856000
H	5.591618000	8.775817000	3.225540000
C	4.549099000	8.782059000	1.326928000
H	4.257564000	9.829657000	1.405810000
C	4.157933000	8.036263000	0.210091000
H	3.559217000	8.496974000	-0.578401000
C	4.533259000	6.683550000	0.110232000
C	5.298176000	6.104690000	1.123023000
Bi	5.892477000	3.960575000	0.963413000
N	6.683593000	4.890166000	3.179249000
C	6.421379000	6.143660000	3.296389000
H	6.733563000	6.719242000	4.175837000
C	7.430898000	4.118880000	4.198205000
C	6.516311000	2.960245000	4.628061000
H	6.249408000	2.328600000	3.771449000
H	7.040450000	2.336432000	5.364953000
H	5.593280000	3.345407000	5.082861000
C	8.687564000	3.576024000	3.497785000
H	8.416772000	2.949506000	2.637186000
H	9.261196000	2.958723000	4.202619000
H	9.324200000	4.402828000	3.153499000

C	7.827544000	4.953374000	5.419483000
H	8.367120000	4.306984000	6.123949000
H	6.945481000	5.356900000	5.937427000
H	8.492103000	5.785839000	5.146731000
N	4.444761000	4.583104000	-1.016584000
C	4.078804000	3.661565000	-2.115796000
C	3.309467000	2.496655000	-1.472964000
H	2.392117000	2.859035000	-0.989250000
H	3.927478000	1.985554000	-0.724363000
H	3.037632000	1.767469000	-2.248291000
C	3.222387000	4.320990000	-3.200727000
H	2.275909000	4.703390000	-2.791978000
H	3.753887000	5.145014000	-3.698271000
H	2.983079000	3.566544000	-3.961377000
C	5.401157000	3.157545000	-2.718731000
H	6.009868000	2.647584000	-1.959802000
H	5.978209000	3.994428000	-3.135896000
H	5.186812000	2.440763000	-3.523202000
C	4.125664000	5.828848000	-1.006488000
H	3.540546000	6.280767000	-1.816341000
C	5.675358000	6.840377000	2.246448000
C	4.005500000	3.283353000	2.046351000
C	3.083984000	4.197134000	2.570683000
H	3.236691000	5.270971000	2.458878000
C	1.952517000	3.745708000	3.247400000
H	1.231790000	4.453057000	3.658319000

C	1.743043000	2.362233000	3.399876000
C	0.587568000	1.893424000	4.094356000
C	2.666178000	1.439674000	2.873403000
H	2.496008000	0.369917000	2.996333000
C	3.792994000	1.907316000	2.200117000
H	4.506877000	1.188595000	1.796153000
N	-0.357078000	1.512420000	4.662388000
B	7.205044000	0.544949000	0.498813000
F	7.484660000	-0.838976000	0.492492000
F	8.241329000	1.253516000	-0.155718000
F	7.091402000	1.004730000	1.841957000
F	5.970622000	0.788313000	-0.167940000

11. NMR spectra

Bismuthinidene **1**

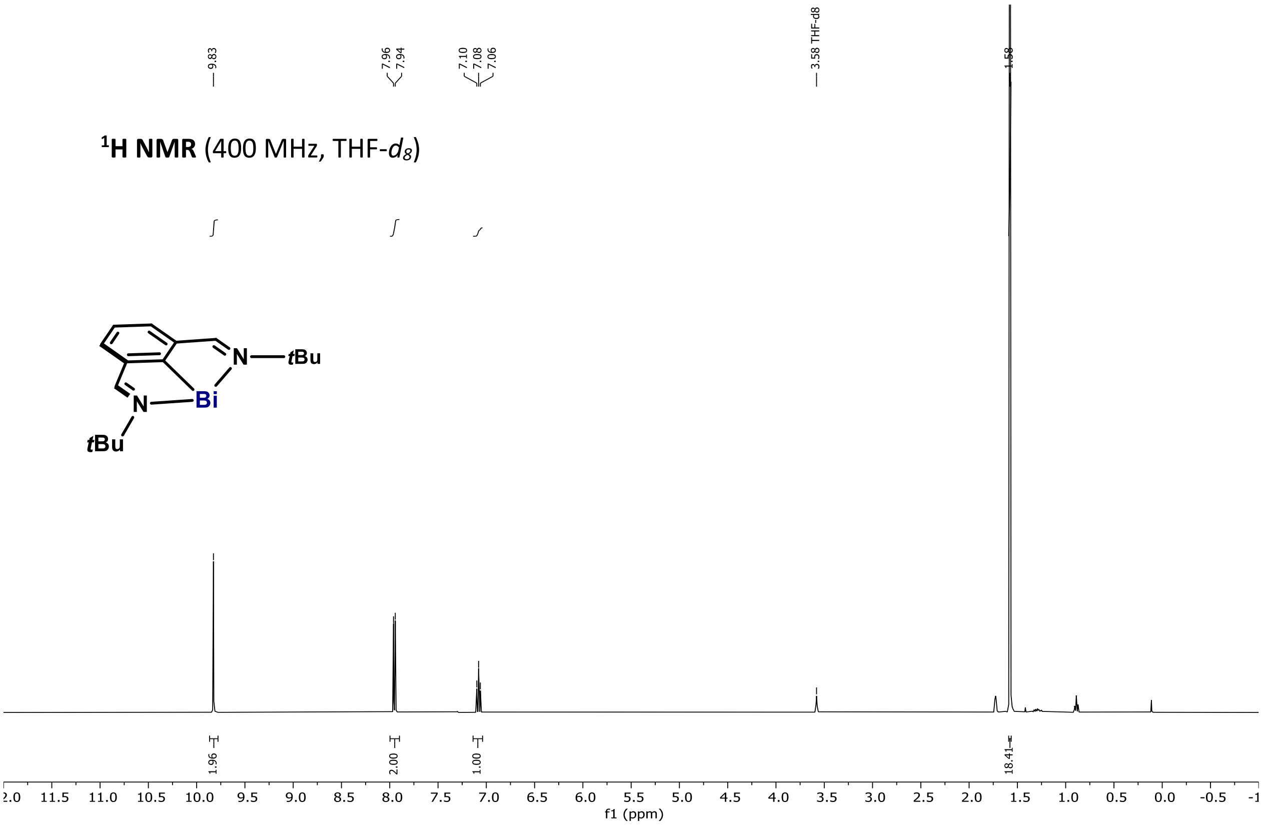

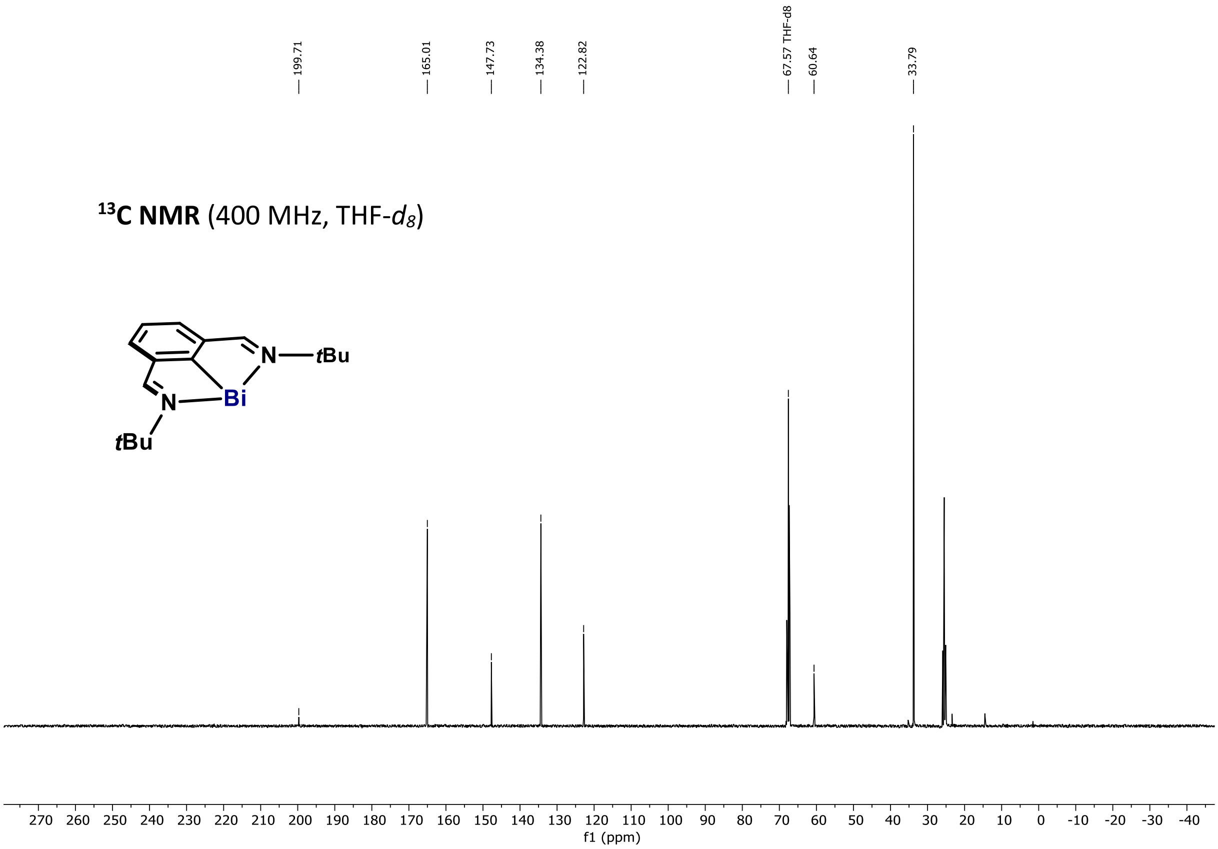

[(2,6-(*t*BuNCH)$_2$C$_6$H$_3$)Bi(4-cyanophenyl)(iodide)] (3a-I)

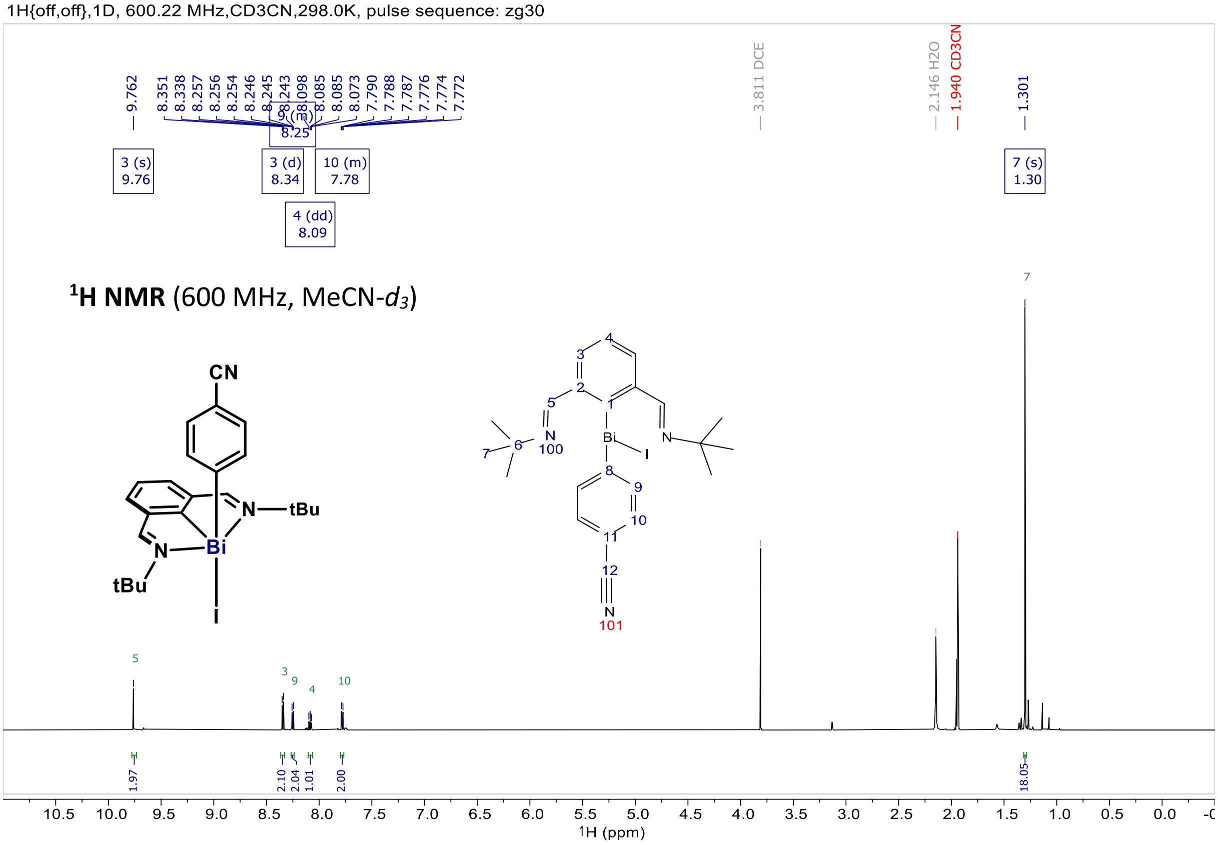

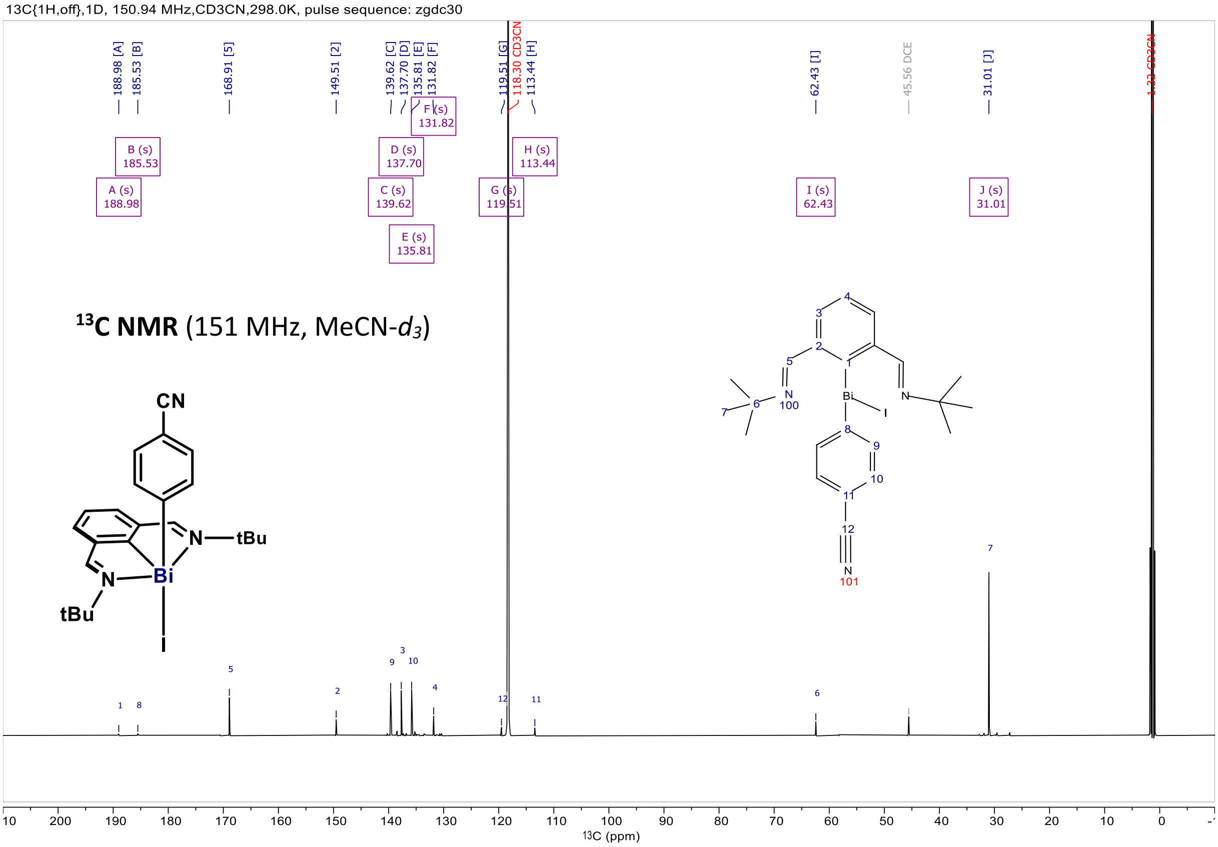

^{1}H-^{13}C-edited HSQC

1H{13C,off},HSQC-EDITED, 600.22 MHz,CD3CN,298.0K, pulse sequence: hsqcedetgpsisp2.3

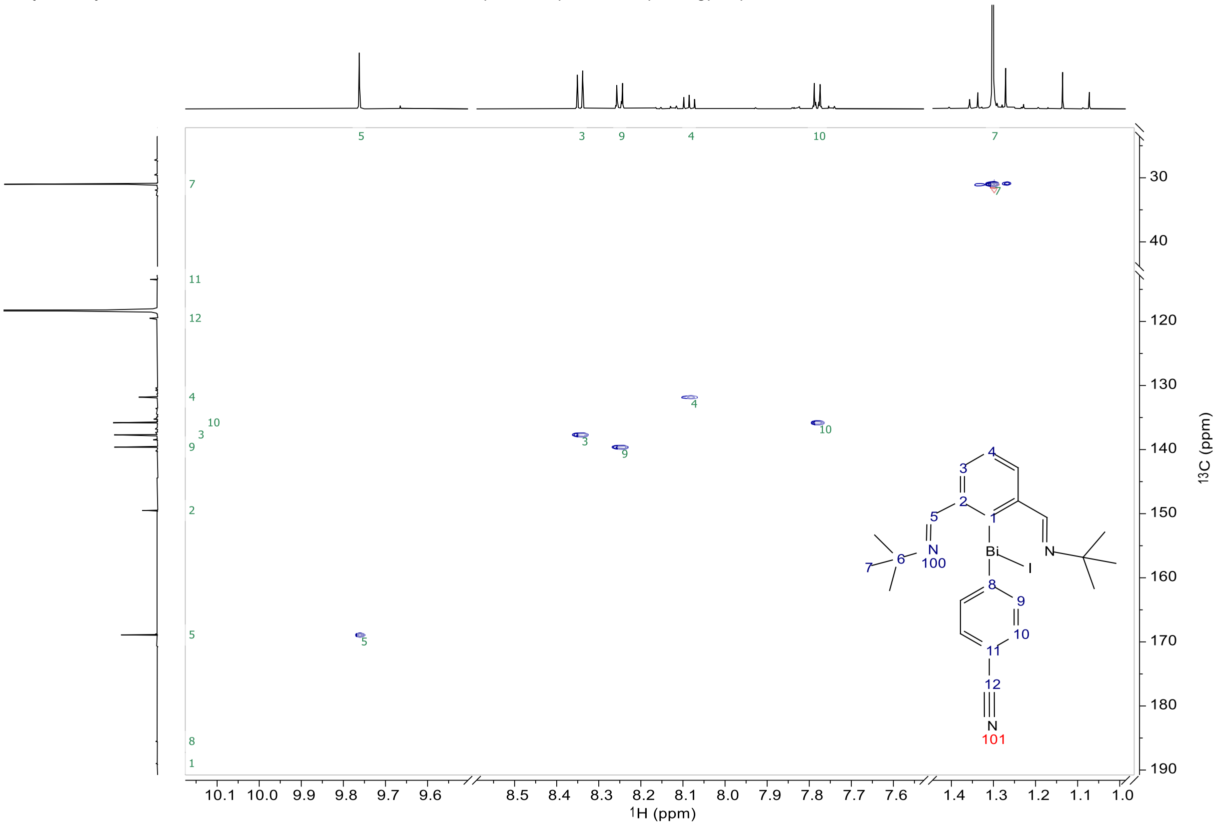

^{1}H-^{13}C HMBC

1H{13C,off},HMBC, 600.22 MHz,CD3CN,298.0K, pulse sequence: hmbcetgpl3nd

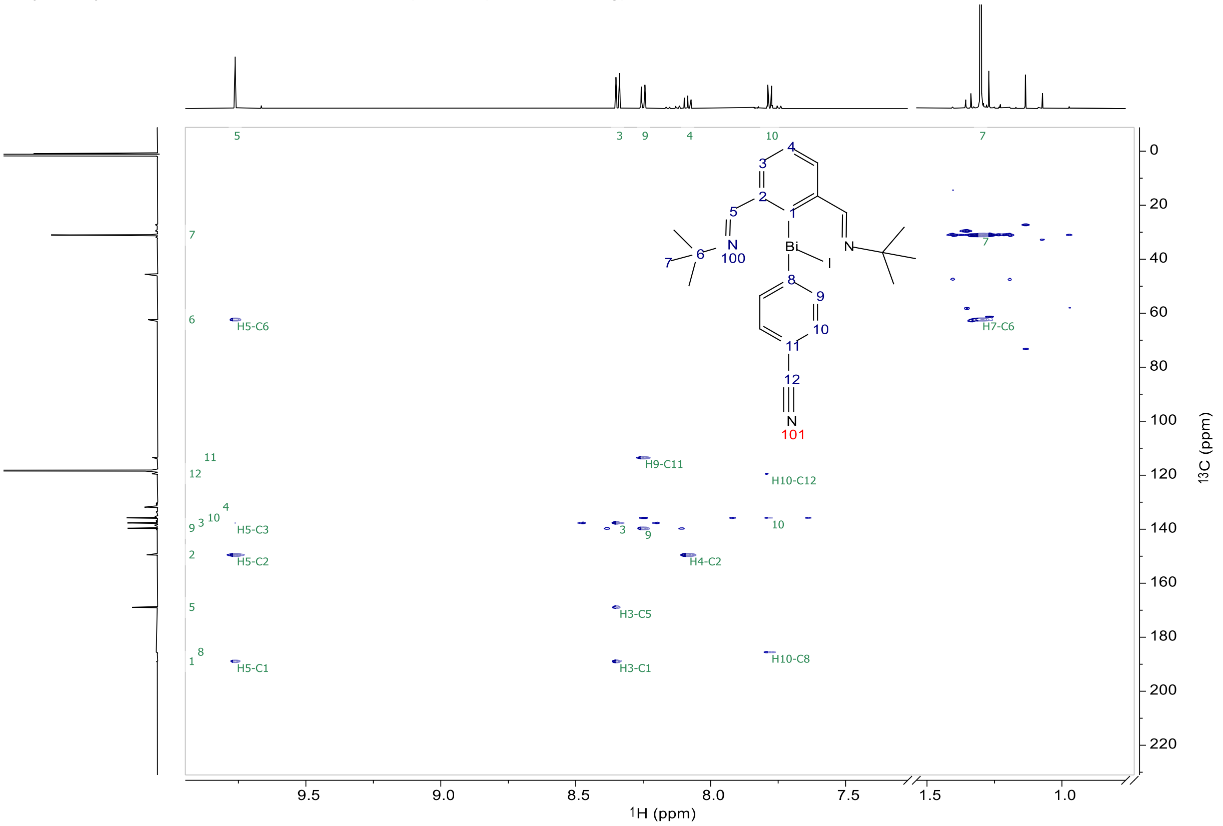

^{1}H-^{1}H COSY

1H{off,off},COSY, 600.22 MHz,CD3CN,298.0K, pulse sequence: cosygpppqf

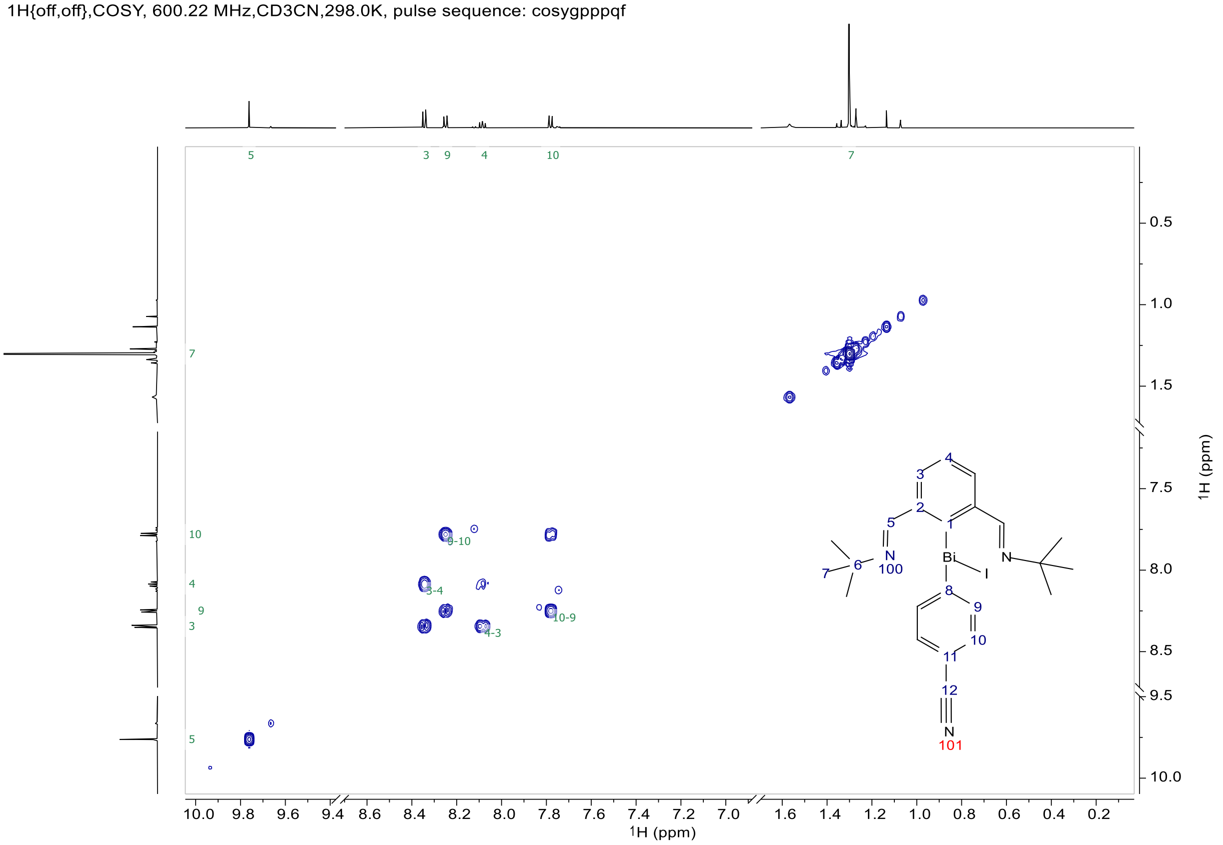

^{1}H-^{1}H NOESY

1H{off,off},NOESY, 600.22 MHz,CD3CN,298.0K, pulse sequence: noesygpph

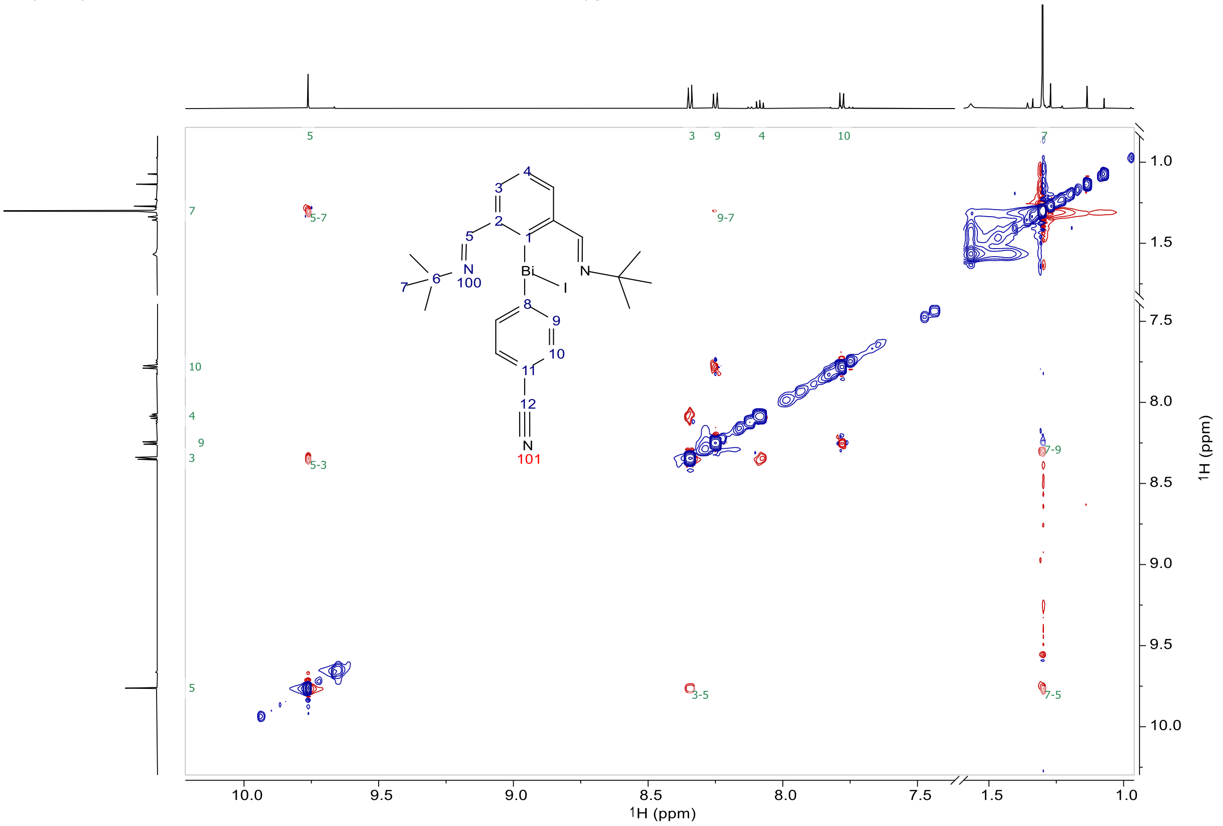

^{1}H-^{15}N HMBC

1H{off,15N},HMBC, 600.22 MHz,CD3CN,298.0K, pulse sequence: hmbcf3gpndqf

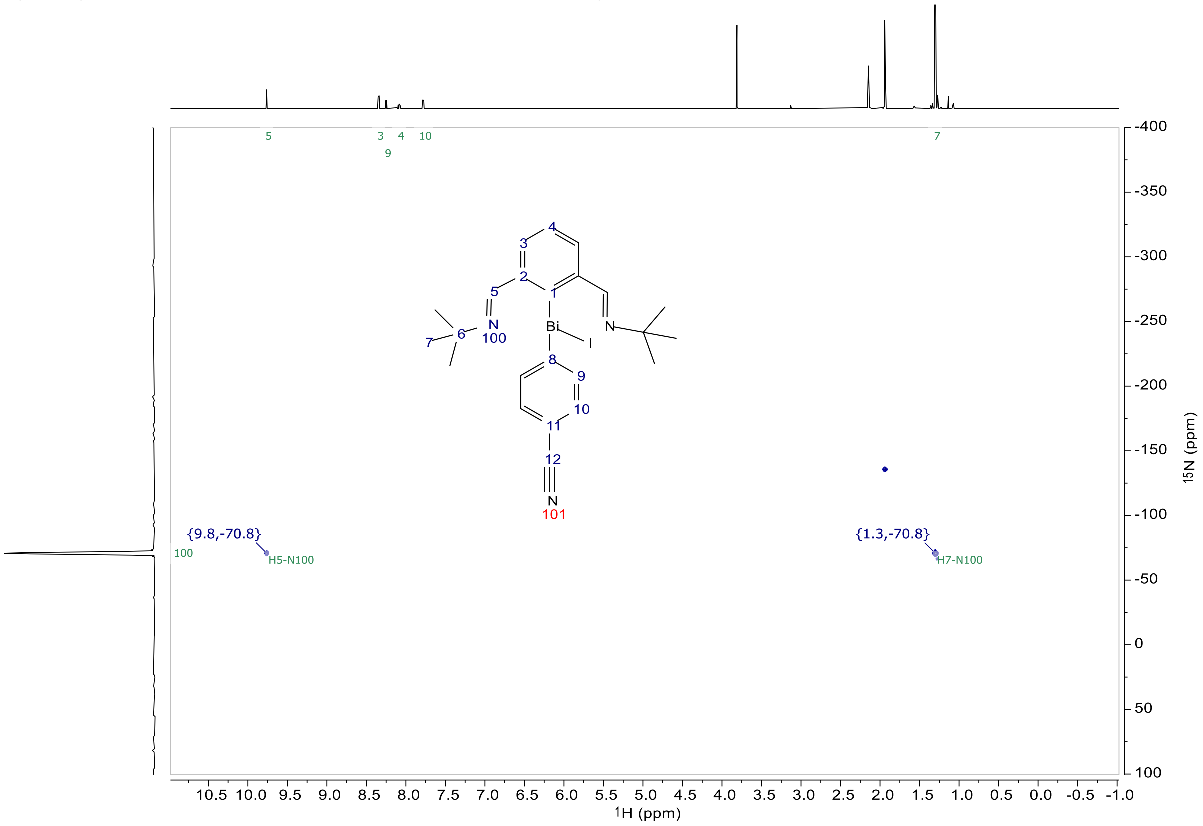

[(2,6-(*t*BuNCH)$_2$C$_6$H$_3$)Bi(4-benzonitrile)(tetrafluoroborate)] (3a-BF4)

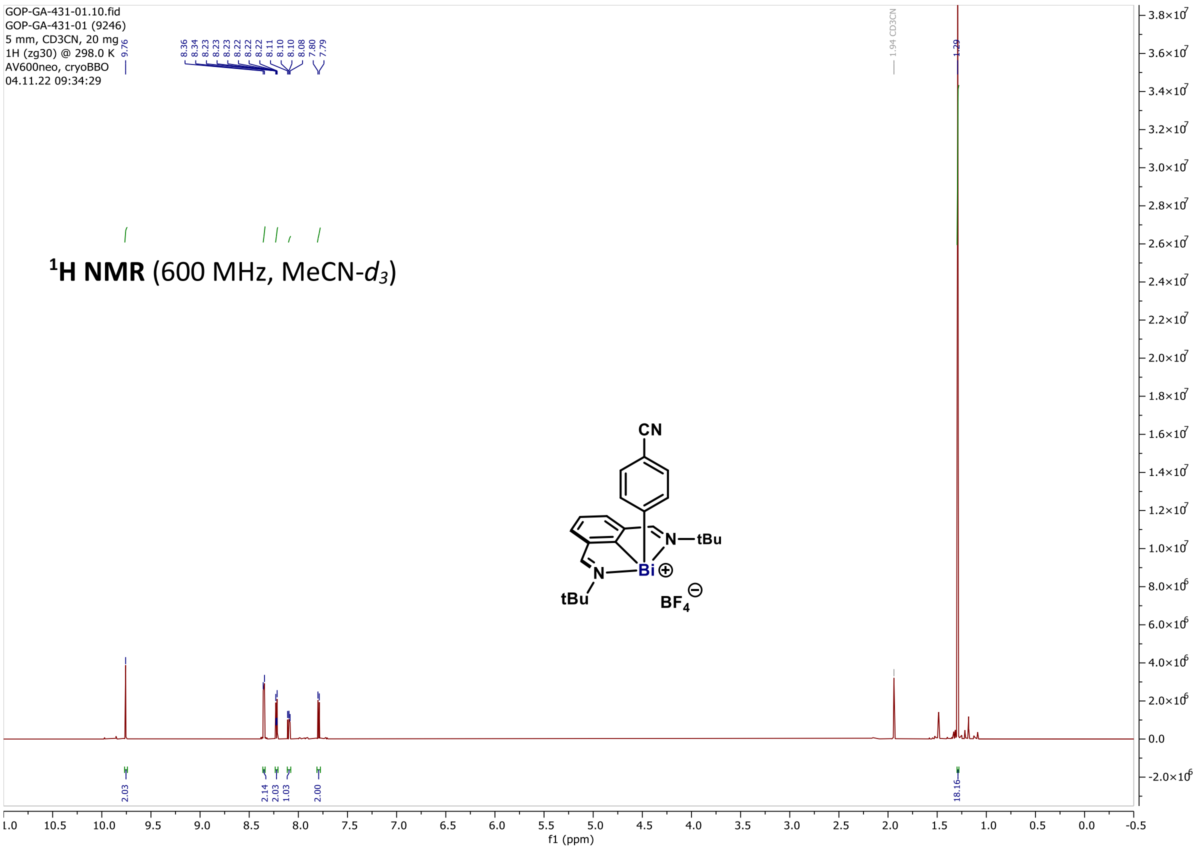

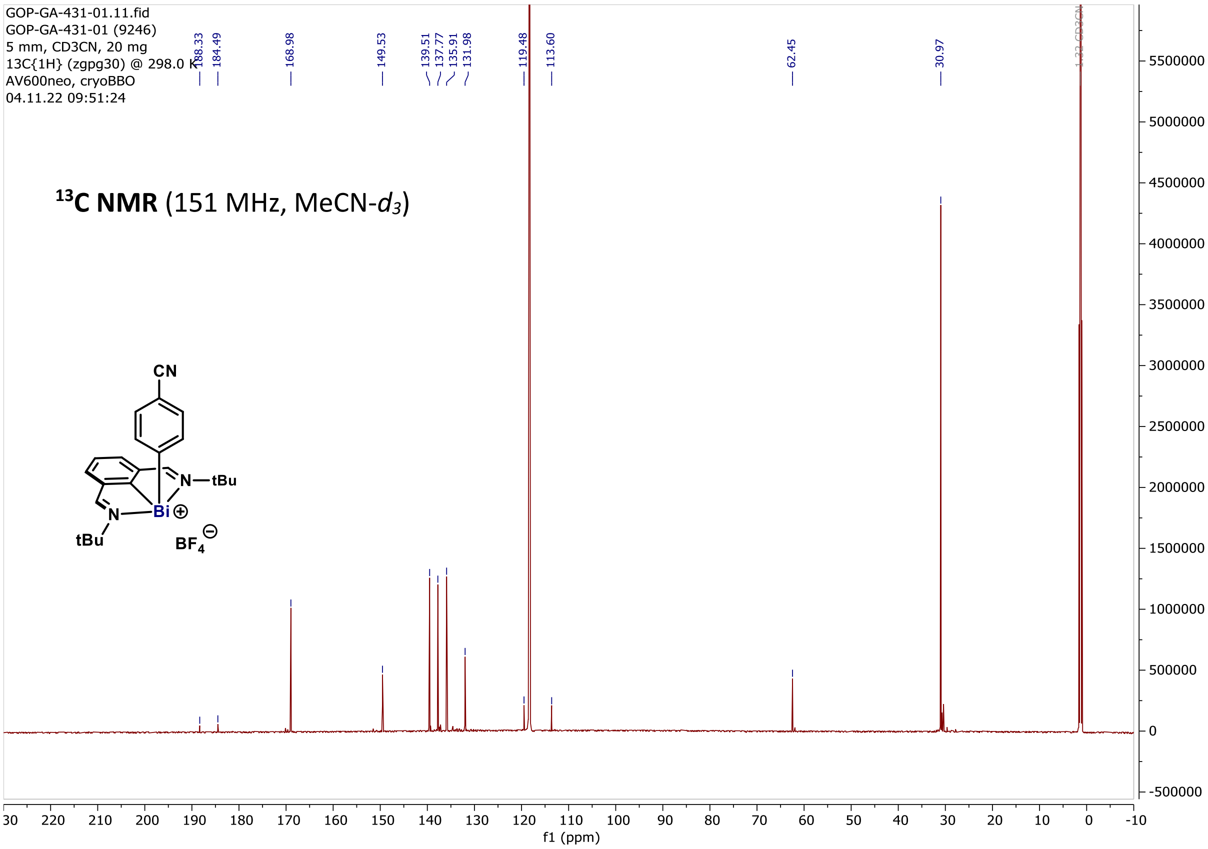

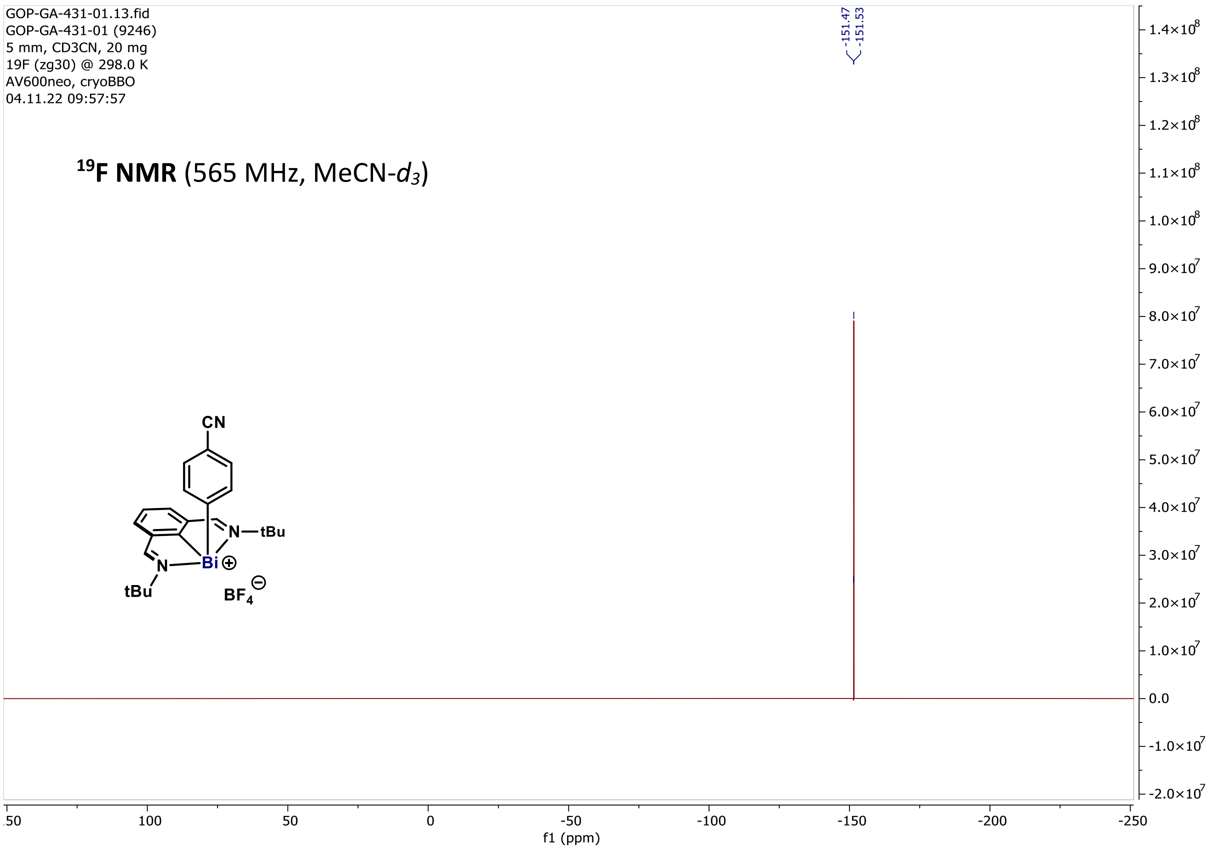
GOP-GA-431-01.13.fid
GOP-GA-431-01 (9246)
5 mm, CD3CN, 20 mg
19F (zg30) @ 298.0 K
AV600neo, cryoBBO
04.11.22 09:57:57
19F NMR (565 MHz, MeCN-d3)
-151.47
-151.53
CN
N—tBu
N
Bi
tBu
BF4
f1 (ppm)

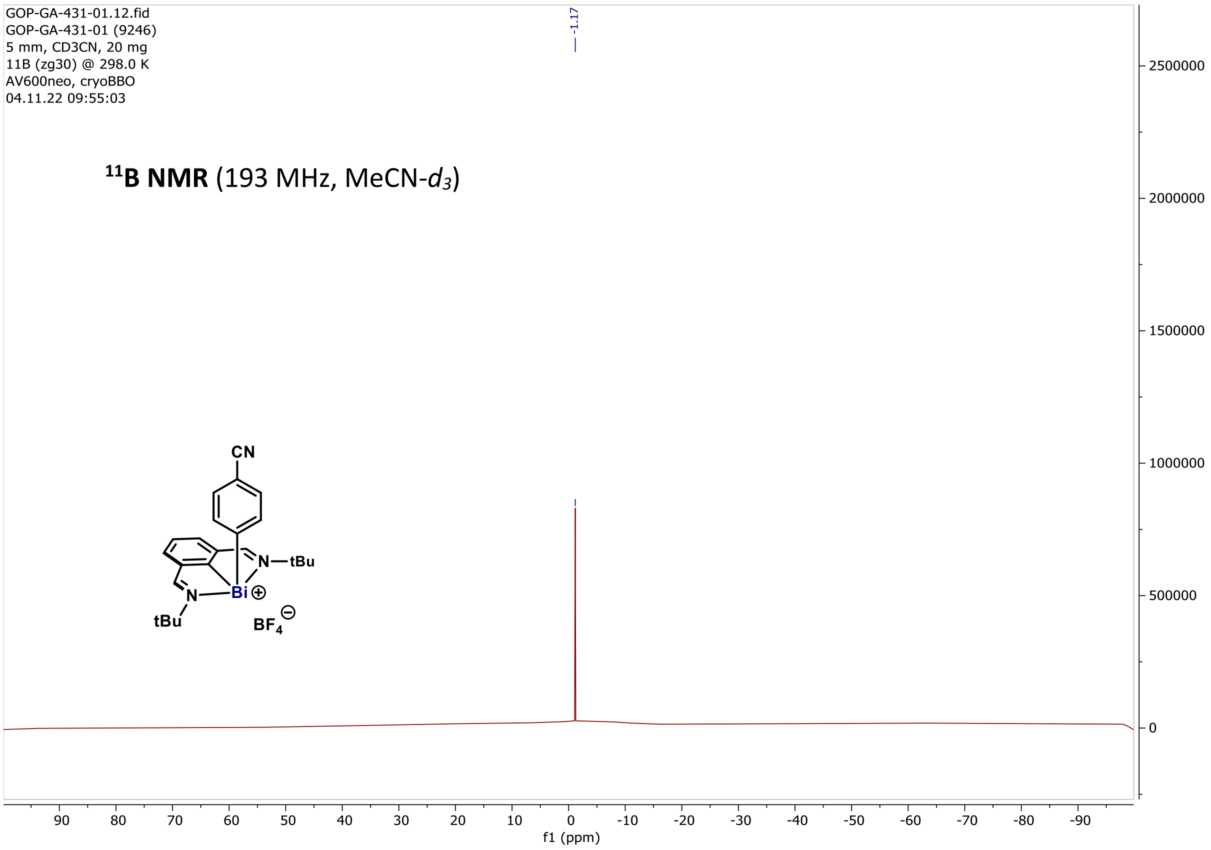
GOP-GA-431-01.12.fid
GOP-GA-431-01 (9246)
5 mm, CD3CN, 20 mg
11B (zg30) @ 298.0 K
AV600neo, cryoBBO
04.11.22 09:55:03
11B NMR (193 MHz, MeCN-d3)
-1.17
CN
N—tBu
N
Bi
tBu
BF4
f1 (ppm)

[(2,6-(*t*BuNCH)$_2$C$_6$H$_3$)BiI$_2$] (1ab)

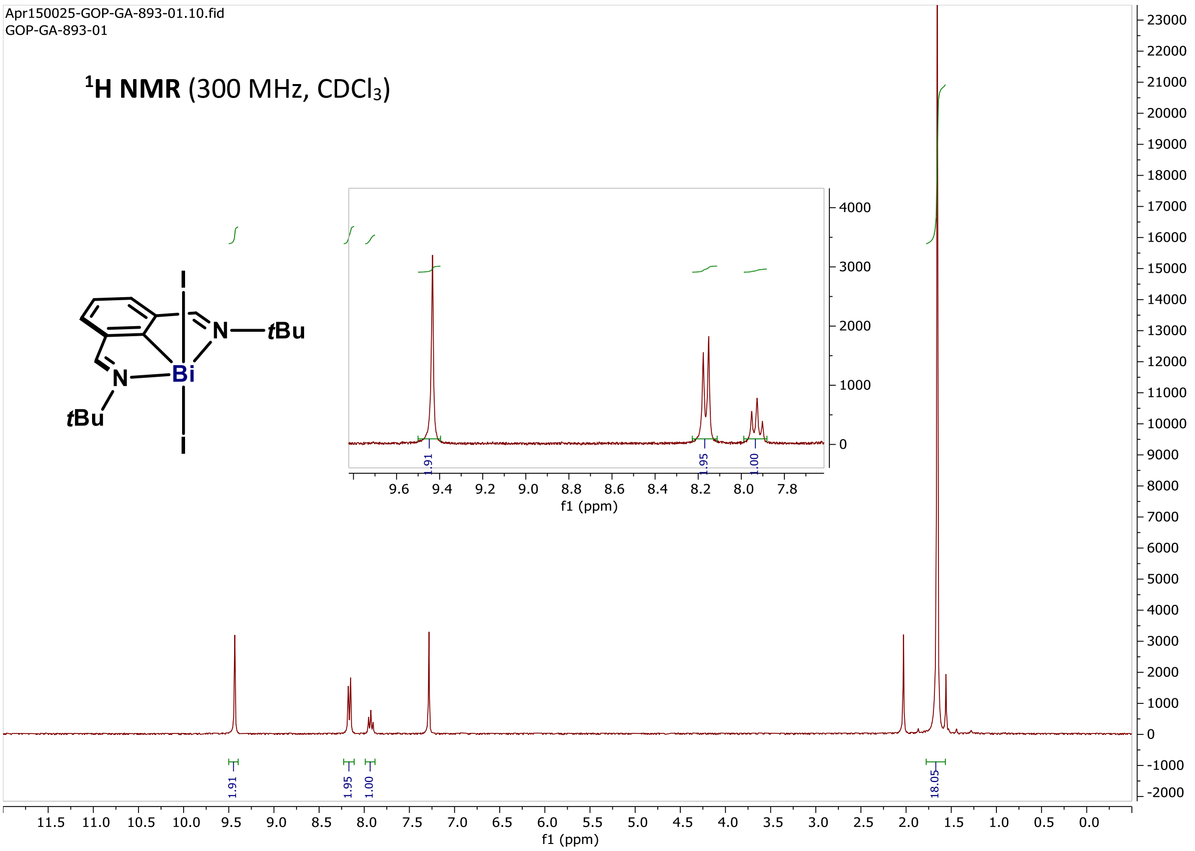

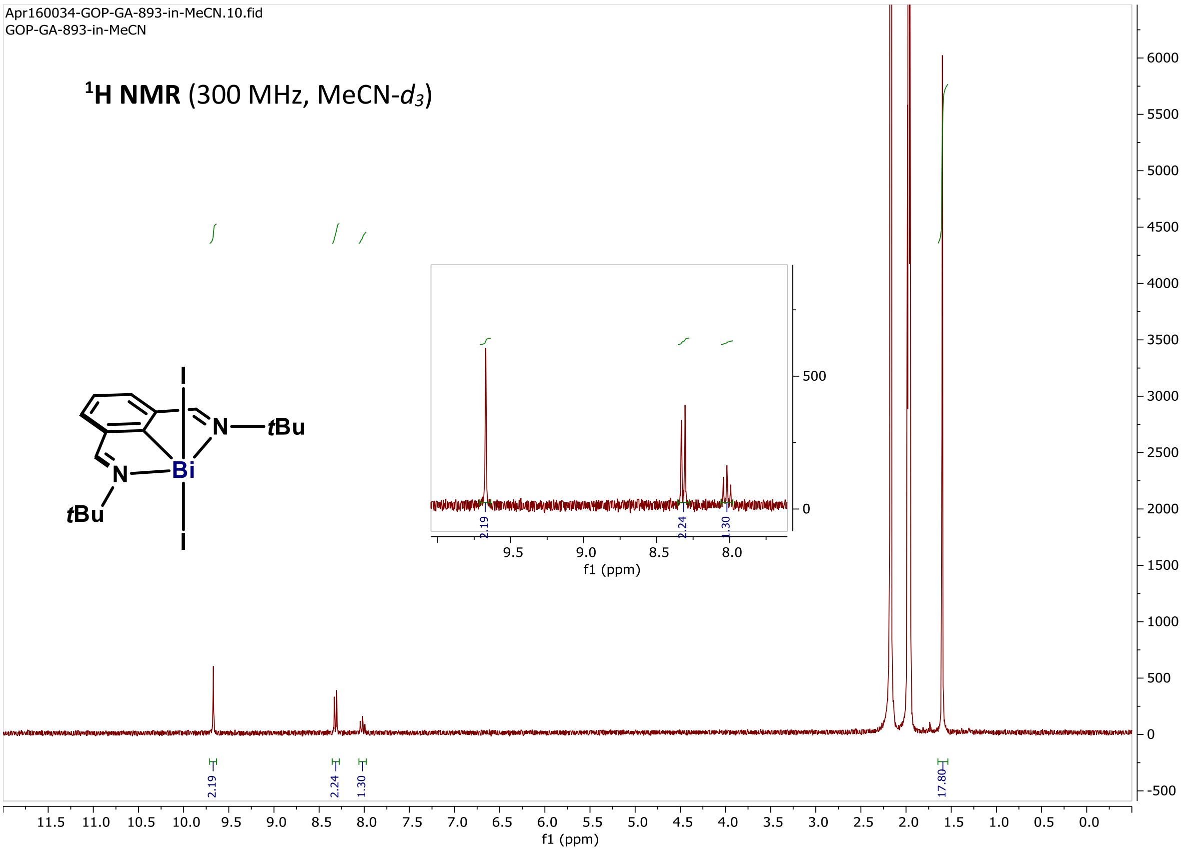

4-(1-Methyl-1*H*-pyrrol-2-yl)benzonitrile (5a)

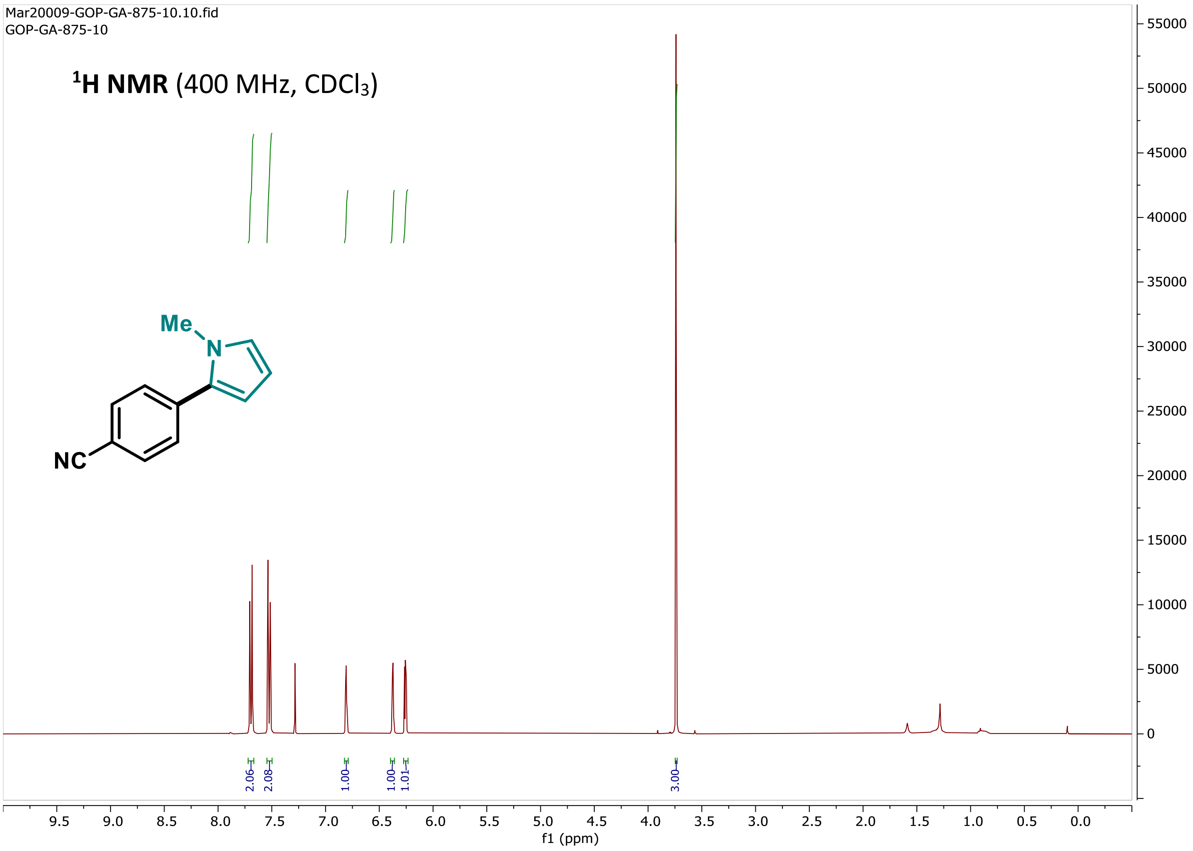

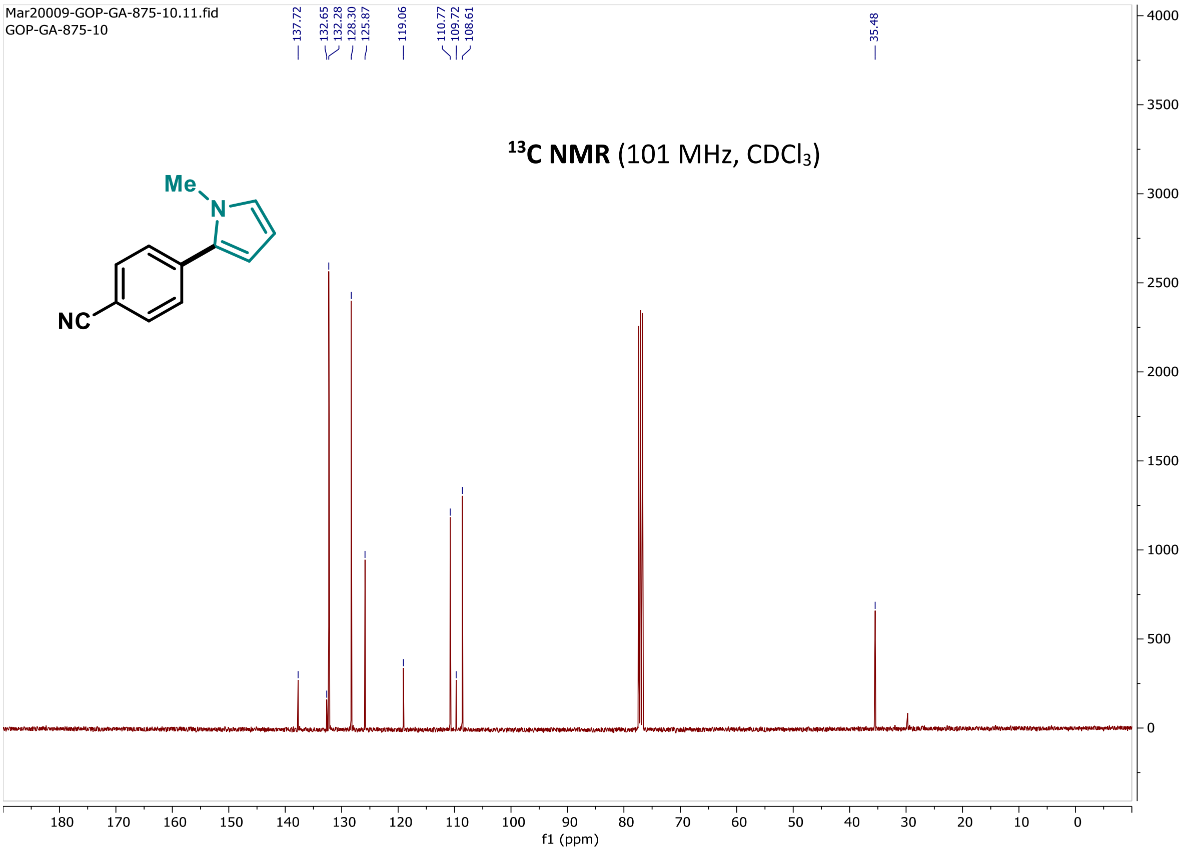

3-(1-Methyl-1*H*-pyrrol-2-yl)benzonitrile (5b)

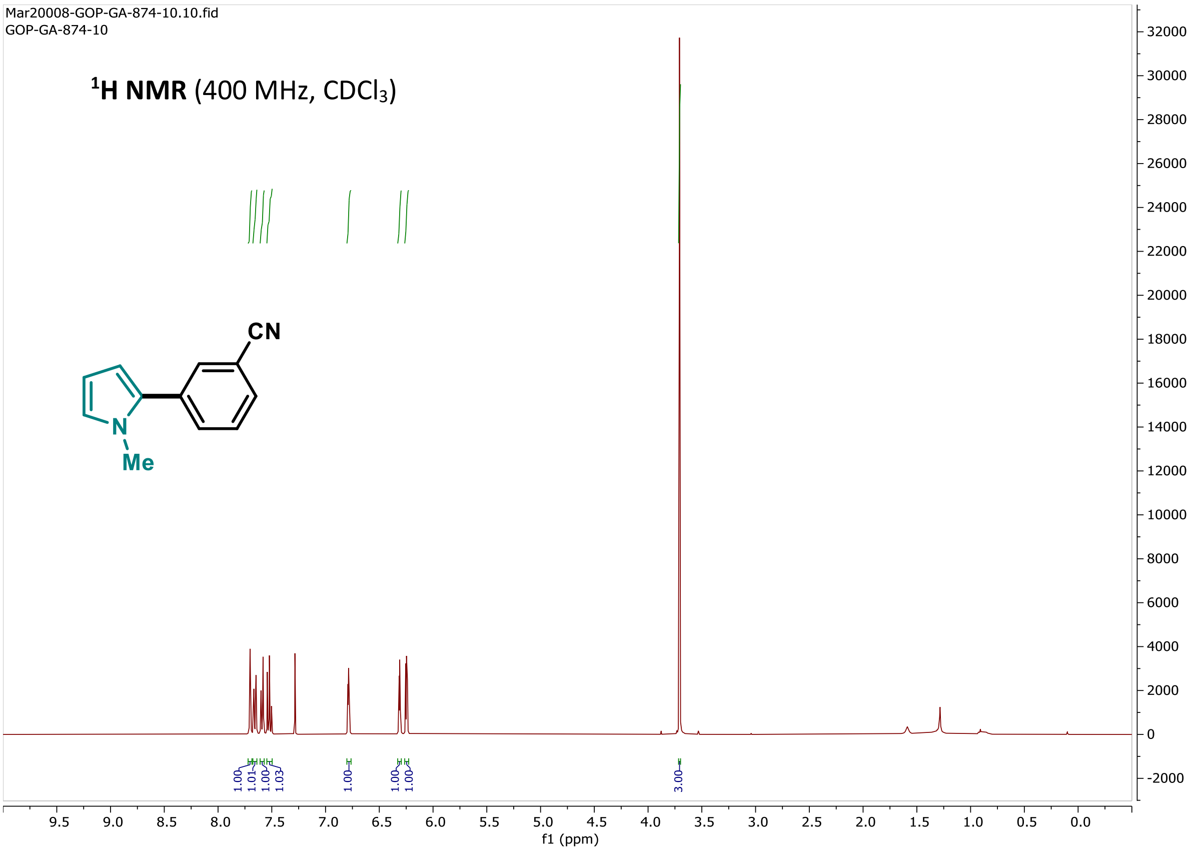

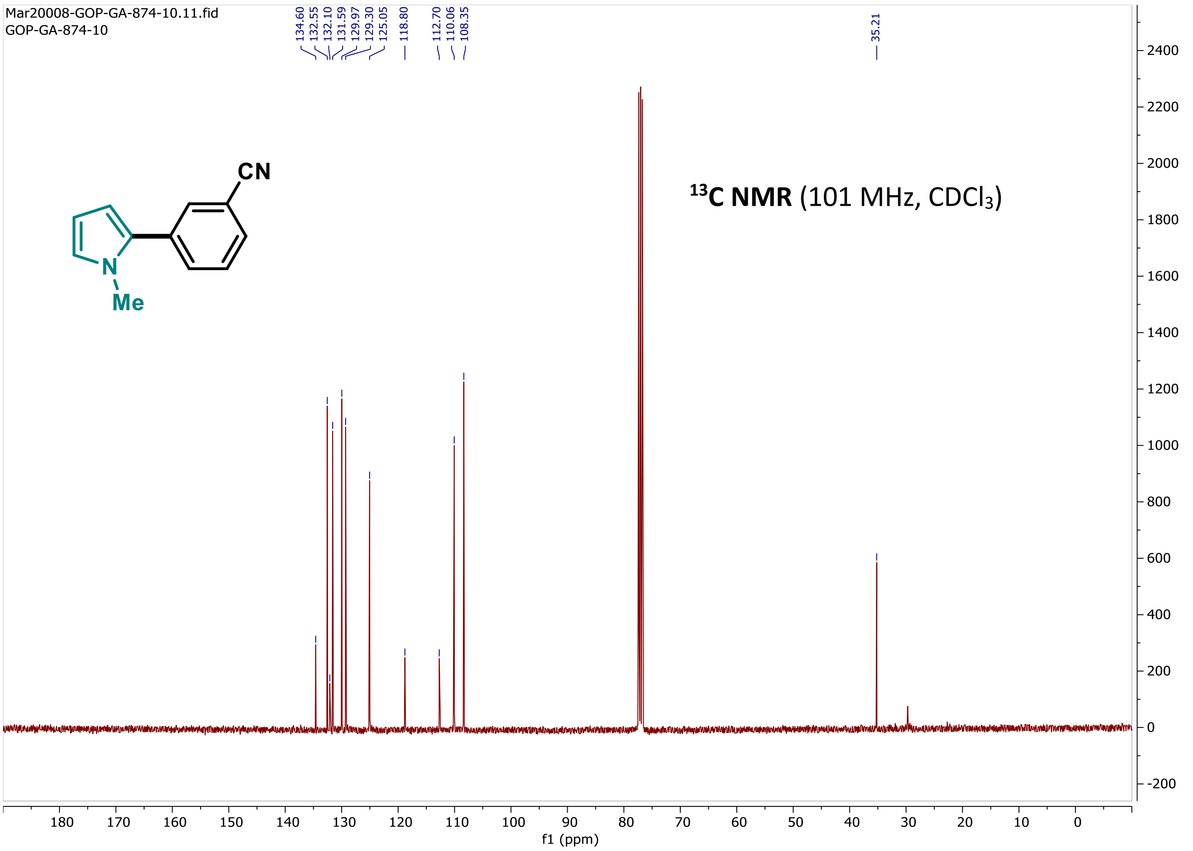

2-(1-Methyl-1*H*-pyrrol-2-yl)benzonitrile (5c)

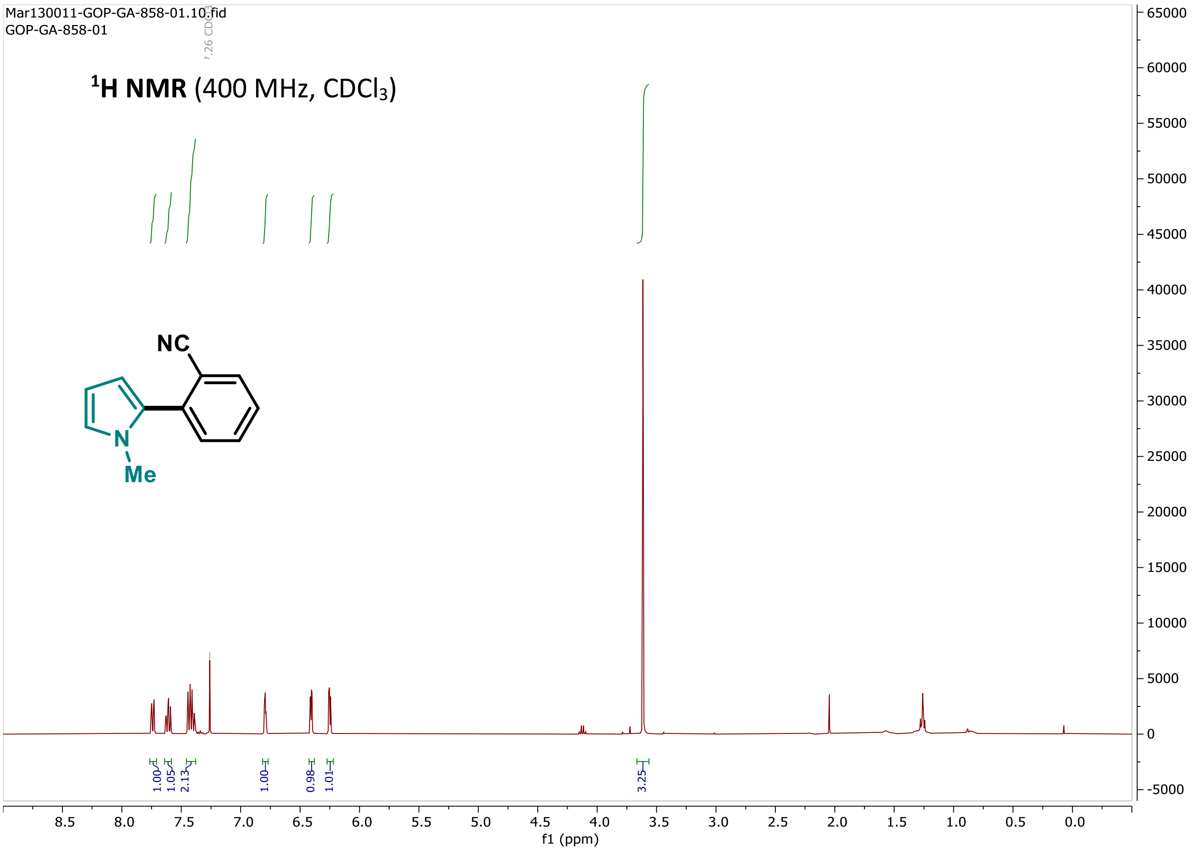

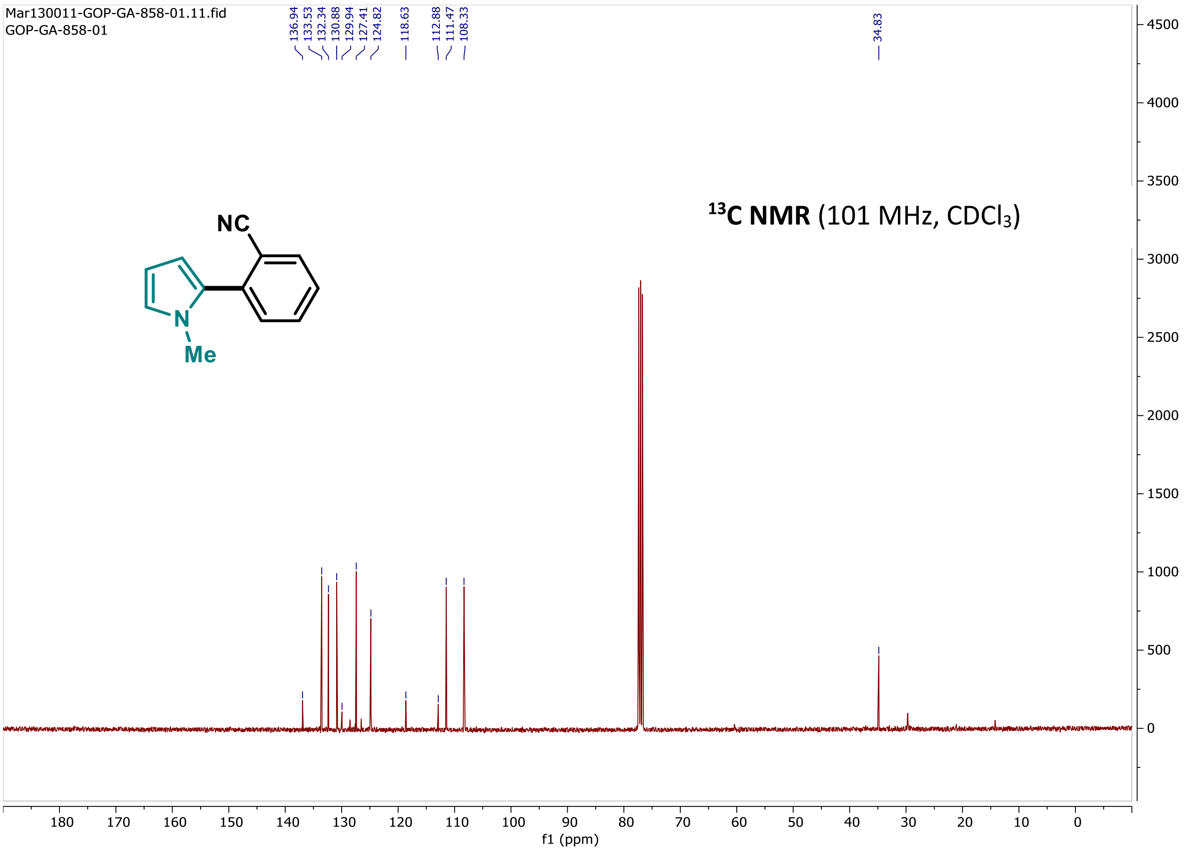

4-(1-Methyl-1*H*-pyrrol-2-yl)phenyl trifluoromethanesulfonate (5d)

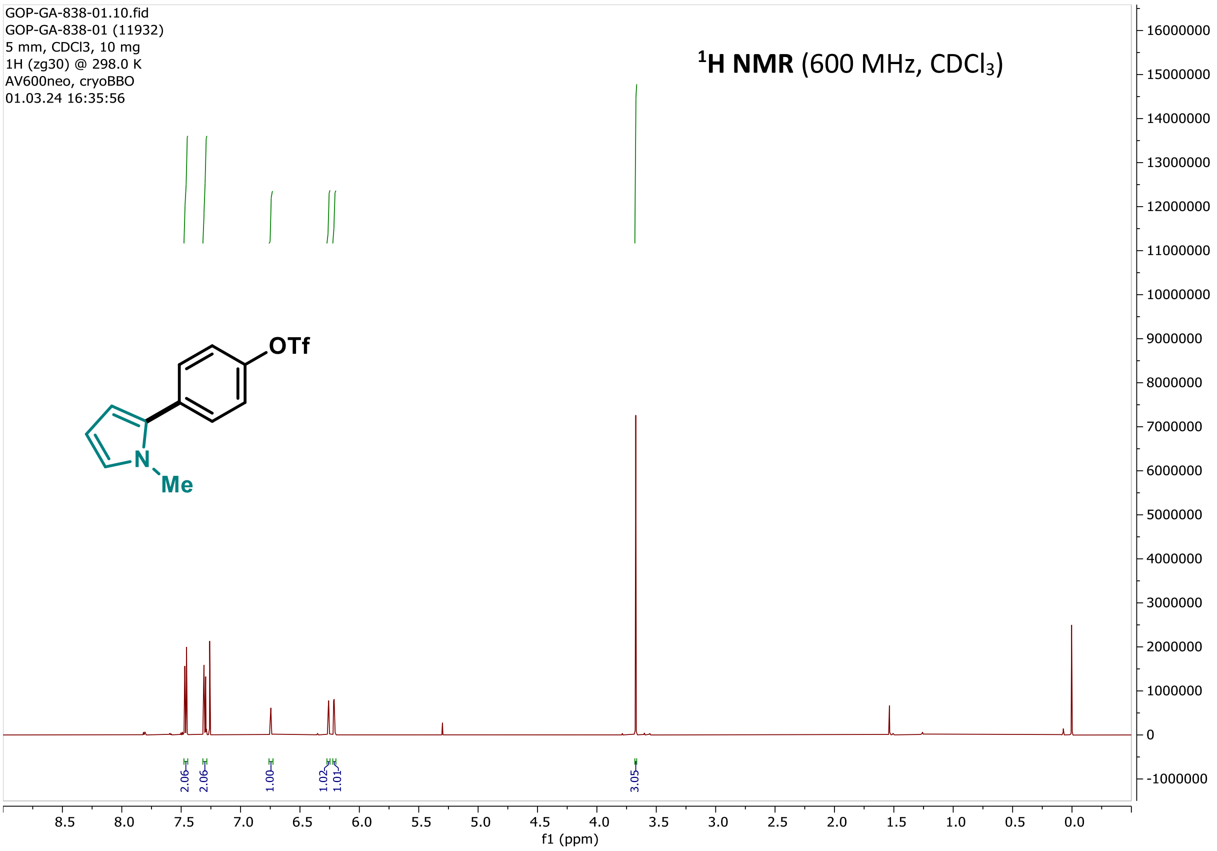

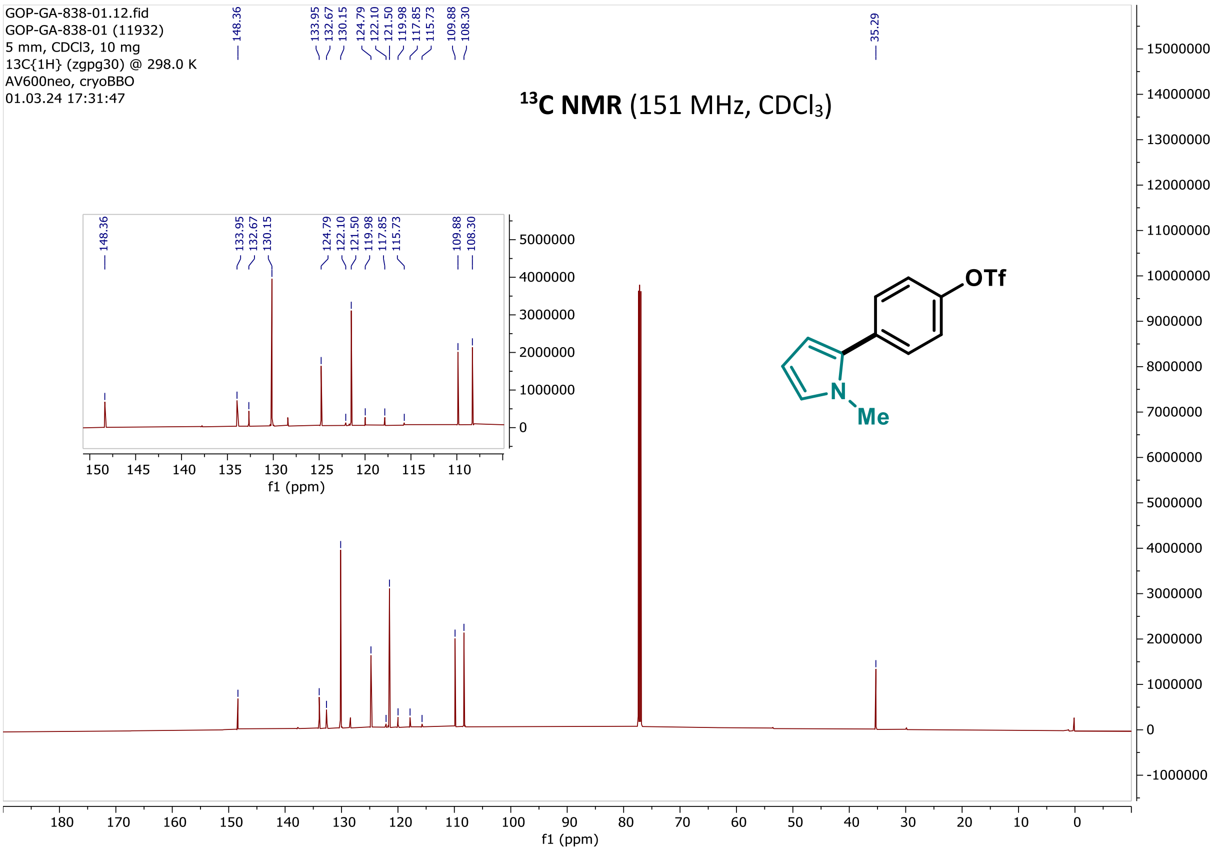

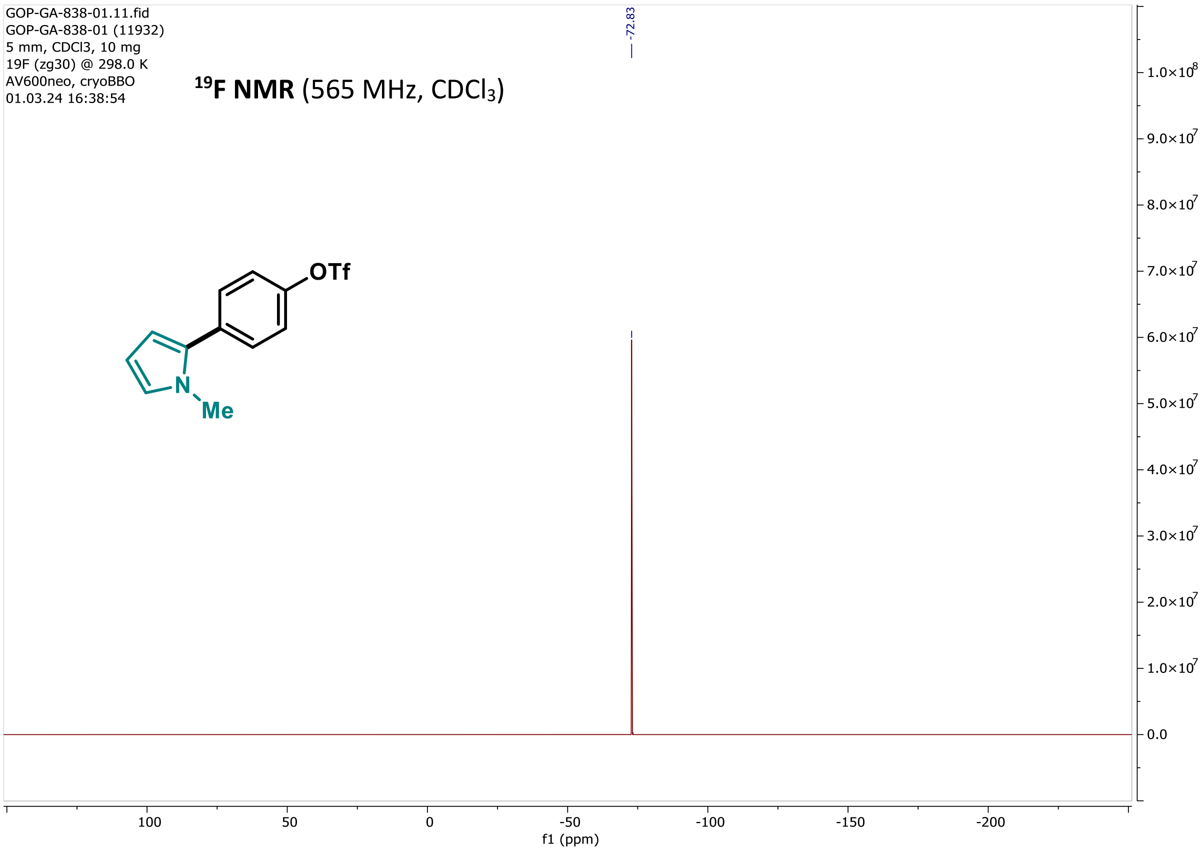
GOP-GA-838-01.11.fid
GOP-GA-838-01 (11932)
5 mm, CDCl3, 10 mg
19F (zg30) @ 298.0 K
AV600neo, cryoBBO
01.03.24 16:38:54
19F NMR (565 MHz, CDCl3)
-72.83
OTf
N
Me
f1 (ppm)

2-(4-Bromophenyl)-1-methyl-1*H*-pyrrole (5e)

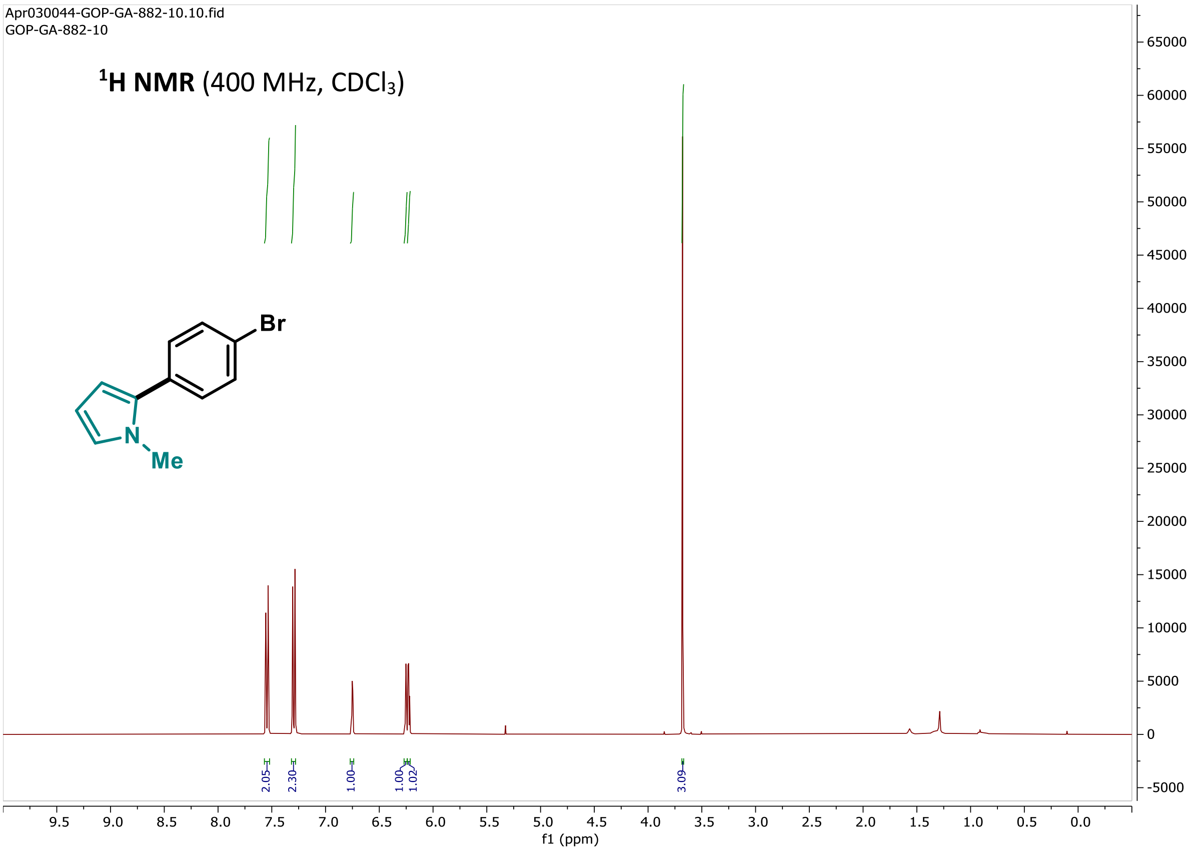

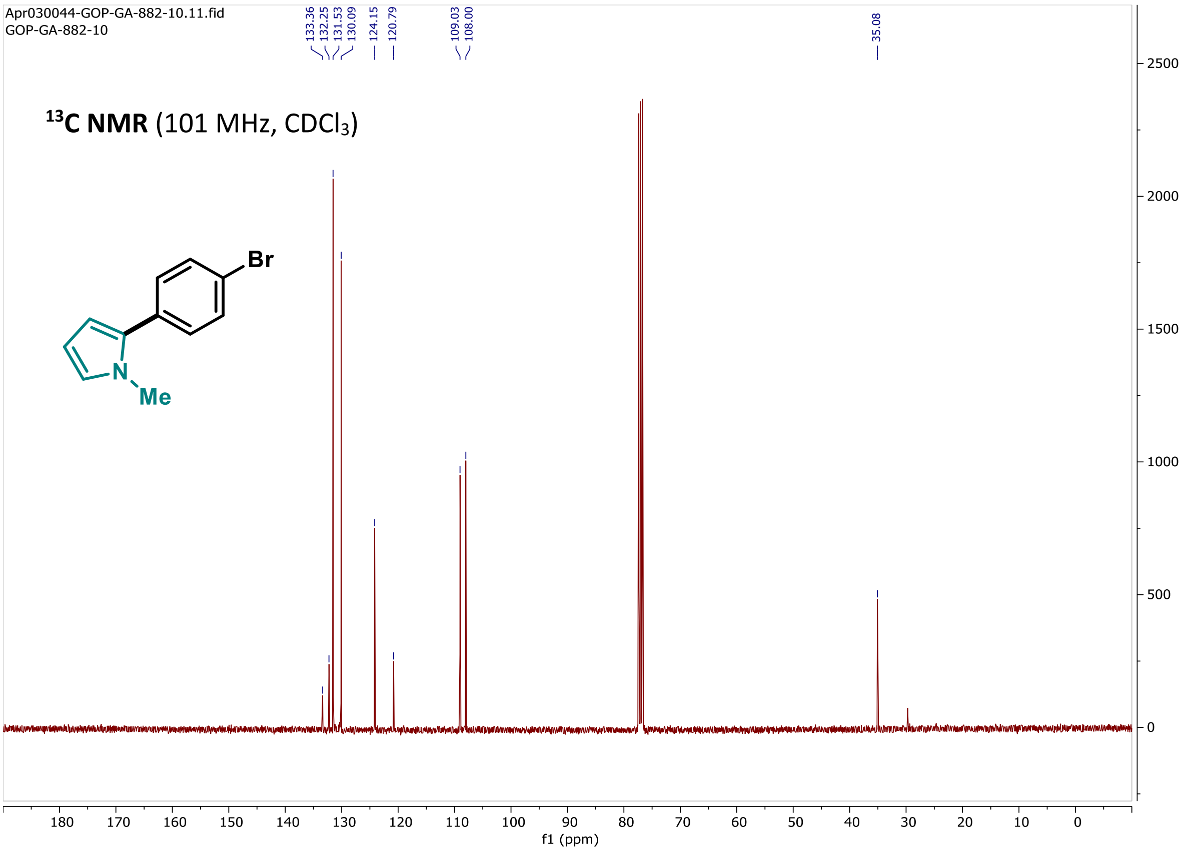

2-(4-Chlorophenyl)-1-methyl-1*H*-pyrrole (5f)

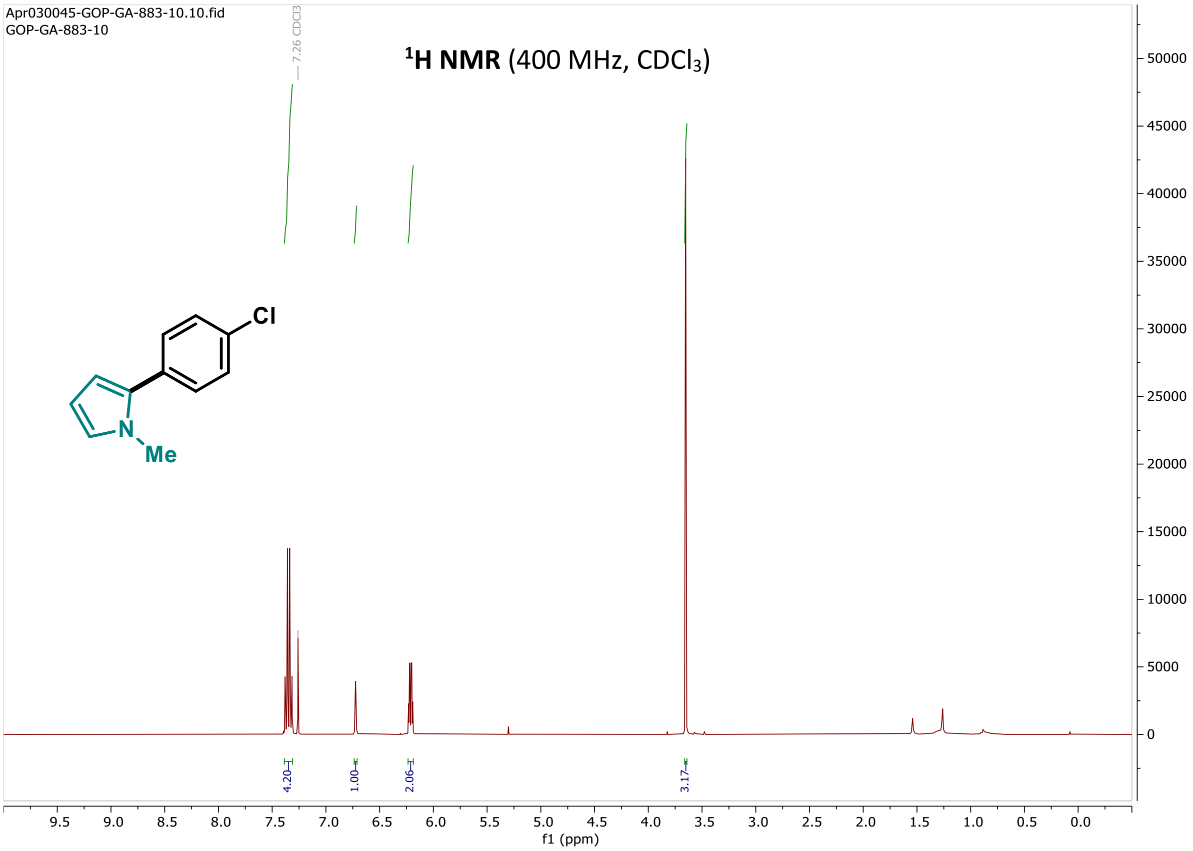

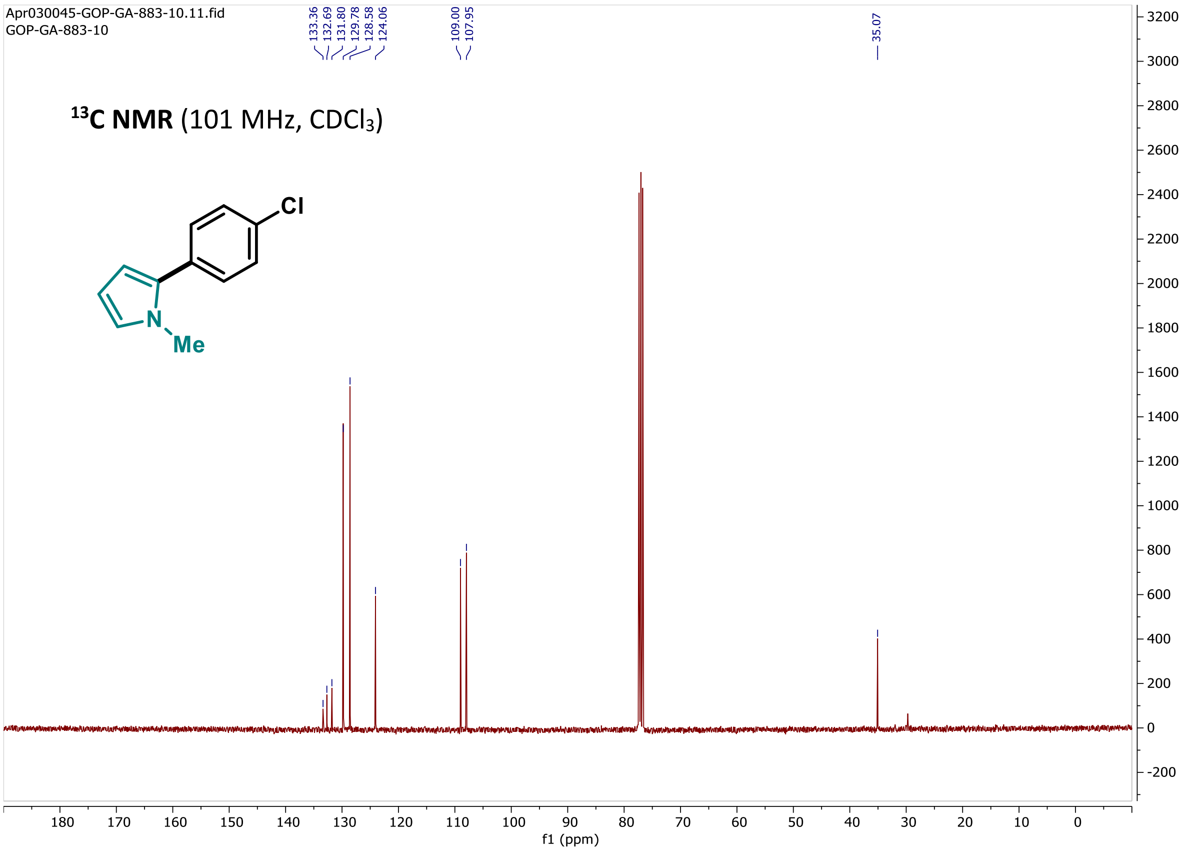

2-(3-bromo-5-(trifluoromethyl)phenyl)-1-methyl-1*H*-pyrrole (5g)

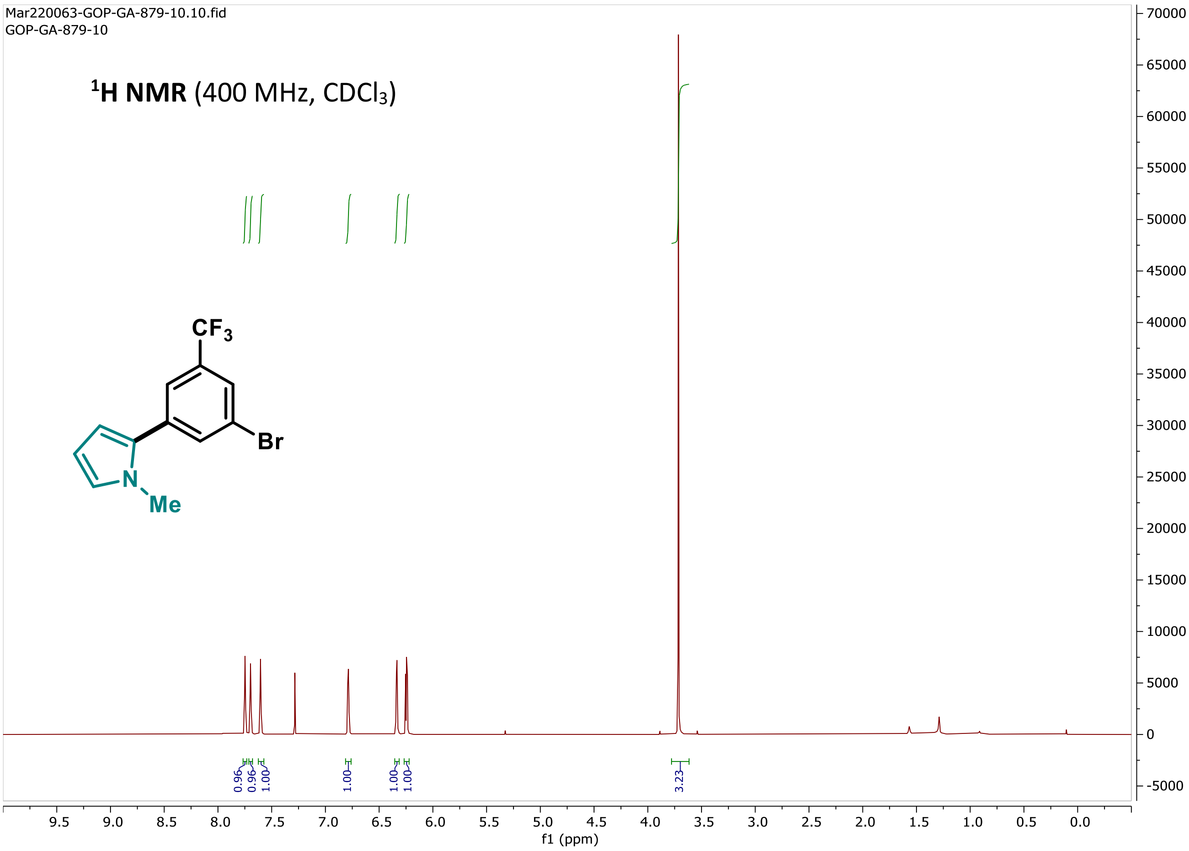

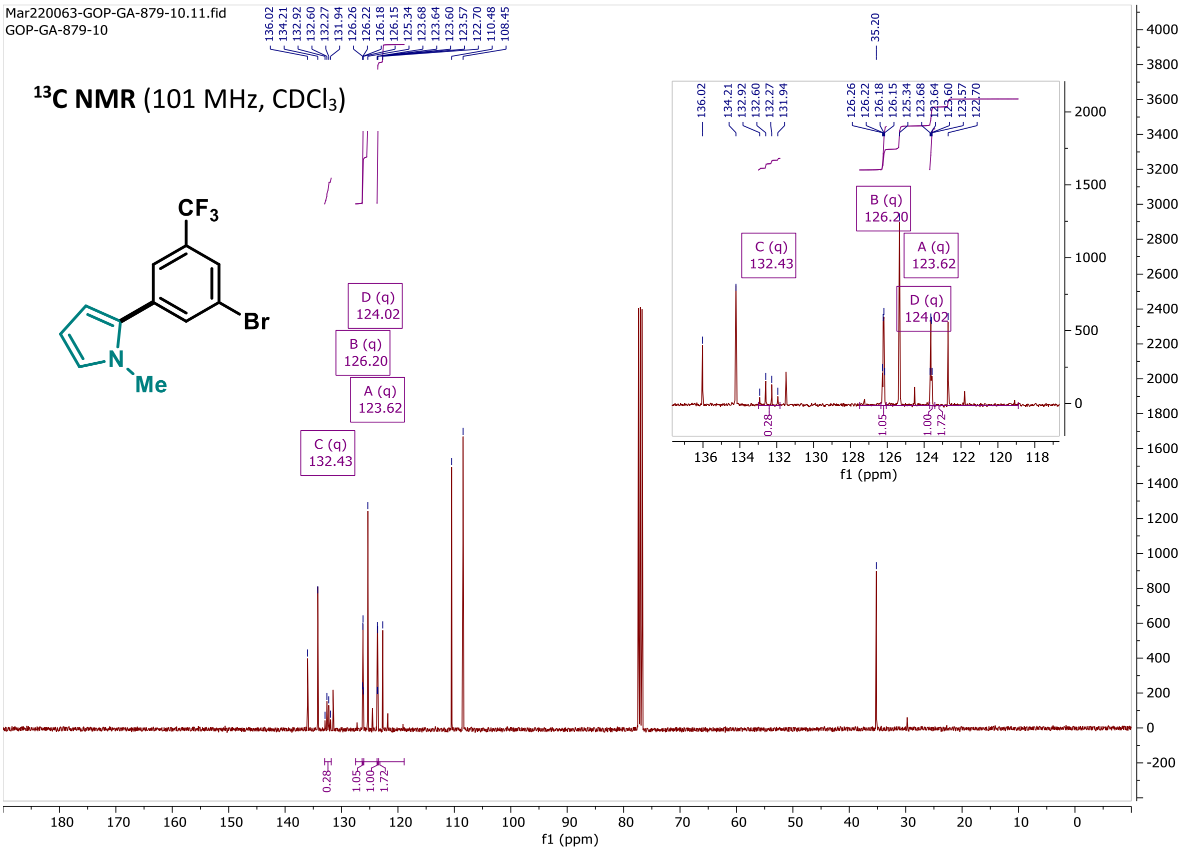

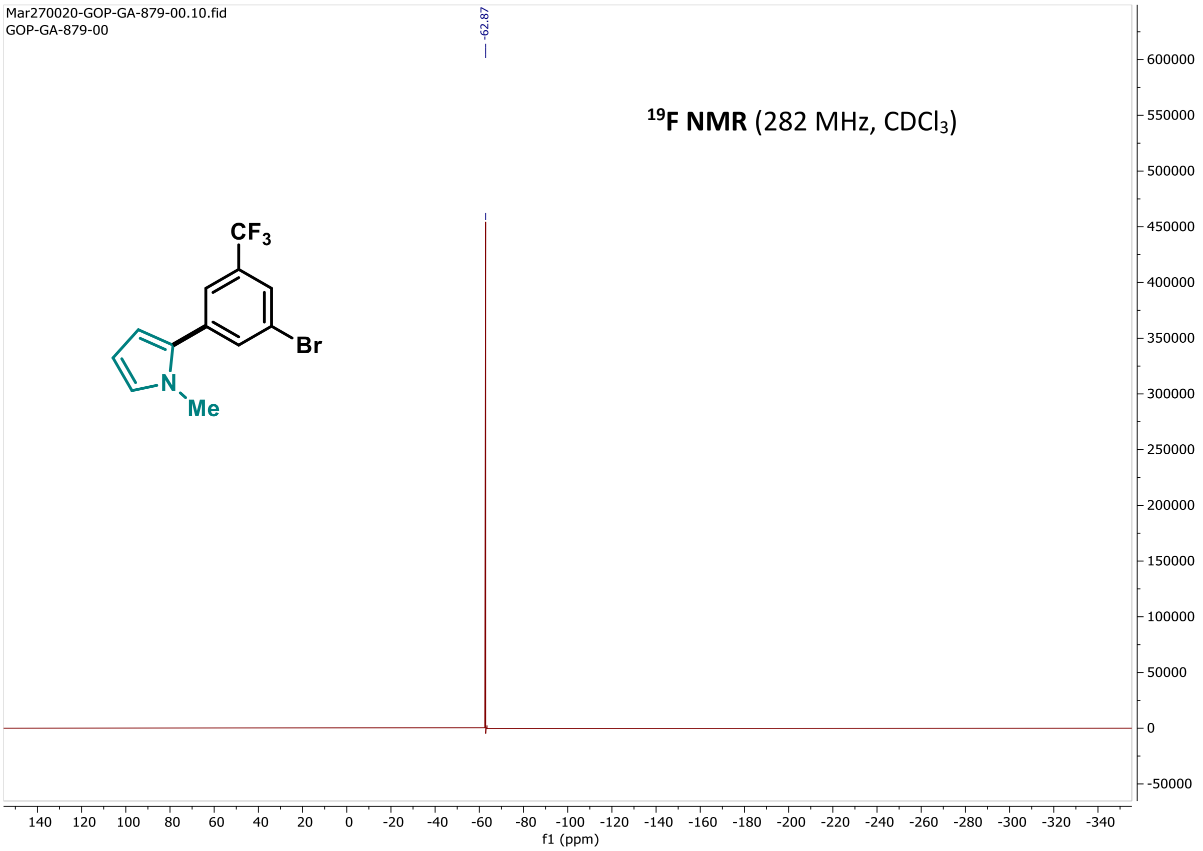

Mar270020-GOP-GA-879-00.10.fid
GOP-GA-879-00
-62.87
19F NMR (282 MHz, CDCl3)
CF3
Br
N
Me
f1 (ppm)

4-(1-Methyl-1*H*-pyrrol-2-yl)-2-(trifluoromethyl)pyridine (5h)

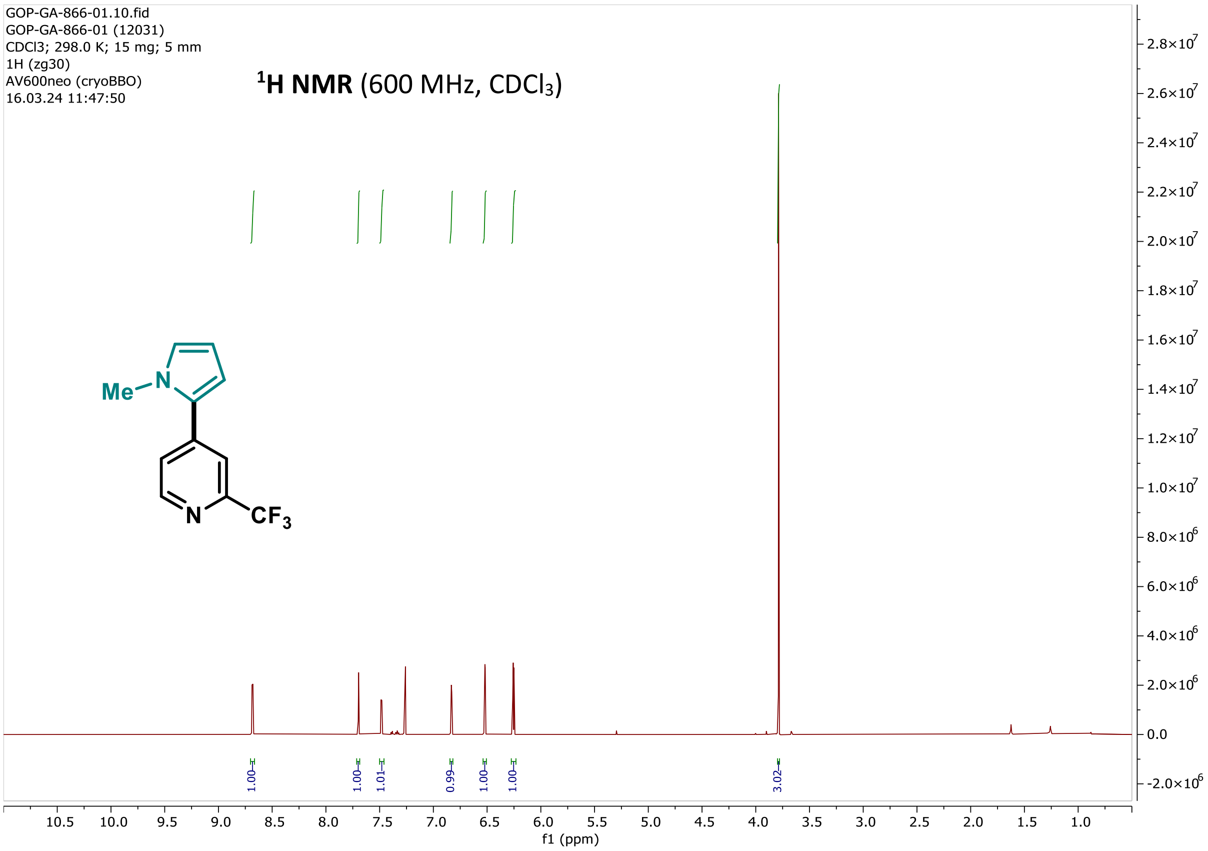

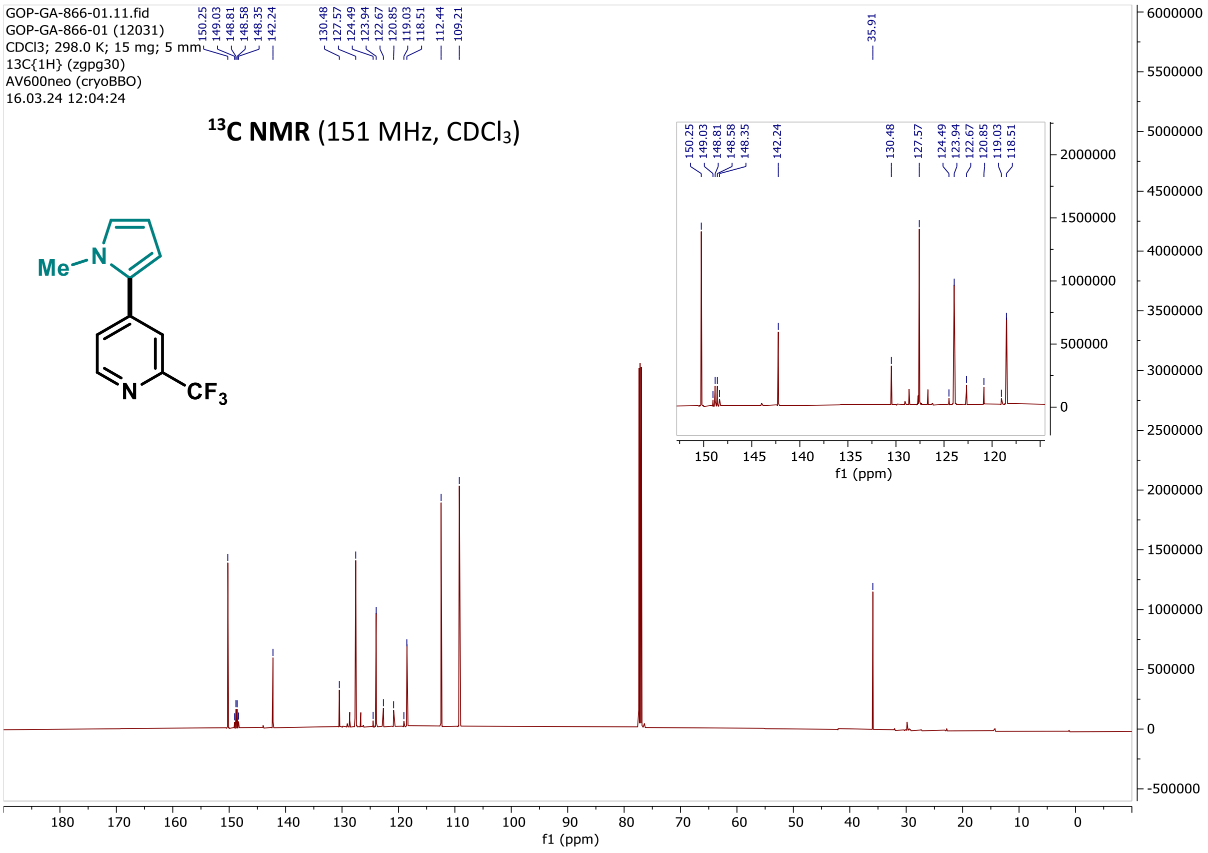

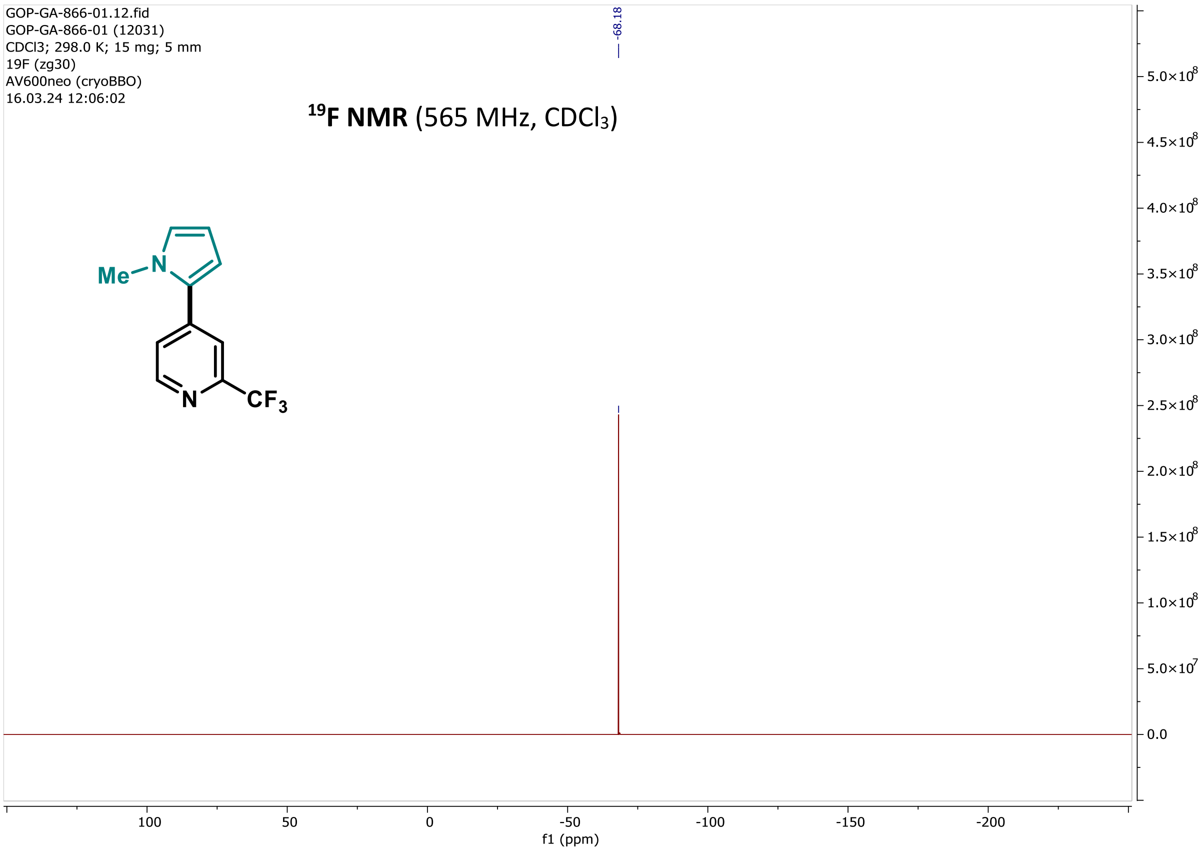
GOP-GA-866-01.12.fid
GOP-GA-866-01 (12031)
CDCl3; 298.0 K; 15 mg; 5 mm
19F (zg30)
AV600neo (cryoBBO)
16.03.24 12:06:02
19F NMR (565 MHz, CDCl3)
-68.18
Me
N
N
CF3
5.0×10^8
4.5×10^8
4.0×10^8
3.5×10^8
3.0×10^8
2.5×10^8
2.0×10^8
1.5×10^8
1.0×10^8
5.0×10^7
0.0
100
50
0
-50
-100
-150
-200
f1 (ppm)

2-(1-Methyl-1*H*-pyrrol-2-yl)pyrazine (5i)

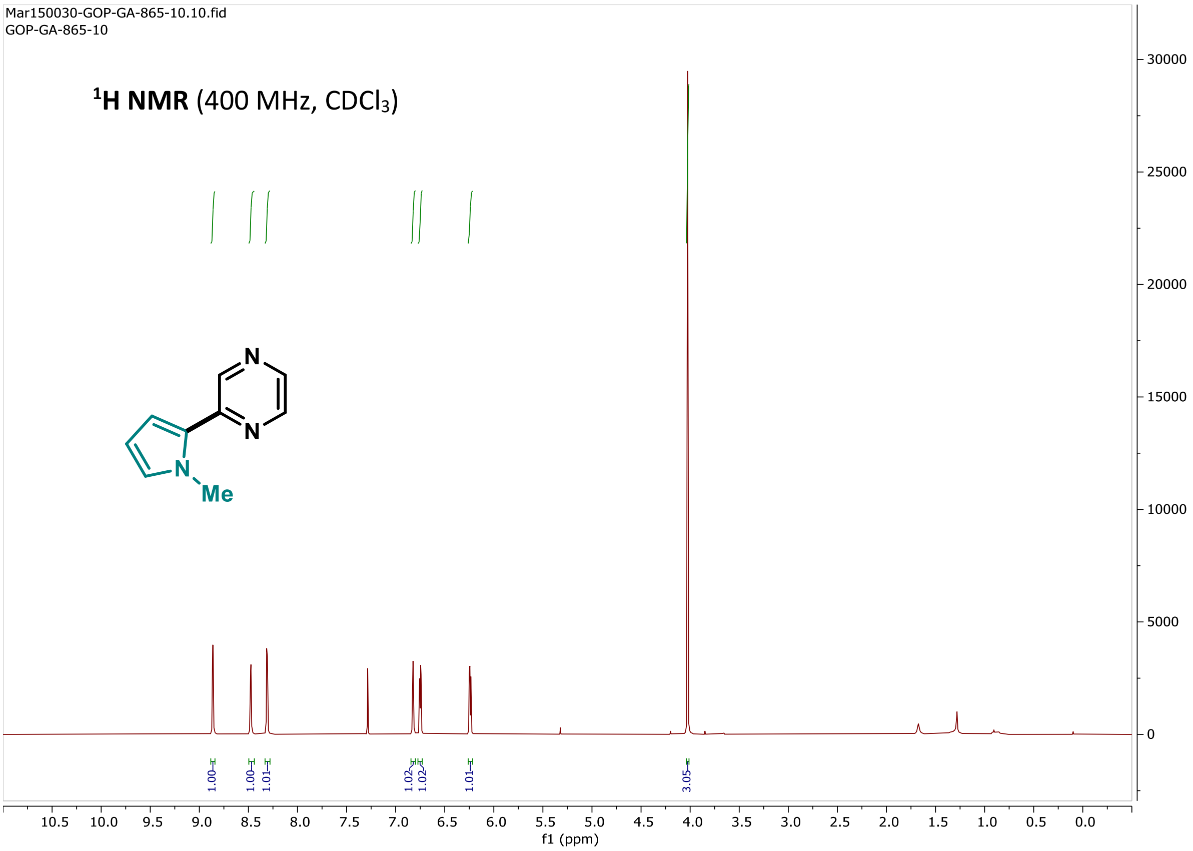

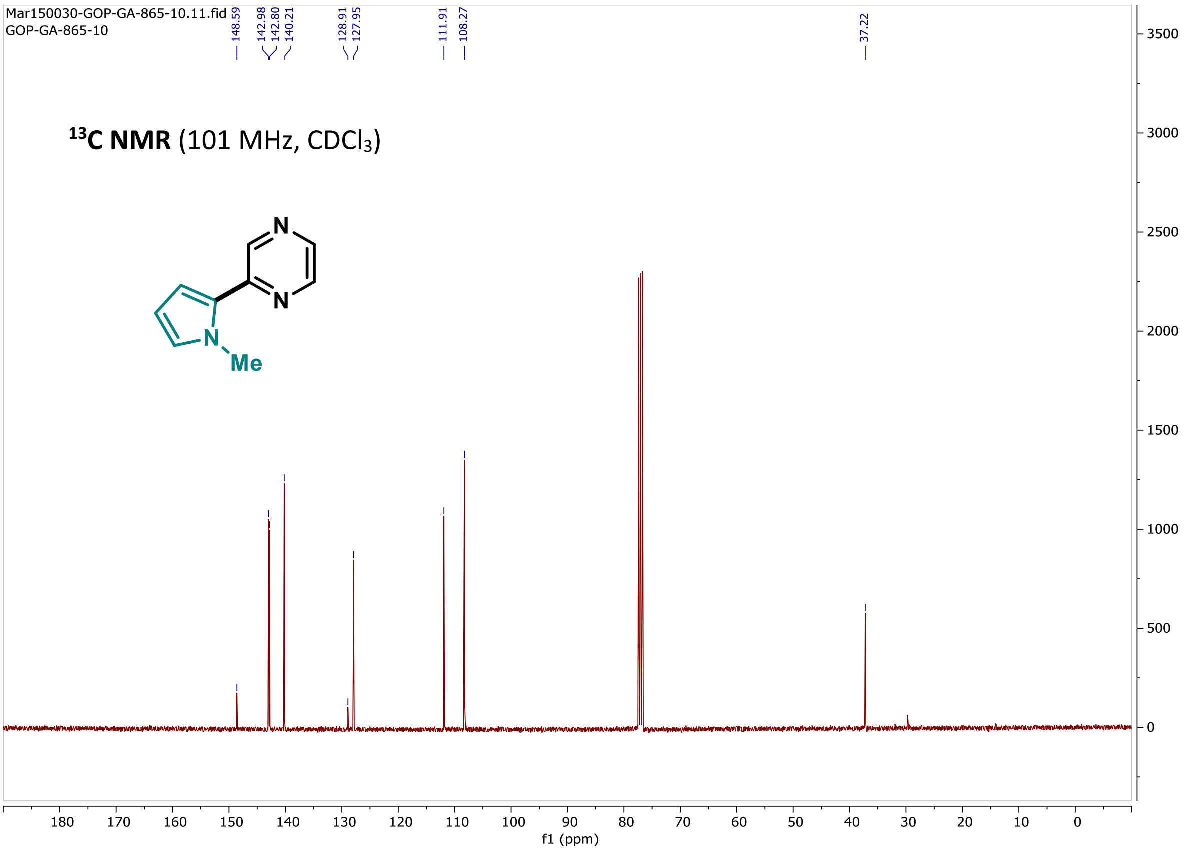

2-(1-Methyl-1*H*-pyrrol-2-yl)pyridine (5j)

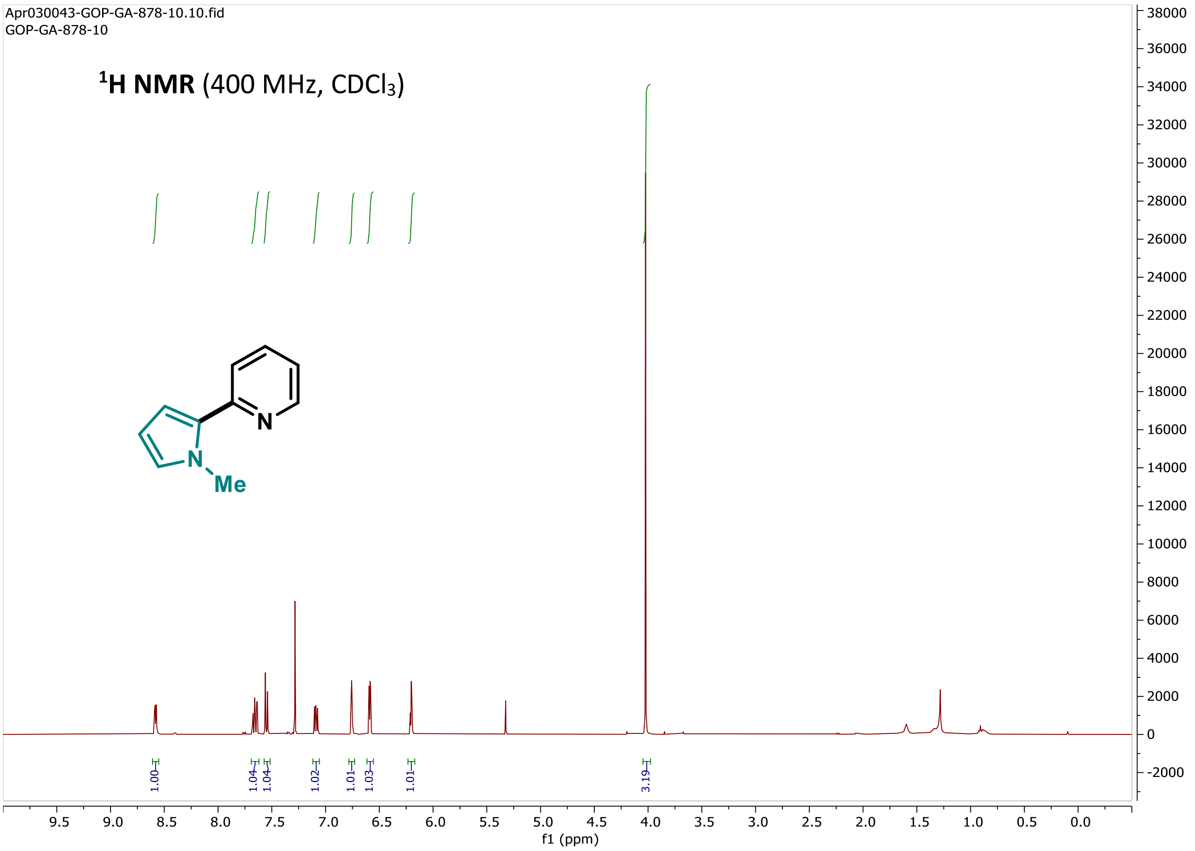

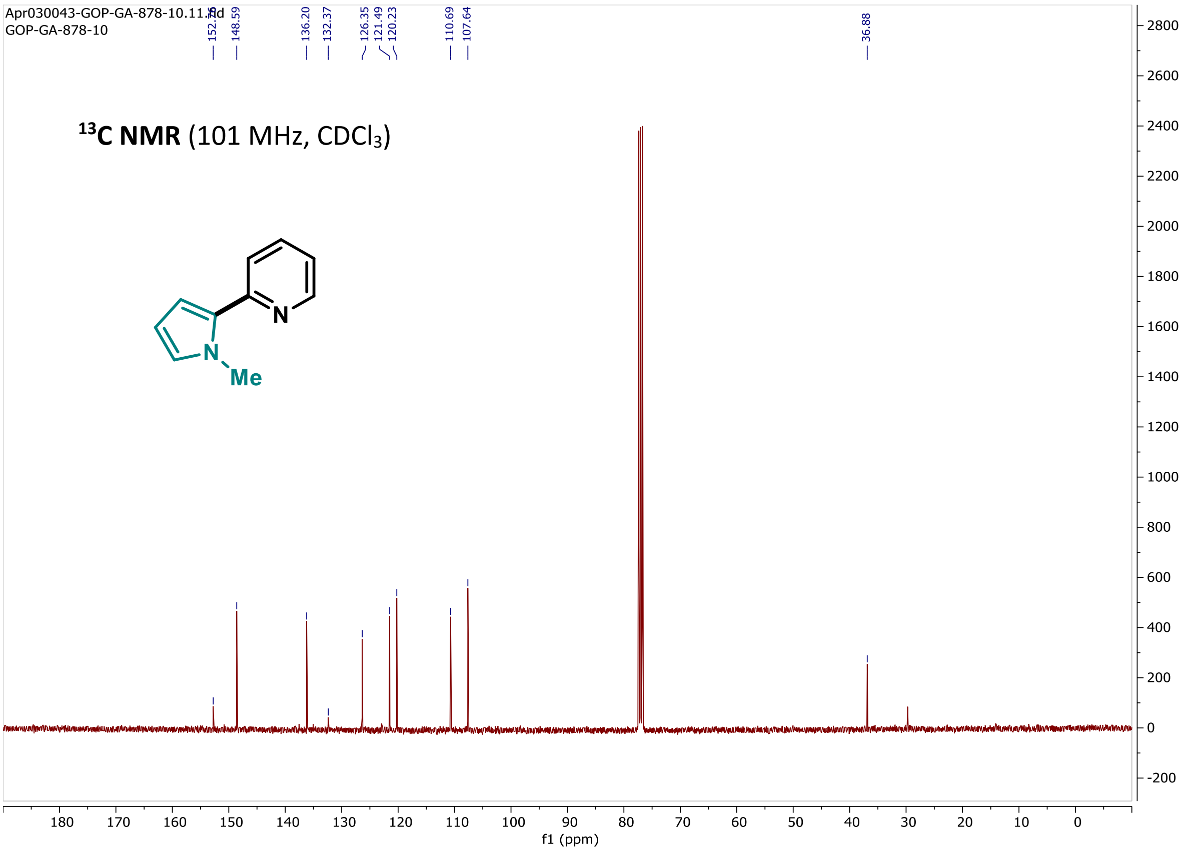

5-Bromo-2-(1-methyl-1*H*-pyrrol-2-yl)pyridine (5k)

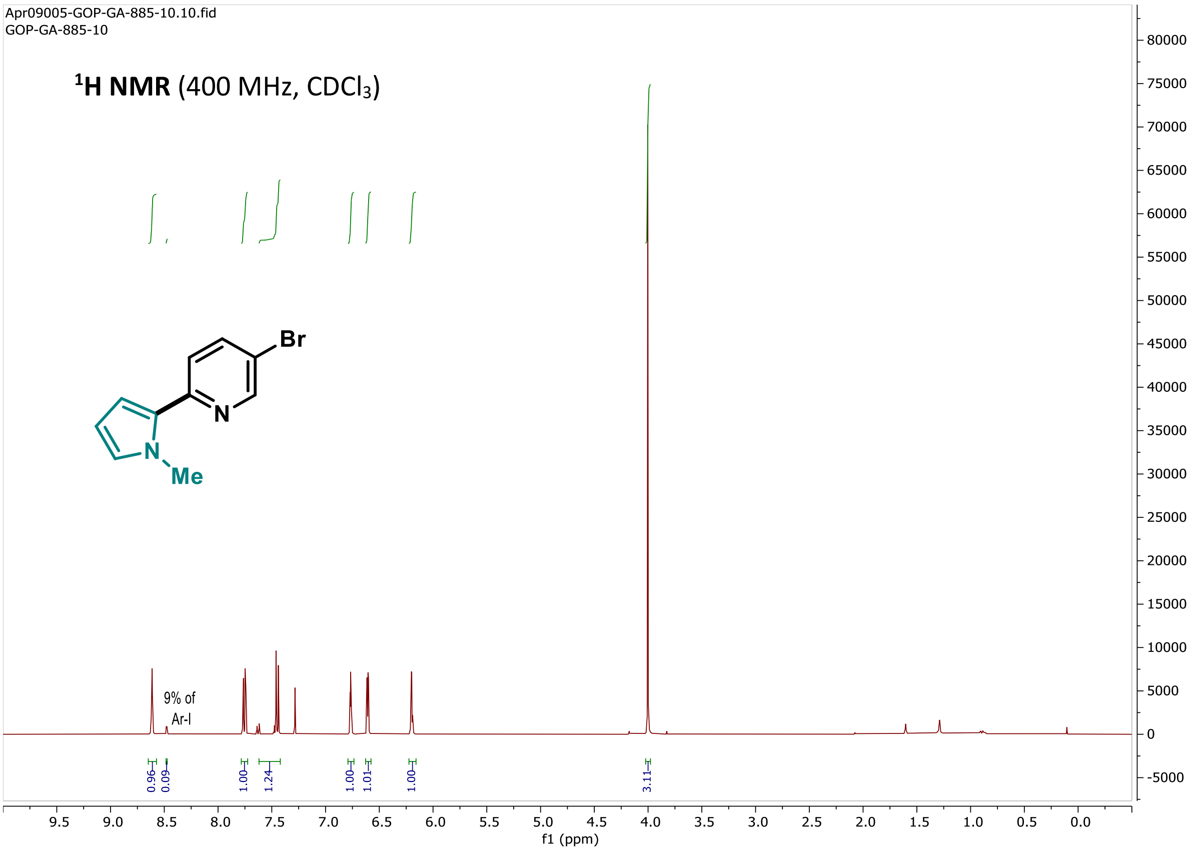

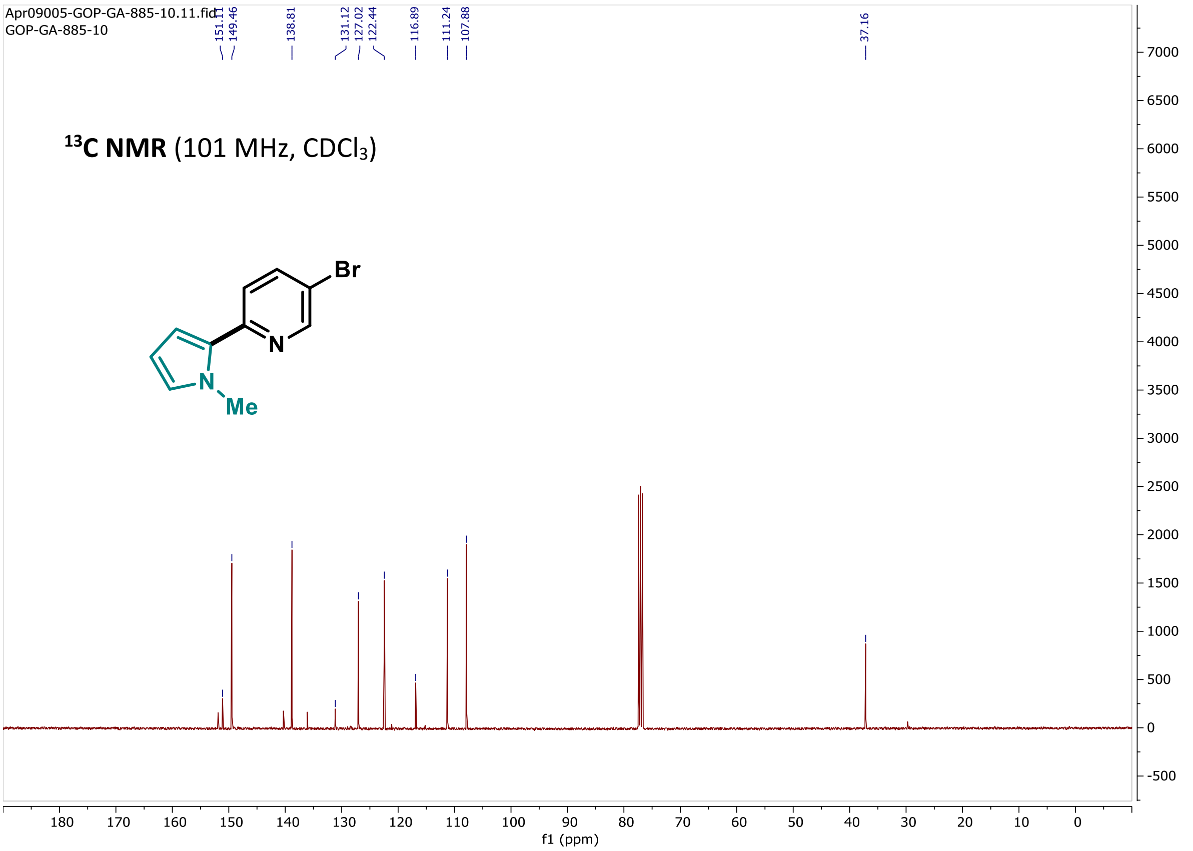

* Contains 9% of starting heteroaryl iodide. Isolated yield corrected accordingly.

2-Bromo-5-(1-methyl-1*H*-pyrrol-2-yl)pyridine (5l)

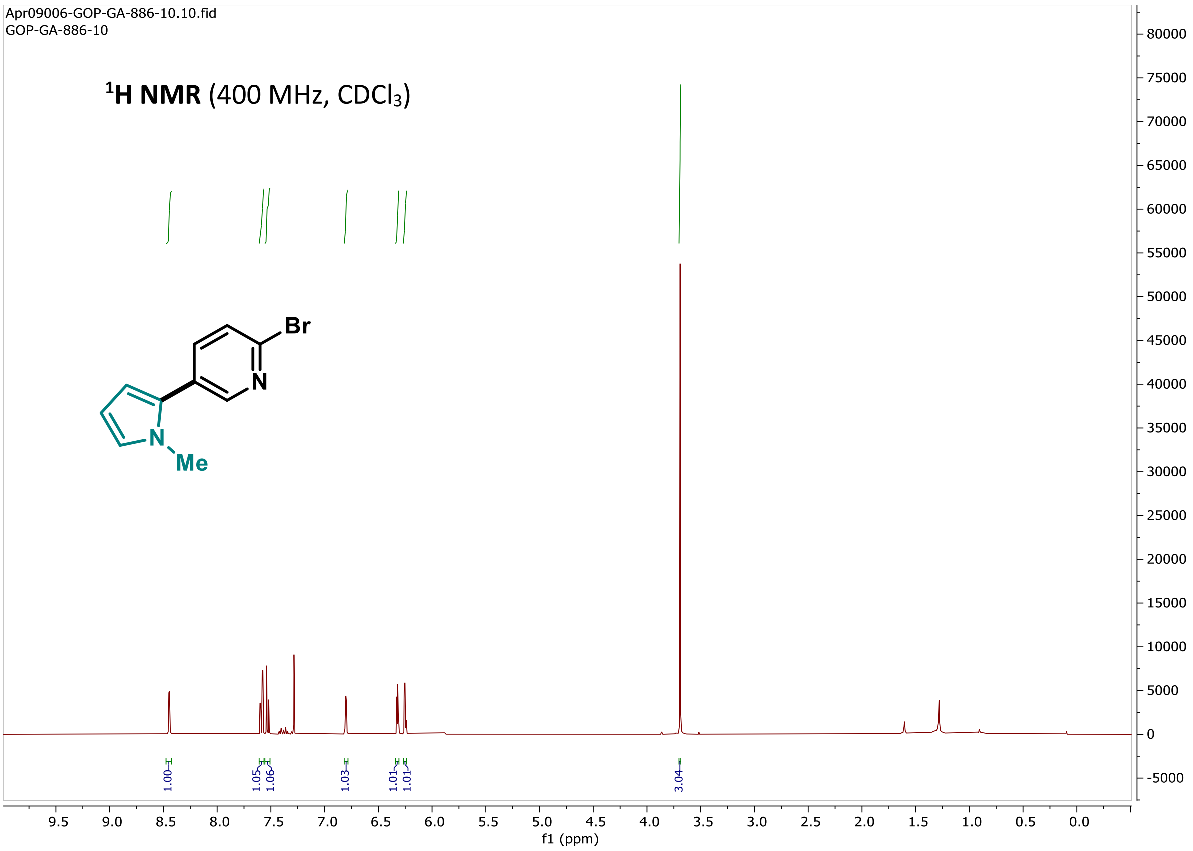

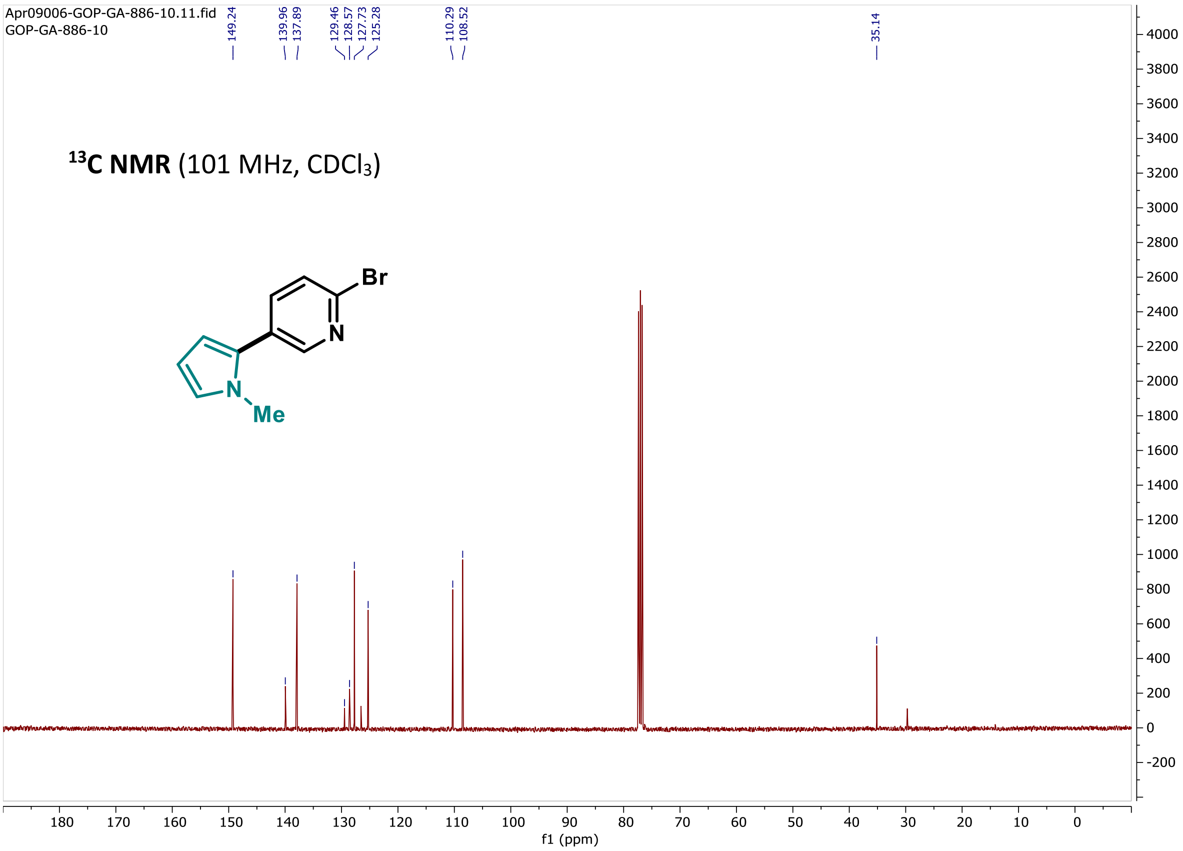

7-Chloro-4-(1-methyl-1*H*-pyrrol-2-yl)quinoline (5m)

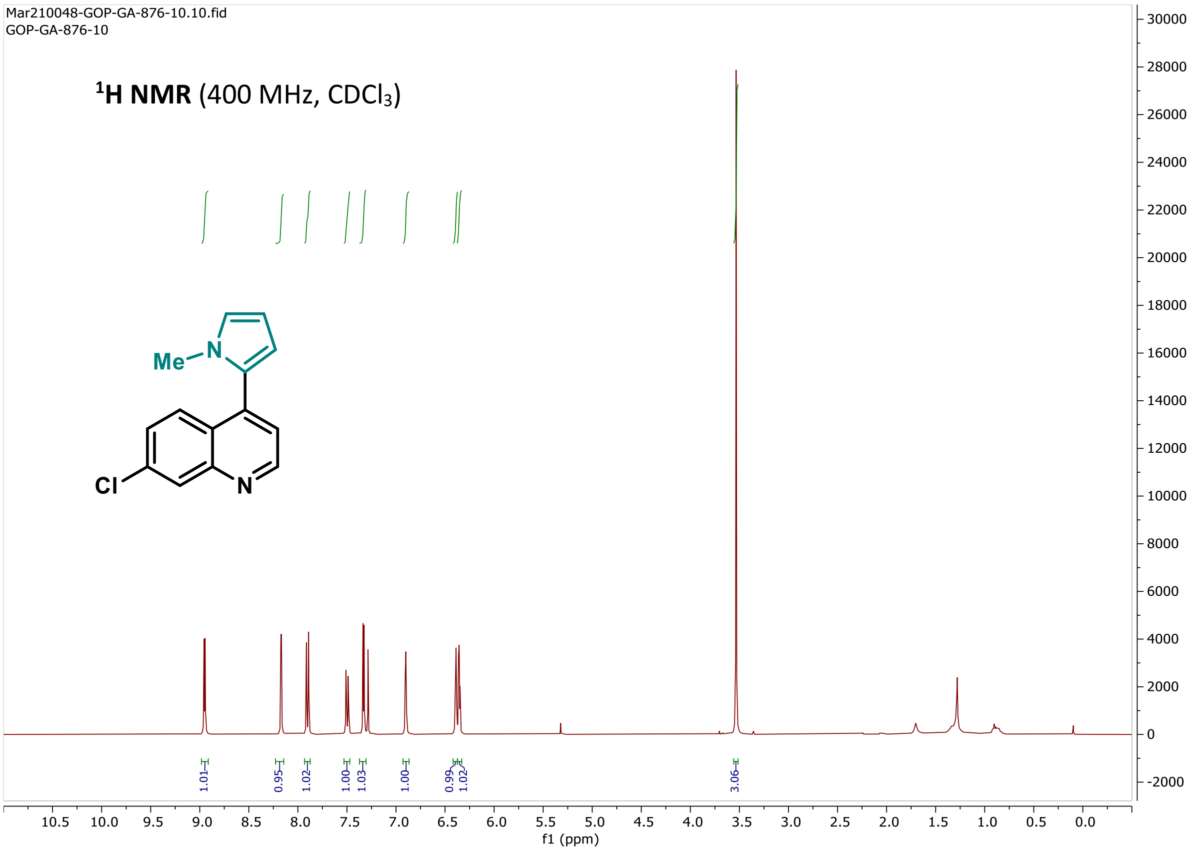

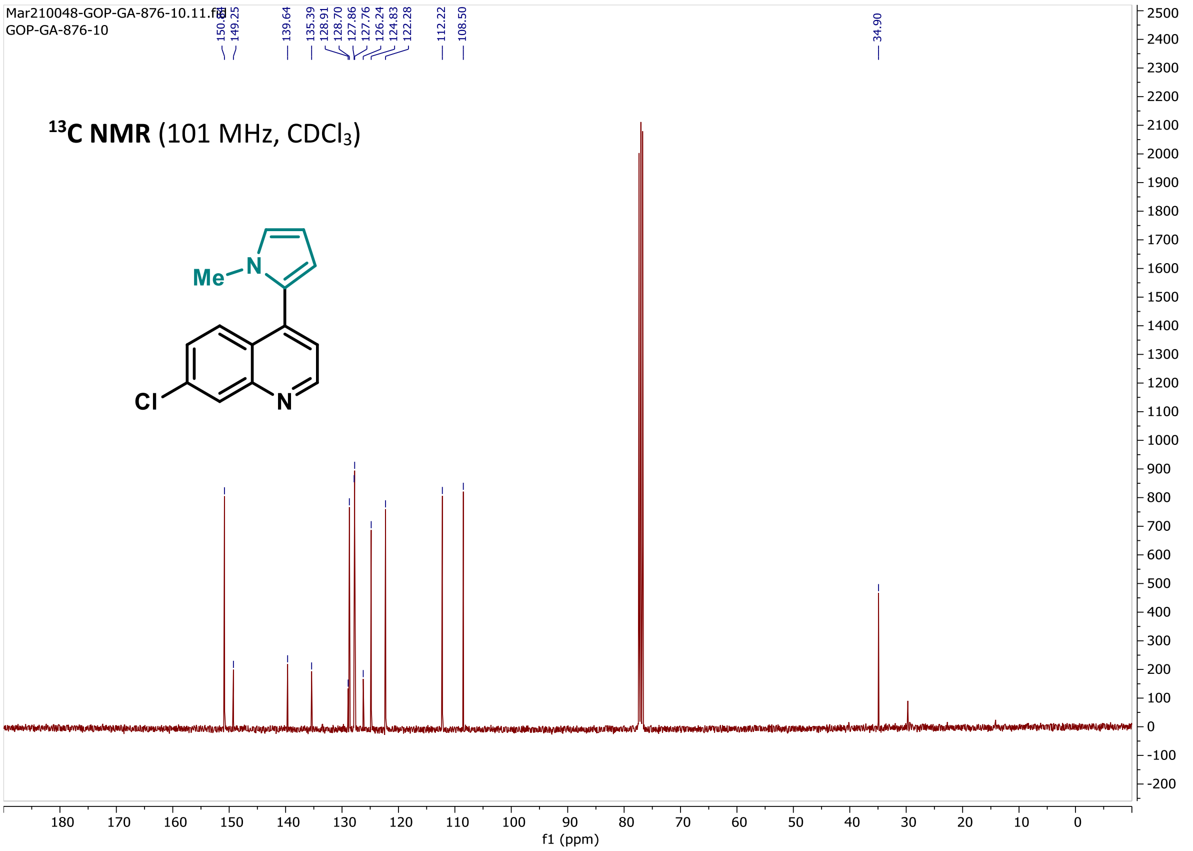

2-(1-Methyl-1*H*-pyrrol-2-yl)quinoxaline (5n)

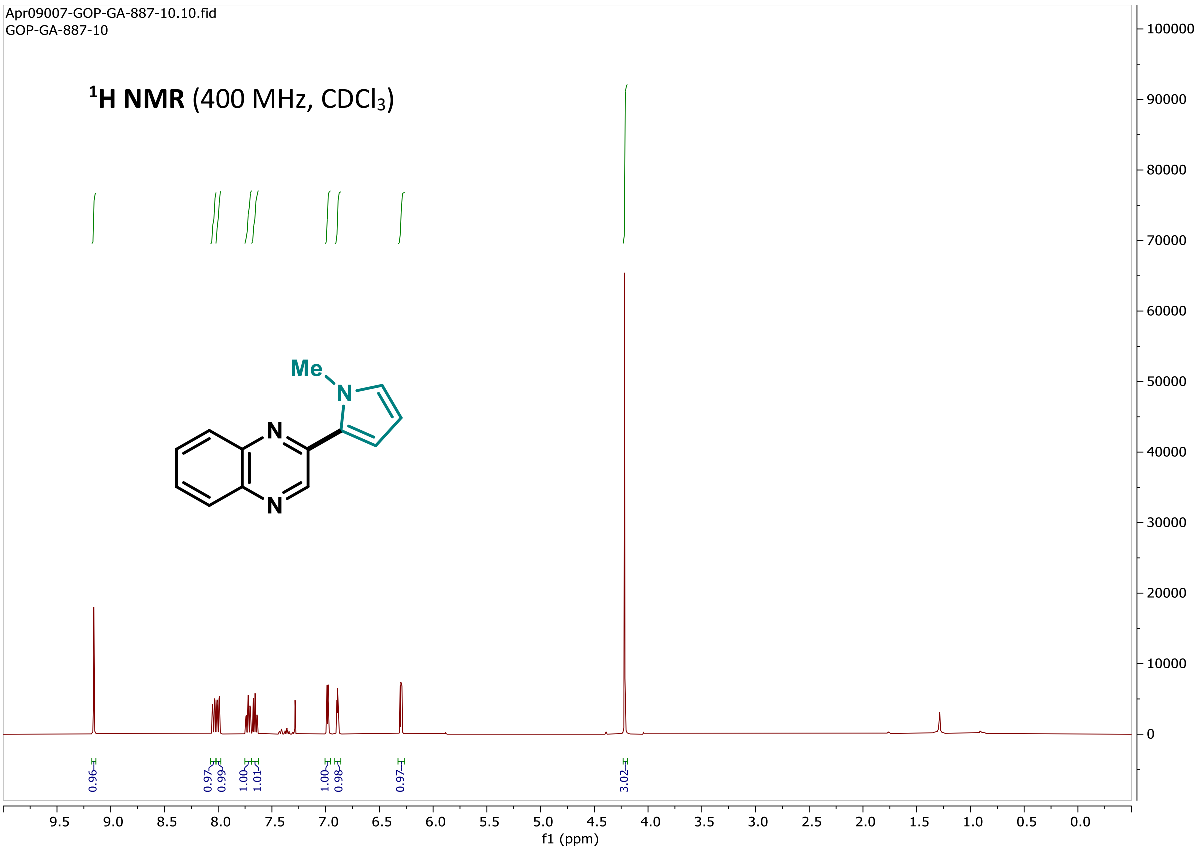

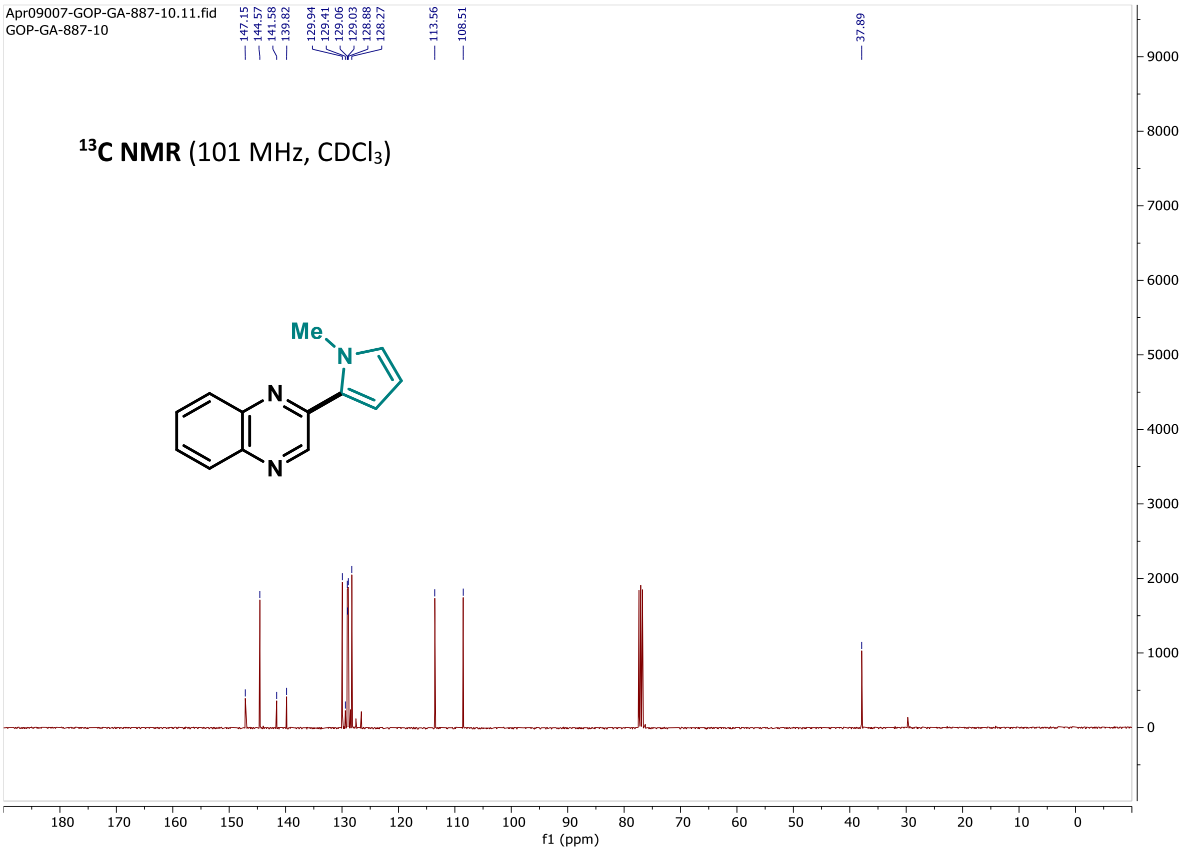

4-(3,5-Dimethyl-1*H*-pyrrol-2-yl)benzonitrile (5o)

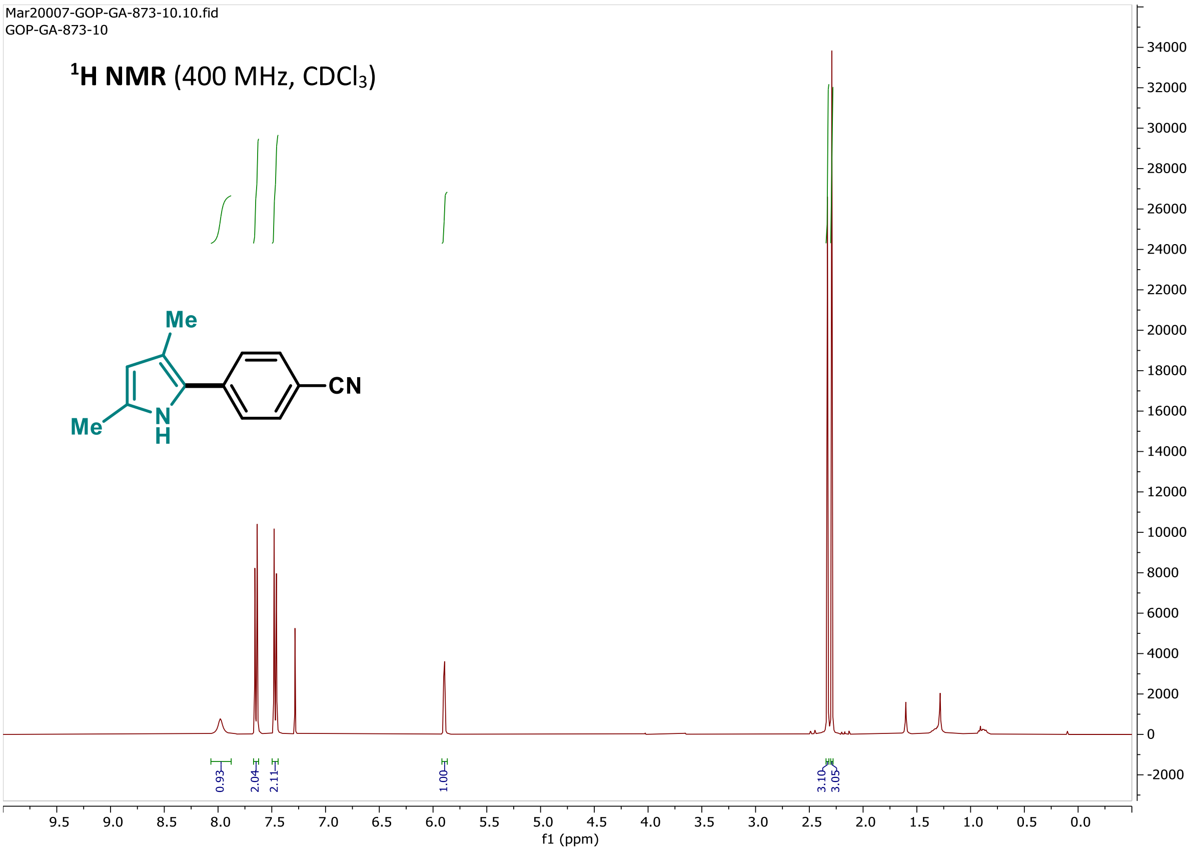

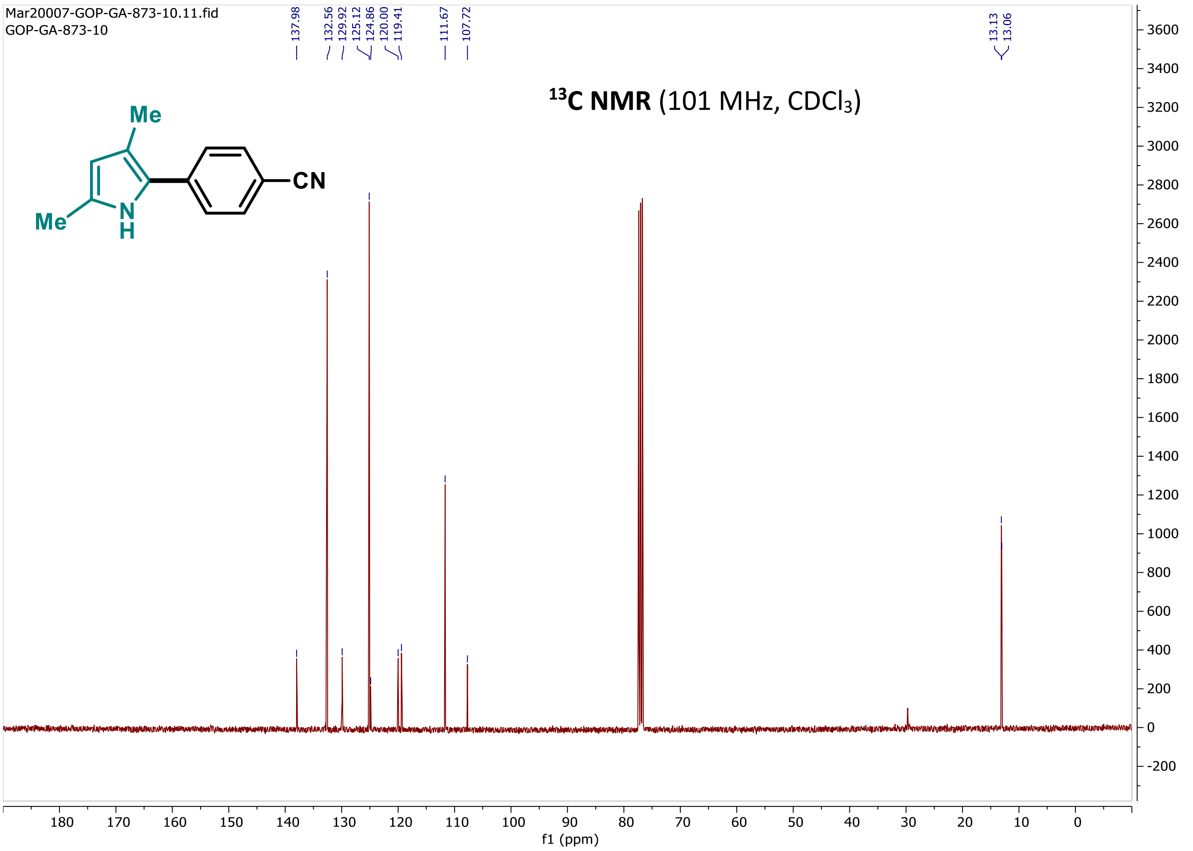

4-(1*H*-Pyrrol-2-yl)benzonitrile (5p)

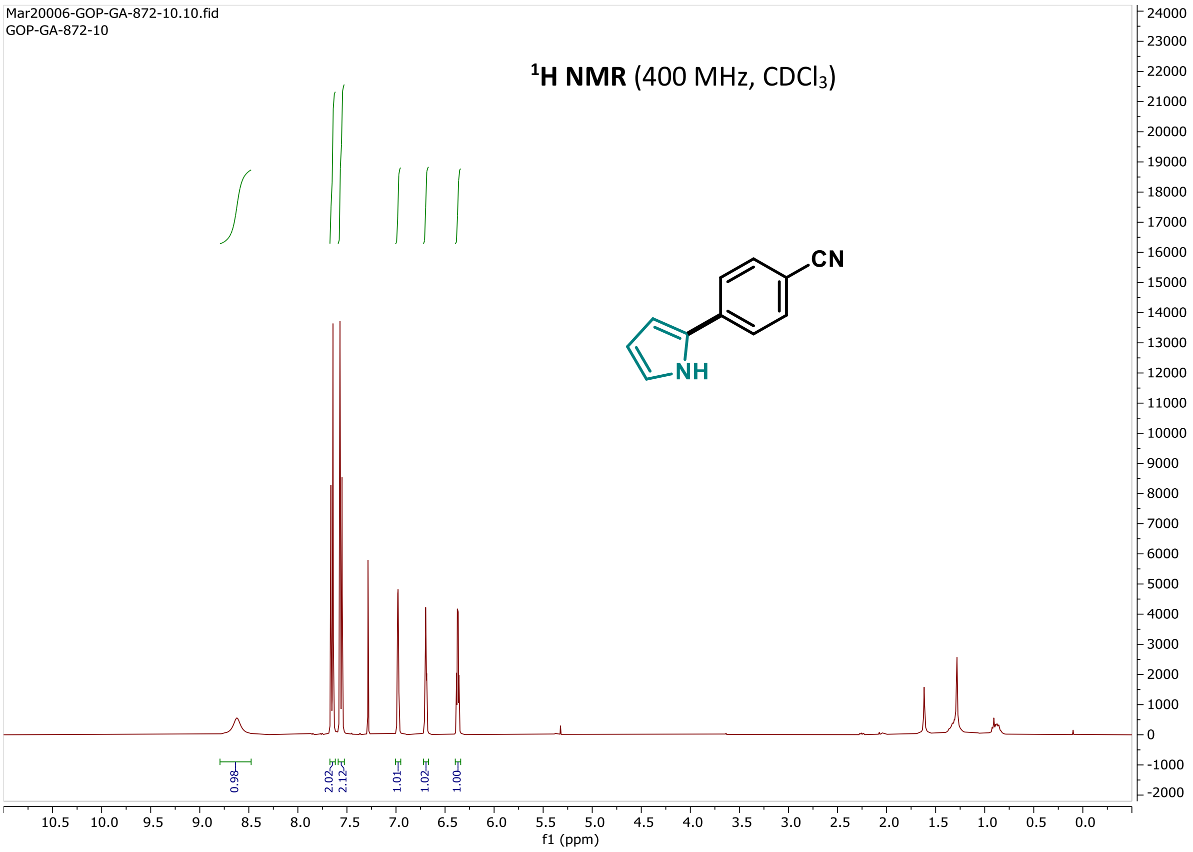

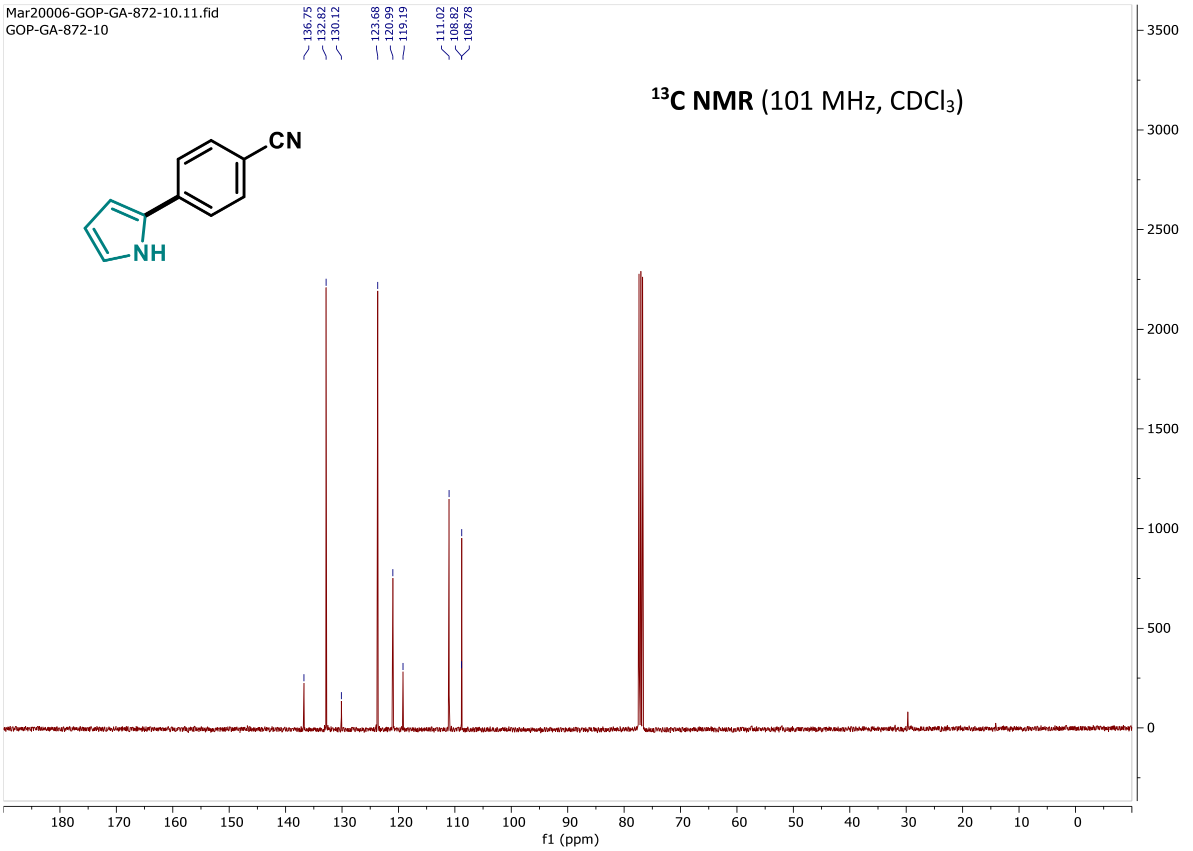

12. References

1. I. Vránová, M. Alonso, R. Lo, R. Sedlák, R. Jambor, A. Růžička, F. Dr Proft, P. Hobza, L. Dostál, From Dibismuthenes to Three- and Two-Coordinated Bismuthinidenes by Fine Ligand Tuning: Evidence for Aromatic BiC3N Rings through a Combined Experimental and Theoretical Study. *Chem. Eur. J.* **2015**, *21*, 16917–16928.

2. F. Wang, O. Planas, J. Cornella, Bi(I)-Catalyzed Transfer-Hydrogenation with Ammonia-Borane. *J. Am. Chem. Soc.* **2019**, *141*, 4235–4240.

3. M. Mato, D. Spinnato, M. Leutzsch, H. –W. Moon, E. J. Reijerse, J. Cornella, Bismuth radical catalysis in the activation and coupling of redox-active electrophiles. *Nat. Chem.* **2023**, *15*, 1138–1145.

4 T. Tsuruta, D. Spinnato, H. –W. Moon, M. Leutzsch, J. Cornella, Bi-Catalyzed Trifluoromethylation of C(sp^2)–H Bonds under Light. *J. Am. Chem. Soc.* **2023**, *145*, 25538–25544.

5 M. Mato, P. C. Bruzzese, F. Takahashi, M. Leutzsch, E. J. Reijerse, A. Schnegg, J. Cornella Oxidative Addition of Aryl Electrophiles into a Red-Light-Active Bismuthinidene. *J. Am. Chem. Soc.* **2023**, *145*, 18742–18747.

6. M. Bremerich, C. M. Conrads, T. Langletz, C. Bolm, Additions to N-Sulfinylamines as an Approach for the Metal-free Synthesis of Sulfonimidamides: *O*-Benzotriazolyl Sulfonimidates as Activated Intermediates. *Angew. Chem. Int. Ed.* **2019**, *58*, 19014–19020.

7 A. P. Soran, C. Silvestru, H. J. Breunig, G. Balázs, J. C. Green, Organobismuth(III) Dihalides with T-Shaped Geometry Stabilized by Intramolecular N→Bi Interactions and Related Diorganobismuth(III) Halides. *Organometallics* **2007**, *26*, 1196–1203.

8 M. Hejda, R. Jirásko, A. Růžička, R. Jambor, L. Dostál, Probing the Limits of Oxidative Addition of C(sp^3)–X Bonds toward Selected *N,C,N*-Chelated Bismuth(I) Compounds. *Organometallics* **2020**, *39*, 4320–4328.

9 (a) C. G. Hatchard, C. A. Parker, A new sensitive chemical actinometer - II. Potassium ferrioxalate as a standard chemical actinometer. Proc. Roy. Soc. (London) **1956**, *A235*, 518–536. (b) M. A. Cismesia, T. P. Yoon, Characterizing chain processes in visible light photoredox catalysis. *Chem. Sci.* **2015**, *6*, 5426–5434. (c) X. Zhao, S. Zhu, F. –L. Qing, L. Chu, Reductive hydrobenzylation of terminal alkynes via photoredox and nickel dual catalysis. *Chem. Commun.* **2021**, *57*, 9414–9417.

10 F. Neese, Software update: The ORCA program system—Version 5.0. *WIREs Comput. Mol. Sci.* **2022**, *12*, e1606.

11 W. Kutzelnigg, W. Liu, Quasirelativistic theory equivalent to fully relativistic theory. *J. Chem. Phys.* **2005**, *123*, 241102.

12 (a) A. D. Becke, Density-functional thermochemistry. III. The role of exact Exchange. *J. Chem. Phys.* **1993**, *98*, 5648–5652. (b) C. Lee, W. Yang, R. G. Parr, Development of the Colle-Salvetti correlation-energy formula into a functional of the electron density. *Phys. Rev. B* **1988**, *37*, 785.

13 (a) P. Pollak, F. Weigend, Segmented Contracted Error-Consistent Basis Sets of Double- and Triple-ζ Valence Quality for One- and Two-Component Relativistic All-Electron Calculations. *J. Chem. Theory Comput.* **2017**, *13*, 3696–3705. (b) Y. J. Franzke, R. Treß, T. M. Pazdera, F. Weigend, Error-consistent segmented contracted all-electron relativistic

basis sets of double- and triple-zeta quality for NMR shielding constants. *Phys. Chem. Chem. Phys.* **2019**, *21*, 16658–16664.

14 (a) S. Grimme, J. Antony, S. Ehrlich, H. Krieg, A consistent and accurate ab initio parametrization of density functional dispersion correction (DFT-D) for the 94 elements H-Pu. *J. Chem. Phys.* **2010**, *132*, 154104. (b) S. Grimme, S. Ehrlich, L. Goerigk, Effect of the damping function in dispersion corrected density functional theory. *J. Comput. Chem.* **2011**, *32*, 1456–1465.

15 A. V. Marenich, C. J. Cramer, D. G. Truhlar, Universal Solvation Model Based on Solute Electron Density and on a Continuum Model of the Solvent Defined by the Bulk Dielectric Constant and Atomic Surface Tensions. *J. Phys. Chem. B* **2009**, *113*, 6378–6396.

16 (a) B. A. Heß, C. M. Marian, U. Wahlgren, O. Gropen, A mean-field spin-orbit method applicable to correlated wavefunctions. *Chem. Phys. Lett.* **1996**, *251*, 365–371. (b) F. Neese, Efficient and accurate approximations to the molecular spin-orbit coupling operator and their use in molecular *g*-tensor calculations. *J. Chem. Phys.* **2005**, *122*, 034107.

17 (a) F. Neese, A. Hansen, D. G. Liakos, Efficient and accurate approximations to the local coupled cluster singles doubles method using a truncated pair natural orbital basis. *J. Chem. Phys.* **2009**, *131*, 064103. (b) F. Neese, F. Wennmohs, A. Hansen, Efficient and accurate local approximations to coupled-electron pair approaches: An attempt to revive the pair natural orbital method. *J. Chem. Phys.* **2009**, *130*, 114108. (c) C. Riplinger, F. Neese, An efficient and near linear scaling pair natural orbital based local coupled cluster method. *J. Chem. Phys.* **2013**, *138*, 034106. (d) G. Bistoni, C. Riplinger, Y. Minenkov, L. Cavallo, A. A. Auer, F. Neese, Treating Subvalence Correlation Effects in Domain Based Pair Natural Orbital Coupled Cluster Calculations: An Out-of-the-Box Approach. Treating Subvalence Correlation Effects in Domain Based Pair Natural Orbital Coupled Cluster Calculations: An Out-of-the-Box Approach. *J. Chem. Theory Comput.* **2017**, *13*, 3220–3227. (e) M. Saitow, U. Becker, C. Riplinger, E. F. Valeev, F. Neese, A new near-linear scaling, efficient and accurate, open-shell domain-based local pair natural orbital coupled cluster singles and doubles theory. *J. Chem. Phys.* **2017**, *146*, 164105.
